# Next‐Generation Piezoelectric Materials in Wearable and Implantable Devices for Continuous Physiological Monitoring

**DOI:** 10.1002/advs.202507853

**Published:** 2025-09-25

**Authors:** Bangul Khan, Umay Amara, Bilawal Khan, Wasim Ullah Khan, Rafi u Shan Ahmad, Muhammad Shehzad Khan, Mohamed Elhousseini Hilal, Bee Luan Khoo

**Affiliations:** ^1^ Department of Biomedical Engineering, College of Biomedicine City University of Hong Kong Tat Chee Ave Kowloon Hong Kong China; ^2^ Hong Kong Centre of Cerebro‐Cardiovascular Health Engineering (COCHE) Shatin Hong Kong China; ^3^ School of Materials Science and Engineering Anhui University Hefei 230601 China; ^4^ Department of Materials Science and Engineering City University of Hong Kong Kowloon Hong Kong China; ^5^ Futian Shenzhen Research Institute City University of Hong Kong Kowloon Hong Kong China

**Keywords:** Health Monitoring, Implantable Devices, Piezoelectric Materials, Wearable Devices

## Abstract

The rapid expansion of miniature biomedical devices has attracted considerable global attention, driven by the growing demand for advanced healthcare solutions. Despite significant progress in materials, fabrication techniques, and device architectures that have propelled the development of wearable and implantable technologies, a critical challenge remains: mimicking the structure and function of human skin. This challenge contrasts advancements in power efficiency, design miniaturization, precision, and device integration. Recent breakthroughs in manufacturing techniques and the development of high‐performance organic and inorganic piezoelectric materials with tunable mechanical properties offer transformative potential to overcome these limitations. This review systematically examines the evolution of piezoelectric materials for health monitoring applications, focusing on their historical development, underlying mechanisms, fabrication strategies, and characterization techniques. We critically evaluate their integration into wearable and implantable systems, emphasizing their potential to address power autonomy, device adaptability, and sensing accuracy issues. Additionally, the article highlights emerging interdisciplinary research frontiers in bioengineering, highlighting pioneering contributions from global research teams. By synthesizing key advancements and identifying unresolved challenges, this review aims to provide a comprehensive guide for future innovations in smart biomedical devices, fostering collaborative efforts across the materials science, electronics, and healthcare fields.

## Graphical abstract

An analysis of literature trends and a historical overview of organic and inorganic piezoelectric materials, focusing on their structural diversity, functional mechanisms, and inherent characteristics. It then explores cutting‐edge developments in material synthesis, fabrication processes, and performance optimization, highlighting their applicability in biomedical technologies such as wearables and implants. The subfigures are reprinted with copyright permissions, which are mentioned in the respective section, while the Quartz structure is adapted under CC. BY license 4.0.

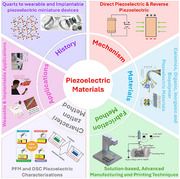

## 1 Introduction

Implantable and wearable devices have recently garnered significant global attention, primarily driven by the increased public demand for real‐time, continuous health monitoring in an aging population and the increasing prevalence of deadly diseases.^[^ ^1^, ^2^ ^]^ This opens a new arena in the field of bioengineering, with a focus on developing biocompatible, miniaturized, flexible and self‐powered systems capable of seamless integration with the human body.^[^ ^3^, ^4^, ^5^ ^]^ Among various engineering challenges, replicating the mechanical and structural characteristics of human skin remains one of the most complex challenge.^[^ ^6^ ^]^ Unlike traditional challenges such as miniaturization, power supply, or signal fidelity, the ability to match the flexibility, stretchability, and multimodal sensing properties of the skin is crucial for ensuring comfort, minimizing immune responses, and enhancing long‐term device‐tissue integration.^[^ ^7^, ^8^ ^]^

To address this, significant research has been devoted to designing functional materials that not only mimic skin‐like properties but also enable self‐powered operation through energy harvesting.^[^ ^9^ ^]^ In this context, piezoelectric materials which are capable of converting mechanical deformations into electrical signals are promising solutions.^[^ ^10^ ^]^ These materials are broadly classified into inorganic and organic piezoelectric materials.^[^ ^11^ ^]^ High‐performance inorganic materials such as lead zirconate titanate (PZT),^[^ ^12^ ^]^ lithium niobate (LiNbO₃),^[^ ^13^ ^]^ barium titanate (BaTiO₃),^[^ ^14^ ^]^ zinc oxide (ZnO),^[^ ^15^ ^]^ and quartz^[^ ^16^ ^]^ possess strong piezoelectric properties due to their noncentrosymmetric crystal structures, which facilitate ionic displacement under mechanical stress. However, their brittleness, rigidity, and potential toxicity raise concerns for implantable and flexible applications.^[^ ^17^ ^]^

In contrast, organic piezoelectric materials—predominantly polymers such as polyvinylidene fluoride (PVDF),^[^ ^18^ ^]^ poly(L‐lactic acid) (PLLA),^[^ ^19^ ^]^ and poly(D‐lactic acid)^[^ ^20^ ^]^—exhibit intrinsic biocompatibility, biodegradability, and mechanical flexibility.^[^ ^21^, ^22^ ^]^ Their piezoelectricity arises from their molecular dipole orientation in semicrystalline phases.^[^ ^23^ ^]^ Although these polymers typically display lower piezoelectric coefficients than inorganic piezomaterials but they offer advantages in terms of low cost, ease of processing, and environmental sustainability.^[^ ^17^ ^]^ Additionally, piezoelectricity has also been observed in biological tissues and biomolecules,^[^ ^24^ ^]^ including collagen,^[^ ^25^ ^]^ keratin,^[^ ^26^ ^]^ and various polysaccharides, presenting opportunities for biomimetic sensor development.^[^ ^27^ ^]^

Recent advances in materials synthesis, nanofabrication, and biointegrated device engineering have accelerated the design of piezoelectric systems with tunable shapes, properties, and functionalities.^[^ ^28^ ^]^ These include stretchable energy harvesters,^[^ ^29^ ^]^ flexible pressure sensors, tissue‐interfaced actuators,^[^ ^32^ ^]^ and implantable therapeutic systems.^[^ ^2^, ^33^ ^]^ Despite these breakthroughs, several critical challenges remain unresolved: achieving stable performance in dynamic physiological environments, increasing the output power density of organic materials, and ensuring long‐term biostability and immunocompatibility.^[^ ^34^ ^]^ Moreover, the integration of multifunctional piezoelectric systems that can simultaneously sense, harvest energy, and actuate remains an open frontier.^[^ ^35^ ^]^

Several review articles reported in recent years, such as Wang et al.^[^ ^36^ ^]^ summarized the development and application of self‐powered wearable monitoring sensors based on piezoelectric materials and composites for healthcare, especially in the context of the post‐COVID‐19 era. Huang et al.^[^ ^37^ ^]^ documented the development of flexible, wearable piezoelectric cardiac sensors enhanced by machine learning for continuous and real‐time heart monitoring. Li et al.^[^ ^38^ ^]^ reviewed the biomedical applications of 3D‐printed piezoelectric composites, highlighting their potential in wearable and implantable medical devices. However, this review focuses on the developmental trajectory of piezoelectric materials for miniature wearable and implantable biomedical devices, addressing the challenge of mimicking the complex properties of human skin. It highlights recent advances in material design, fabrication techniques, and device integration, aiming to improve power autonomy, adaptability, and sensing accuracy. The review also emphasizes interdisciplinary innovations and global research efforts, offering a comprehensive guide for future smart healthcare technologies.

The structure of this review is as follows. It begins by highlighting the evolution and history of various structures, mechanisms, characteristics, and configurations of organic and inorganic piezoelectric materials. This thorough exploration further summarizes recent advancements in their synthesis techniques, processing techniques, and performance characterization, with potential applications in wearable and implantable biomedical devices. This review delves into a comprehensive summary of wearable and implantable devices for physiological monitoring in the last five years by different research groups. The conclusion of the discourse converges upon a reflective discussion and conclusive interpretations that outline the future direction of piezoelectric material advancements, conceivable challenges, and prospective viewpoints in healthcare monitoring.

## 2 History of Piezoelectric Materials

The history of piezoelectric materials began in 1880 when the Curie brothers discovered that certain crystals, such as quartz and Rochelle salt, generate electric charges under mechanical pressure—a phenomenon they named piezoelectricity.^[^ ^39^ ^]^ However, Curie's brother only acknowledged the direct piezoelectric effect and was unaware of the existence of the inverse piezoelectric effect. In 1881, Gabriel Lippmann predicted the inverse piezoelectric effect—where an electric field induces mechanical strain—on the basis of thermodynamic principles, which the Curie brothers later confirmed experimentally. The first practical application occurred in 1917 during World War I, when Paul Langevin used piezoelectric materials in ultrasonic submarine detectors. Since then, piezoelectric technology has expanded globally, influencing various fields beyond healthcare.^[^ ^40^, ^41^, ^42^ ^]^

## 3 Piezoelectric Mechanism

**Figure 1:**
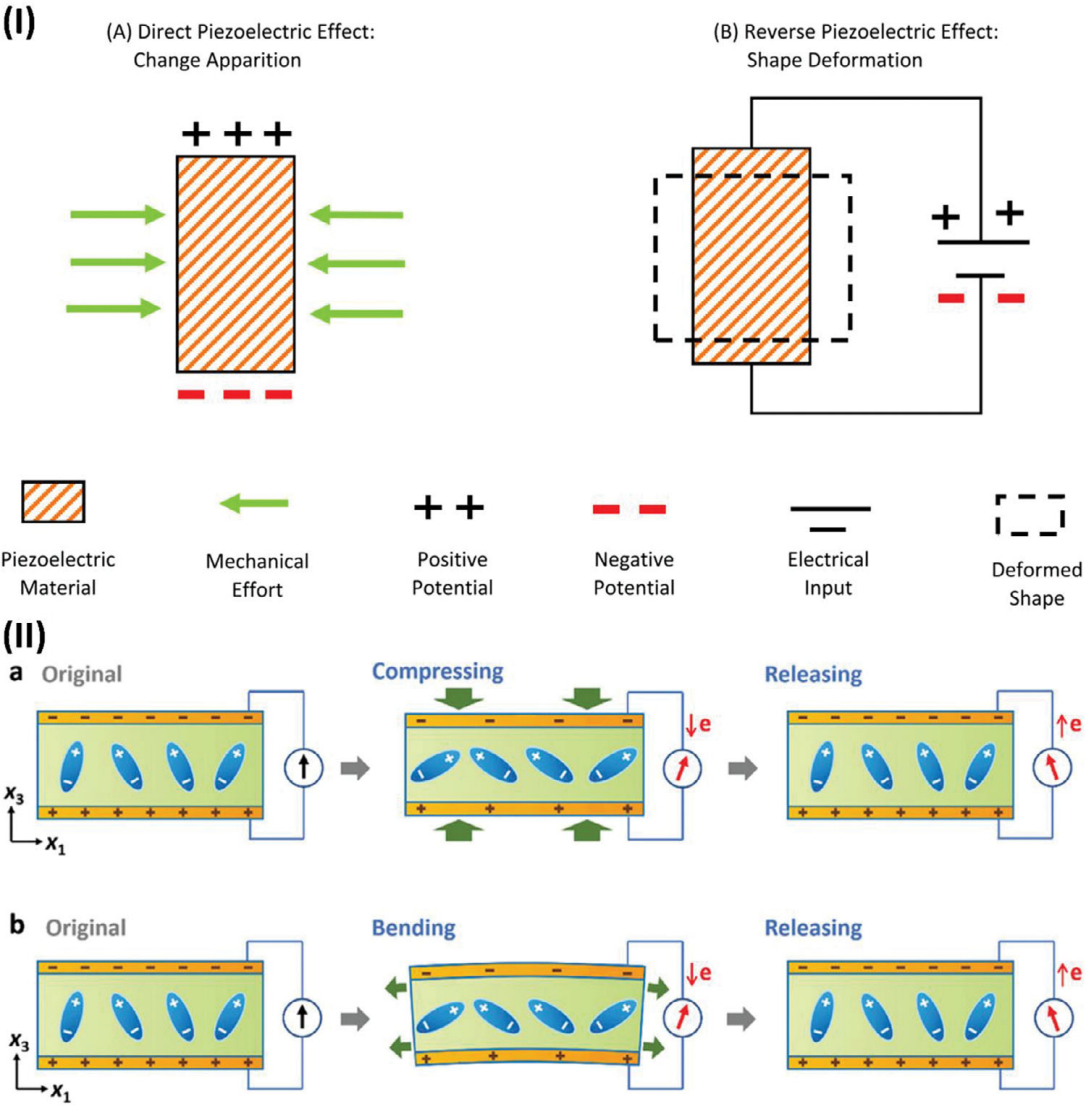
Piezoelectric mechanism illustration. I) Electromechanical conversion of piezoelectric phenomena reproduced under a CC BY license.^[^ ^43^ ^]^ Copyright 2020, Elsevier Ltd. II) Working modes of piezoelectric devices reproduced under a CC BY license.^[^ ^36^ ^]^ Copyright 2022, Wiley VCH GmbH.

The piezoelectric effect is a two‐way phenomenon. When mechanical pressure is applied to a piezoelectric material, it generates an electric charge—this is known as the direct piezoelectric effect (**Figure** **1**IA). Conversely, when an external electric field is applied, the material undergoes mechanical deformation, which is referred to as the inverse (or indirect) piezoelectric effect (Figure 1IB).^[^ ^43^ ^]^ Initially, piezoelectricity is generated in crystals that possess unit cells that lack symmetry to maintain electrical balance.^[^ ^44^ ^]^ Piezoelectricity depends upon the alignment of the dipole density, the symmetry of the crystal, and the mechanical stress and outer electrical field that is exerted.^[^ ^45^ ^]^ When pressure is applied to a crystal, causing it to compress or expand, deformation of its structure results in atomic displacement. This disrupts the equilibrium between positive and negative charges, leading to the emergence of net electrical charges. This phenomenon persists throughout the entire structure, resulting in positive and negative charges on the outer surfaces of the crystal located on opposite sides.^[^ ^46^ ^]^ The reverse piezoelectric effect is the opposite phenomenon^[^ ^47^ ^]^: applying a voltage to a piezoelectric crystal results in the atoms within the crystal experiencing electrical pressure. The movement required for self‐rebalancing leads to the deformation of piezoelectric crystals when a voltage is placed across them. The electric dipole moments in solids are closely related to the piezoelectric effect.^[^ ^48^, ^49^ ^]^

The working mechanism of piezoelectric devices relies on the polarization of electric dipoles within piezoelectric materials. When no external force is applied, the internal electric field remains balanced, and no current is generated. However, when a mechanical stimulus is applied, the material deforms, altering its crystal structure. This deformation induces dipole polarization, generating a piezoelectric potential and producing an electric current. Once the pressure is removed, the material returns to its original shape, the polarization disappears, and a reverse current is generated, as highlighted in (Figure 1IIa). When the piezoelectric material bends, it experiences lateral strain, reducing its polarization and generating a voltage (Figure 1IIb). Releasing the strain produces the opposite signal.^[^ ^36^ ^]^ Direct and indirect piezoelectric phenomena are beneficial in bioengineering applications. Specifically, the direct effect is used in wearable devices or energy harvesting devices, whereas the inverse piezoelectric effect is used primarily as an actuator.^[^ ^50^ ^]^ However, recently, it has also been employed in transdermal drug delivery systems.^[^ ^51^ ^]^

The mathematical representations of direct and indirect piezoelectric effects are listed below.

(1) $$D=dT+\varepsilon E\left(Direct\;\;Piezoelectric\;\;effect\right)$$

where D is the electrical displacement, d is the piezoelectric coefficient, T is the stress, and ε is the permittivity of the material.^[^ ^50^ ^]^

(2) $$X=sT+dE\left(Indirect\;\;Piezoelectric\;\;effect\right)$$

Here, X strain, where s is the mechanical compliance, d is the piezoelectric coefficient, and E is the electric field.^[^ ^50^ ^]^

## 4 Piezoelectric Materials

The field of piezoelectric materials has evolved dramatically from the historical discovery of the piezoelectric properties of quartz to today's sophisticated advanced materials.^[^ ^52^ ^]^ Contemporary research on physiological monitoring and implantable devices has established three key material categories: inorganic piezoelectric ceramics, functional synthetic piezoelectric polymers, and natural/biomolecular piezoelectric systems. This strategic classification reflects the progression from robust ceramic materials that provide strong piezoelectric responses to engineered polymers that also enable flexibility and biocompatibility and, finally, to biomolecular systems with great potential for seamless biological integration. This section concisely overviews the unique capabilities/limitations, and progress made in next‐generation piezoelectric materials.

### 4.1 Ceramic Piezoelectric Materials

The evolution of piezoelectric ceramics marks a transformative journey from conventional high‐performance materials to biocompatible, next‐generation solutions for wearable and implantable devices. Ceramics have pioneered the field with superior piezoelectric responses and cost‐effective manufacturing; their integration into biomedical applications has driven an influential shift in material design. Despite their exceptional piezoelectric coefficients, traditional lead‐based ceramics face critical limitations due to toxicity concerns, especially in biomedical implementations.^[^ ^53^ ^]^ Nevertheless, following many endeavors, piezo researchers have successfully developed lead‐free piezo ceramics such as potassium sodium niobate KNN (K_0.5_Na_0.5_NbO_3_) and barium titanate (BT) (BaTiO_3_).^[^ ^54^ ^]^ The field has further evolved with the emergence of advanced functional ceramics, which are explicitly engineered for biointegrated electronics, including biocompatible bismuth sodium titanate (BNT)^[^ ^55^ ^]^ and advanced functional ceramics such as aluminum nitride (AlN) and zinc oxide (ZnO).^[^ ^56^ ^]^ Since these materials were discovered, piezoceramics have been used in many applications, including transducers, sensors, and actuators.^[^ ^54^ ^]^ A summarized overview of ceramic piezoelectric materials is listed in **Table** **1**.

**Table 1:** Overview of Piezoelectric Ceramics.

Material	Structure	d_33_ [pCN^−1^]	Electromechanical Coupling factor (k_33_)	Poling Field [kV mm^−1^]	Dielectric Constant (εr)	Drawbacks	Refs.
PZT	Perovskite	225–600	0.7	2–3	1200–1700	Lead toxicity, brittle	[12, 90, 91, 92, 93]
KNN	Orthorhombic Perovskite	93–700	0.4–0.6	3–4	300–600	Temp. sensitivity, brittle	[93, 94, 95, 96]
BT	Perovskite	191	0.2‐0.3	2–3	1500–2000	Low coupling, high εr	[97, 98, 99]
BNT	Perovskite	75	0.2–0.3	3–5	800–1200	Processing challenges	[76, 100, 101]
AlN	Wurtzite	3‐6	0.12	1–2	8–10	Low piezoelectricity, brittle	[92, 102, 103]
ZnO	Wurtzite Hexagonal	3‐20	0.2	1–2	9–11	Orientation dependent, surface sensitivity	[92, 104]

#### 4.1.1 Conventional High‐Performance Ceramics

##### Lead Zirconate Titanate (PZT)

PZT, discovered in 1950, consists of perovskite structures that exhibit remarkable piezoelectric properties.^[^ ^57^, ^58^ ^]^ An ongoing investigation revealed that the PZT material had a significant electromechanical coupling coefficient of 0.69 and a piezoelectric constant of 360 pCN^−1^. Moreover, the ability of PZT to maintain its piezoelectric capabilities at temperatures exceeding 350 °C renders it a highly desirable material in terms of thermal stability.^[^ ^59^ ^]^ PZT possesses exceptional mechanical durability, outstanding chemical stability, and remarkable resistance to degradation, hence expanding the potential applications of this material. One significant limitation of PZTs is their lack of applicability for flexible applications.^[^ ^41^ ^]^ Due to its elevated Young's modulus of 65 GPa and dense composition, PZT is brittle and cannot withstand substantial deformation. Because of its rigidity, bulk PZT exhibits a high resonance frequency, rendering it unsuitable for harnessing energy from low‐frequency ambient vibrations. Furthermore, the recognized health and environmental hazards make it impractical to employ PZT in specific applications, such as medical implants.^[^ ^60^ ^]^

**Figure 2:**
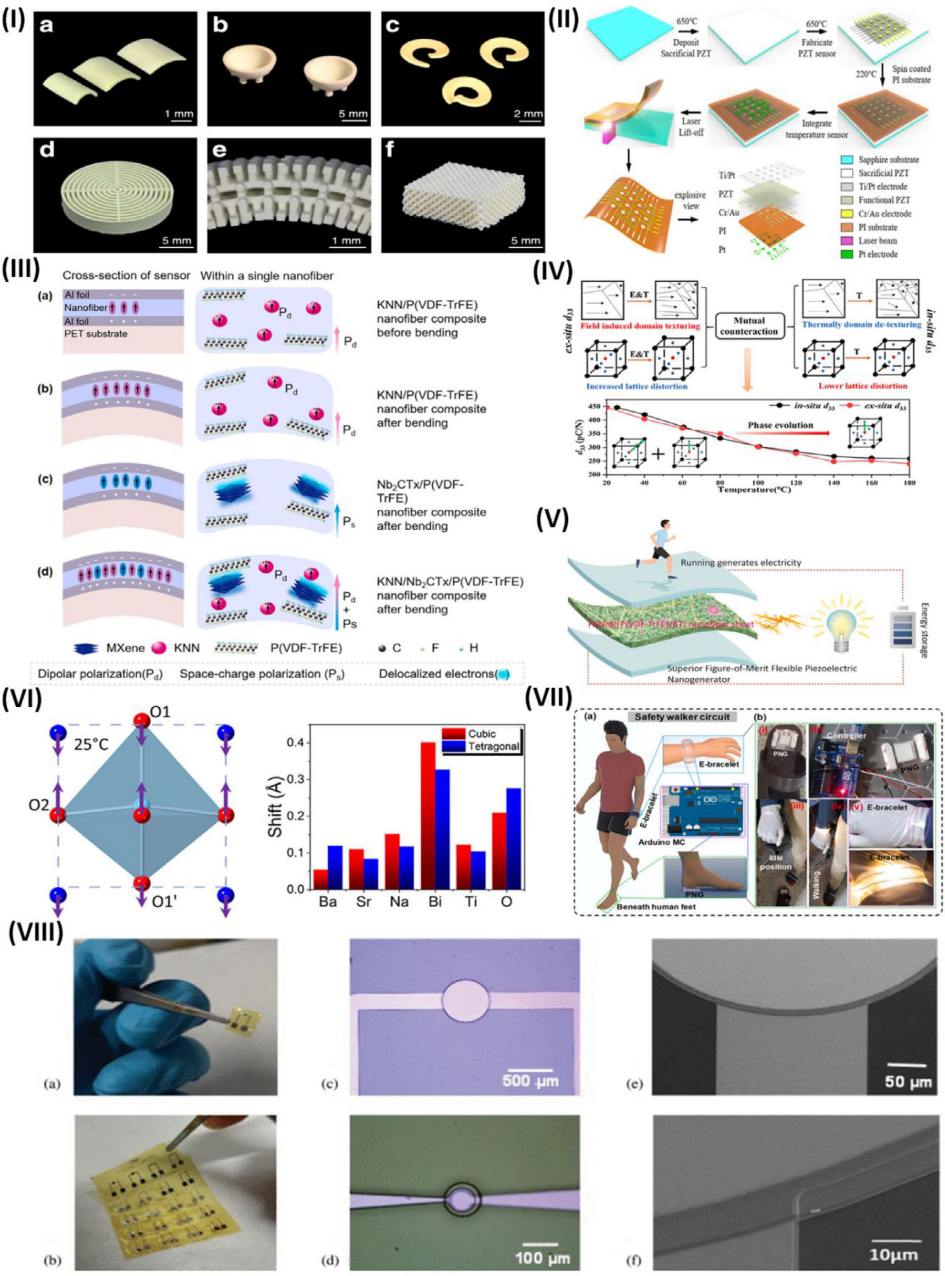
Application and Properties of Piezoceramics for wearable devices. I) 3D printing of PZ with various shapes and curvatures reproduced under a CC‐BY 4.0 license.^[^ ^61^ ^]^ Copyright 2023, Springer Nature. II) Sensor fabrication and cross‐sectional view, reproduced with permission.^[^ ^63^ ^]^ Copyright 2020, American Chemical Society. III) Illustration of the electrostatic effects on the hybrid film structure of P(VDF‐TrFE) nanofibers containing KNN and Nb2CTx, MXene, reproduced with permission.^[^ ^66^ ^]^ Copyright 2024, Elsevier Ltd. IV) KNN‐based ceramic materials with high‐temperature reliability reproduced with permission.^[^ ^69^ ^]^ Copyright 2020, America Chemical Society. V) Schematic illustration of P(VDF‐TrFE)/BaTiO3 coated with a PANI piezoelectric nanogenerator, reproduced with permission ^[^ ^72^ ^]^ Copyright 2023, Elsevier Ltd. VI) Structural arrangement in the composition 0.2(Ba_0.4_Sr_0.6_TiO_3_)‐0.8(Bi_0.5_Na_0.5_TiO_3_) of bismuth sodium titanate (BNT) reproduced under CC‐BY 4.0 license^[^ ^78^ ^]^ Copyright 2024, American Chemical Society. VII) Schematic of the Safety walker circuit reproduced with permission.^[^ ^79^ ^]^ Copyright 2023, Elsevier Ltd. VIII) Visual of piezoelectric sensor with variable diameters, ranging from 10–500 µm and SEM view, reproduced under CC‐BY 4.0 license.^[^ ^80^ ^]^ Copyright 2023, IEEE.

Owing to the fragile characteristics of piezoceramics, current processing techniques for piezoelectric components are limited to producing basic shapes such as flat discs, cylinders, cubes, and rings. Although additive printing has made it possible to create piezoceramics with complex shapes, the resulting transducers have drawbacks such as excessive porosity, weak piezoelectric responses, and limited flexibility in terms of geometry. Lu et al. reported a method for producing piezoelectric microtransducers that are extremely sensitive and function at ultrasonic frequencies. The reported method enables the production of high‐performing piezoelectric transducers with intricate 3D shapes that cannot be achieved via traditional fabrication techniques for piezoceramics, such as hot pressing, molding, sanding, and dicing. These intricate forms provide the possibility of customized transducer applications. **Figure** **2**I(a–f) displays curved components with intentionally engineered curvatures for prospective usage in miniaturized transducers. Additional examples include hemispheric components used in medical imaging, nondestructive testing, and helical components employed to produce acoustic beams with vortex motion for ultrasonic manipulation. This design helps to reduce traverse vibration and improve thickness vibration. Additionally, the system includes architected piezo sensors specifically designed for underwater sensing.^[^ ^61^ ^]^ The traditional clinical environment is well suited for the use of conventional technologies; however, they are not easily adjustable for ongoing employment during ordinary activities. Degderivein et al. developed a PZT‐based conformal device that successfully overcomes these limitations. The combination of small inorganic PZT piezoelectric and semiconductor materials on elastomer substrates enables highly sensitive (<0.005 Pa) and rapid (≈0.1 ms) measurements of pressure on the skin, with minimum hysteresis. The sensor design involves a group of square PZT elements coupled to the gate electrode of an adjacent SiNM n‐MOSFET on a thin silicone elastomer substrate.^[^ ^62^ ^]^ Zhu et al. presented a transfer‐free laser lift‐off method for directly fabricating lead zirconate titanate (PZT) piezoelectric sensors. The technique generally requires, as illustrated in Figure 2II, annealing at 650 °C on flexible substrates such as polyimide (300 °C), polyethene terephthalate (120 °C), and polydimethylsiloxane (150 °C), followed by spin coating. The developed PZT‐integrated bilateral multimodal sensor on a PI substrate demonstrates exceptional stability and performance in detecting distributed dynamic pressure and temperature stimuli, indicating substantial potential for advanced multimodal sensing systems.^[^ ^63^ ^]^

#### 4.1.2 Biocompatible Alternatives

##### Potassium Sodium Niobate (KNN)

Potassium sodium niobate (KNN) is a lead‐free ceramic material that is biocompatible with the biological environment and piezoelectric. While it has lower piezoelectric properties than PZT materials do, recent advancements and various techniques and treatments have made the KNN material equal to PZT with environmentally friendly properties.^[^ ^64^ ^]^ KNN ceramics display strong thermal stability, outstanding mechanical stability, and enhanced chemical properties, making them more suitable for high‐temperature applications. In the field of bioengineering, biocompatibility is always a priority, so the absence of lead in KNN ceramics is a significant benefit over PZT, making it more friendly for wearable applications.^[^ ^65^ ^]^

Deng et al. employed the electrospinning technique to fabricate PENGs of P(VDF‐TrFE) with embedded potassium‒sodium niobate nanoparticles (KNN NPs) codoped with Nb_2_CT_x_. Nb_2_CT_x_ and KNN nanoparticles enhance the piezoelectric performance of a P(VDF‐TrFE) fibrous matrix when subjected to bending stress, as shown in Figure 2III. The β‐phase of the P(VDF‐TrFE) matrix, which contains permanent dipoles and KNN NPs, initially produces a net dipole polarization (Pd), as shown in Figure 2III(a). When an external force is applied to the fiber, it causes the fiber to bend. This bending stretches the dipoles in a direction perpendicular to the curve, increasing the degree of polarization (Figure 2III(b)). The presence of fillers might lead to an increase in the polarization and material dipole moment. The bending of PENG sensors might increase space charge polarization by improving charge transport and accumulation, as shown in Figure 2III(c). The incorporation of Nb_2_CT_x_ and KNN NPs by synergistic integration enhances the piezoelectric response of the composite film, as shown in Figure 2III(d). Furthermore, piezoelectric composite films have shown significant promise in several applications, including monitoring human activities and functioning as advanced alert systems for catastrophic events such as landslides, as evidenced by the test results.^[^ ^66^ ^]^

Among lead‐free piezoelectric materials, KNN‐based ceramics have exceptional thermal stability and strain characteristics.^[^ ^67^ ^]^ These materials are well suited for application in electrical devices as long as the problem of temperature reliability associated with the piezoelectric constant (d_33_) can be resolved appropriately. By examining the influence of external fields on the development of a crystal structure, it has been determined that the electric field does not affect the temperature stability of d_33_.^[^ ^68^ ^]^ This indicates that the variations in d_33_ with temperature are consistent, regardless of whether the observations are conducted at the original location or outside. In contrast, there is a substantial relationship between the electric field and the strain temperature. The temperature stability of d_33_ is affected mainly by the inherent contribution of phase evolution, which is dictated by intrinsic phase coexistence, electric‐field‐driven domain texturing, lattice distortion, and thermally induced domain retexturing. The primary factor influencing temperature stability is the interplay between domain texturing, lattice distortion, and thermal domain retexturing. Xue et al. reported a systematic approach to understanding the temperature stability of d_33_ in KNN‐based ceramics. These findings provide valuable suggestions for the future advancement of materials with high‐temperature reliability, as highlighted in Figure 2IV.^[^ ^69^ ^]^

##### Barium Titanate (BT)

During World War II, scientists synthesized barium titanate BaTiO3 (BT) with a distinctive perovskite structure, which has been extensively used in sonar detection and the use of phonograph needles. It was first recognized for its remarkable capacitance, with a dielectric constant of 1100. However, BT eventually demonstrated piezoelectric characteristics, although it was not electrically polarized. Compared with PZT, pure BT has a relatively low piezoelectric constant of 191 pC N^−1^. Additionally, it has a relatively low Curie temperature of 130 °C and a voltage constant of 11.4 × 10^−3^ V × m N^−1^. Nevertheless, BT ceramics have significantly advanced in attaining piezoelectric qualities equivalent to those of PZT ceramics because of their improved electromechanical capabilities. The piezoelectric characteristics of BT have been enhanced due to the reduction in structure size.^[^ ^70^, ^71^ ^]^

The rapid advancement and integration of the Internet of Things into contemporary life has generated heightened curiosity in self‐sustaining devices and wearable sensors. Mahanty et al. described a straightforward and efficient method for producing nanofiber mats composed of a nanocomposite of polyaniline (PANI)‐coated P(VDF‐TrFE) and BT, as shown in Figure 2V. This process is cost‐effective and straightforward. Introducing PANI‐coated BT NPs into the nanofibers significantly increased the piezoelectric coefficient (d_33_ = 62 pC N^−1^), increasing the output voltage. In addition, the developed flexible devices demonstrated average sensing of mechanical stimuli of 1.2 V kPa^−1^, indicating their potential for extensive use in self‐powered wearable sensors that can detect and record various human movements.^[^ ^72^ ^]^ High‐power piezoelectric applications that operate at the resonance frequency need a high value of d_33_ and a high quality factor (Qm).^[^ ^73^ ^]^ Prior research indicates a negative correlation between d_33_ and Qm, making it challenging to enhance both characteristics concurrently. The addition of manganese (Mn) and zirconium (Zr) to barium titanate (BaTiO_3_) ceramics, known as BT‐xMn‐yZr, improves both the d_33_ piezoelectric coefficient and the Qm quality factor simultaneously, as reported by Yang et al. The experimental findings demonstrate that the defect engineering‐induced domain configuration effect and pinning effect increase the values of d_33_ and Qm in these ceramics. This study demonstrates the methodology for constructing ferroelectric materials free from lead, enhancing their power and performance.^[^ ^74^ ^]^

##### Bismuth Sodium Titanate (BNT)

Bi_0.5_Na_0.5_TiO_3_, often known as BNT, is a lead‐free piezoelectric ceramic with a perovskite structure. Smolensky et al. developed BNT, a lead‐free ceramic, in 1960. It is noted for its excellent piezoelectric properties, with a piezoelectric constant of 75 pCN^−1^. The depolarization temperature of BNT may reach 290 °C. Its strong strain response makes it useful in actuator applications.^[^ ^75^, ^76^ ^]^

In perovskite‐structured systems such as BaTiO3, it is often believed that the ferroelectric transition causes structural changes. This results in polarization due to the displacement of the B‐site cations away from the center.^[^ ^77^ ^]^ Zhang et al. reported that the structural arrangement of 0.2(Ba_0.4_Sr_0.6_TiO_3_)‐0.8(Bi_0.5_Na_0.5_TiO_3_) bismuth sodium titanate (BNT) does not adhere to the suggested model. Researchers are investigating BNT‐based systems as prospective alternatives to the materials currently used in piezo‐/ferroelectric applications to find lead‐free options. Furthermore, the movements of the O_2_^−^ ions and the A‐site cations, particularly Bi^3+^, are quite important, as shown in Figure 2VI. In detail, at room temperature, the tetragonal structure shows a shift in the position of the O1 oxygen atoms, causing the titanium (Ti) atom in the B‐site octahedron to move off‐center along the *c*‐axis. This displacement becomes larger as the temperature increases. However, the position of Ti relative to the equatorial O_2_ atoms remains nearly the same as the temperature changes. Among the A‐site cations, bismuth (Bi) shows much larger displacements than the other cations do in both the tetragonal and cubic phases. Interestingly, most oxygen atoms are more strongly displaced from their ideal positions than most cations are, except for Bi. In the cubic phase, barium (Ba^2^⁺) shows only a small displacement. This detailed study of phase transitions and atomic displacements reveals that certain atoms play a dominant role in creating polarization. These findings challenge traditional theories and open new possibilities for designing advanced materials with improved performance in technologies such as sensors, actuators, and energy storage devices.^[^ ^78^ ^]^ Kurakula et al. developed a piezoelectric nanogenerator (PNG) device that utilizes a flexible composite sheet made of piezoelectric Bi_0.5_Na_0.5_TiO_3_ (BNT) and polyvinylidene fluoride (PVDF) to capture mechanical energy efficiently. The developed PNG device was a real‐time signal generator and could activate the Arduino AMC as a software controller. Figure 2a shows a diagram of the PNG device positioned below the human foot and linked to the AMC to illuminate the LED bracelet. Figure 2VIIb(i) displays the PNG device mounted on the footwear, whereas Figure 2VIIb(ii) illustrates the connection between the AMC and the PNG device. Figure 2VIIb(iii,iv) depicts the device's function during periods of inactivity and movement. Figure 2VIIb(v) displays the E‐bracelet in both the deactivated and activated states.^[^ ^79^ ^]^

##### Aluminum Nitride (AlN)

Aluminum nitride (AlN) is a crystal structure with a wurzite configuration that serves as a lead‐free substitute for PZT. Despite its modest piezoelectric constant of 5 pC N^−1^, AIN has several benefits, such as exceptional thermal and mechanical stability. AIN has a Curie temperature that may surpass 1000 °C while maintaining stability in moisture and displaying high compatibility with analogous metal oxide semiconductor techniques. AIN thin films are well suited for resonance devices and sensors because of their exceptional thermal conductivity, minimal dielectric losses, and low acoustic losses.^[^ ^56^ ^]^

Piezoelectric microelectromechanical systems (MEMSs) meet the growing need for low‐power, small sensors. Tactile applications and pressure readings are employed in robotics and healthcare. This setting requires flexible technologies because of their high sensitivity and adaptability to the studied surface. Carluccio et al. explained the use of compact, flexible piezoelectric devices to integrate sensors that can detect and identify localized pressures and contacts per unit area while reducing interference. To accomplish this goal, piezoelectric sensors made of aluminum nitride (AlN) with sizes ranging from 5 to 500 µm were developed, as highlighted in Figure 2VIII. A range of piezoelectric sensors with variable diameters, ranging from 5 to 500 µm, have been developed, as shown in Figure 2VIII(a,b). Optical microscope images of samples with diameters of 500 µm and 50 µm are presented in Figure 2VIII(c,d), respectively. The preview in Figure 2VIII(e,f) displays scanning electron microscope (SEM) images of a piezoelectric tactile sensor. The enhancements in sensitivity and range of variation indicate the potential use of this integrated technique for directly calibrating piezoelectric sensors and for monitoring the electrical signal during precise and delicate motions associated with grasping and manipulation tasks.^[^ ^80^ ^]^

##### Zinc Oxide (ZnO)

ZnO nanomaterials (nanoparticles, rods, flowers, and fibers) demonstrate flexibility and sensitivity in physiological monitoring. The flexibility of ZnO nanostructures is evidenced by their incorporation into wearable and implantable sensors. Jiang et al.^[^ ^81^ ^]^ demonstrated a wireless implantable strain sensing application using a highly stretchable ZnO/poly(dimethyl acrylamide) nanocomposite hydrogel, which allows monitoring of biological tissues under significant mechanical deformation (up to 200%). This flexibility is crucial for physiological monitoring systems that conform to the dynamic nature of human tissues. Moreover, the sensitivity of ZnO nanostructures has been highlighted in various biosensor applications. Chen et al.^[^ ^82^ ^]^ reported that a carbon nanotube/PVA/nano‐ZnO composite exhibited excellent piezoresistive and piezoelectric sensing characteristics, making it feasible for stress and strain sensing. This high sensitivity is essential for accurately detecting physiological changes. Additionally, Morais et al.^[^ ^83^ ^]^ demonstrated that ZnO nanorods could be used for nonenzymatic glucose detection, showing a high correlation with clinical data, thus validating their effectiveness in monitoring glucose levels in human plasma.

Integrating ZnO nanostructures into flexible and self‐powered systems further enhances their applicability in physiological monitoring; for example, Mao et al.^[^ ^84^ ^]^ developed self‐powered piezoelectric‐biosensing textiles that utilize tetrapod‐shaped ZnO nanowires for physiological monitoring during sports activities. This innovation highlights the flexibility and ability of ZnO nanostructures to operate without external power sources, which is a significant advantage in wearable technology. Furthermore, the versatility of ZnO nanostructures extends to their use in various sensing modalities, including electrochemical and optical sensors. Napi et al.^[^ ^85^ ^]^ emphasized that ZnO nanostructures are active sites in electrochemical biosensors, significantly influencing their sensitivity and stability. Additionally, incorporating ZnO nanowires in fiber‒optic sensors has increased sensitivity through localized surface plasmon resonance effects.

Recent studies have emphasized the versatility and effectiveness of ZnO in enhancing the performance of wearable and implantable sensors. For example, Arrabito et al.^[^ ^86^ ^]^ reported ZnO nanostructured films in self‐cleaning bending sensors, noting their excellent biodegradability and biocompatibility, as recognized by regulatory bodies such as the FDA. This highlights the suitability of ZnO for wearable sensor applications that require both functionality and safety in contact with human skin. Moreover, the integration of ZnO in piezoelectric sensors has significantly enhanced their performance. Chung et al.^[^ ^87^ ^]^ demonstrated that the attachment of ZnO nanowires to poly(vinylidene fluoride) (PVDF) films improved the piezoelectric properties of the material, making it ideal for impact force sensing in wearable applications.

This advancement indicates a trend toward the use of ZnO to improve the sensitivity and accuracy of wearable devices, which is crucial for effective physiological monitoring. Additionally, the development of self‐powered wearable sensors incorporating ZnO has gained traction. Hu et al.^[^ ^88^ ^]^ introduced a self‐powered sound‐driven humidity sensor that utilizes ZnO nanoarrays, demonstrating its potential for continuous health monitoring without external power sources. This innovation aligns with the current trend of creating more efficient and sustainable wearable technologies. Furthermore, the use of ZnO in transparent and flexible electronics is being explored, as noted by Gherendi et al.,^[^ ^89^ ^]^ who discussed the fabrication of ZnO thin film transistors for low‐power disposable electronics, including wearable sensors. The emphasis on flexibility and transparency is particularly relevant for wearable devices, which must be comfortable and unobtrusive for users. The increasing integration of ZnO in wearable sensors is also reflected in the broader context of advancements in sensor technology.

### 4.2 Piezoelectric Polymers

In the 19^th^ century, piezoelectricity was not explored in polymers. In the late 1960s, Kawai et al. discovered that polymers may exhibit significant piezoelectric properties that can revolutionize the world through their inherent flexibility.^[^ ^35^, ^105^ ^]^ There are different types of piezoelectric polymers, such as high‐performance fluoropolymers, such as polyvinylidene fluoride (PVDF), and their copolymers, which are tiny ferroelectric crystals dispersed within an amorphous matrix. When these polymers undergo poling, their dipoles undergo reorientation and alignment, resulting in the material acquiring robust piezoelectric properties.^[^ ^106^ ^]^ Conventional piezoelectric polymers, such as polyamides^[^ ^107^ ^]^ and polyacrylonitrile (PAN),^[^ ^108^ ^]^ are engineered as high‐performance fluoropolymers to increase their piezoelectric performance. Organic piezoelectric polymers contain functional polar groups, such as cyano groups or carboxylic groups, which form crystalline structures and molecular polar dipoles. In the application of mechanical deformation, the dipole orientation and molecular polar dipoles generate piezoelectricity.^[^ ^109^ ^]^ The mechanism of action of biodegradable piezopolymers is similar to that of organic piezopolymers, but they are designed to break down within a specific time period. Biodegradable piezopolymers offer eco‐friendly benefits by retaining the piezoelectric characteristics, which are generated via mechanical deformation of the polar dipoles to produce electricity.^[^ ^110^ ^]^ Biopolymers, which are derived from natural materials such as chitosan and cellulose, can also exhibit piezoelectric properties due to their inherent molecular structure and organization. Their piezoelectric response is often influenced by factors such as moisture content and the arrangement of molecular chains, which shift under stress to generate charges.^[^ ^111^ ^]^ Therefore, this section discusses the current advancements in high‐performance fluoro‐piezoelectric polymers, conventional piezoelectric polymers, biodegradable piezoelectric polymers, and biopiezopolymers. **Tables** **2** and **3** summarize the overall overview of piezopolymers and biopiezoelectric materials.

**Table 2:** Summary of conventional piezoelectric polymers.

Polymer	Type	d₃₃ (pCN^−1^)	k (Coupling factor)	Poling Field (kV/mm)	Dielectric Constant (ε_r_)	Applications	Limitations	References
PVDF	Semicrystalline	−20 to −33	0.1–0.2	0.6–1.2	≈12–13	Sensors, actuators	Low thermal stability	[93, 145, 146]
P(VDF‐TrFE)	Semicrystalline	21.5–74	0.12–0.18	0.8–1.5	8–15	Thermal/mechanical sensors	Higher cost	[93, 147, 148, 149]
Polyureas	Amorphous	≈5	≈0.1	≈1	4–6	Wearables, sustainable	Limited flexibility	[150, 151, 152]
Polyamides (Nylon‐11)	Semicrystalline	3.8–4	≈0.05	1–2	3–5	PENGs, self‐powered devices	Complex processing	[93, 153, 154]
PAN	Semicrystalline	5–10	≈0.05	1–2	5–7	High‐temperature sensors	Difficult poling	[108, 155, 156]
PVC	Amorphous	0.5–5	≈0.05	1–2	3–5	Sensors, nanogenerators	Environmental concerns	[140, 157]

**Table 3:** Overview of Biopiezoelectric Materials.

Material	Piezoelectric Constant	Poling Field (kV mm^−1^)	Dielectric Constant (ε_r_)	Key Features	Applications	References
Poly‐L‐lactic acid (PLLA)	7–16 pCN^−1^	≈0.1–0.3	≈3–5	Biodegradable; enhanced piezoelectricity by stretching/electrospinning	Biodegradable sensors, implants, energy harvesters	[174, 188, 189]
Polyhydroxybutyrate (PHB)	1–2 pCN^−1^	Not typically polled	≈3–5	Biocompatible; piezoelectricity induced by mechanical stretching	Medical implants, tissue engineering	[110, 190, 191]
Cellulose Nanocrystals (CNCs)	≈210 pm V^−1^ (shear mode)	–	≈6–10	High shear piezoelectricity due to crystalline alignment	Flexible electronics, biosensors	[192, 193, 194]
Diphenylalanine (FF) Nanotubes	9.9–17.9 pm V^−1^	–	–	Self‐assembled peptide nanotubes with significant piezoelectricity	Nanogenerators, biosensors	[109, 195, 196]
β‐Glycine	≈178 pm V^−1^	–	–	High piezoelectric voltage constants among amino acids	Bioelectronics, sensors	[109, 197]
Collagen	1–2 pCN‐1	–	≈12–15	Natural protein; piezoelectricity due to triple‐helix structure	Tissue engineering, biosensors	[109, 198]
Silk Fibroin	≈1.5 pCN‐1	–	≈10–12	Piezoelectricity influenced by β‐sheet content and orientation	Flexible electronics, biomedical devices	[199, 200, 201]
Hydroxyapatite	Flexoelectric effect (no intrinsic d₃₃)	–	≈10–20	Generates electric charge due to strain gradient (flexoelectricity)	Bone tissue engineering, bioelectronic interfaces	[202]

#### 4.2.1 High‐Performance Fluoropolymers

##### Polyvinylidene Fluoride (PVDF)

The exploration of the electrical capabilities of PVDF started in 1969 when Kawai demonstrated that thin films subjected to poling had a very high piezoelectric coefficient of 6–7 pCN^−1^.^[^ ^112^ ^]^ This value was almost ten times greater than that previously reported for any other polymer. The hydrogen and fluorine atoms are arranged symmetrically throughout the polymer chain, resulting in distinct polarity effects that impact the electromechanical response, solubility, dielectric characteristics, and crystal shape and provide a very high dielectric constant.^[^ ^113^ ^]^ PVDF has a dielectric constant of approximately 12, which is four times greater than that of typical polymers.^[^ ^106^ ^]^ This characteristic makes PVDF appealing for device incorporation since higher dielectric materials tend to have a lower signal‐to‐noise ratio. The PVDF material exhibited an amorphous phase with a glass transition temperature much lower than the ambient temperature (−35 °C). As a result, the material is very flexible and easily deformed at room temperature.^[^ ^114^ ^]^ Polyvinylidene fluoride (PVDF) usually has a crystallinity ranging from 50% to 60%, which is influenced by its temperature and processing background. PVDF has at least four crystal phases (alpha, beta, gamma, and z), with at least three having polarity. The most stable, nonpolar alpha phase is obtained when PVDF is solidified from its molten state. This phase may be converted into the polar beta by physically stretching it at high temperatures or twisting the molecular chain axis with a strong electric field of ≈130 MV m^−1^.^[^ ^115^, ^116^ ^]^

**Figure 3:**
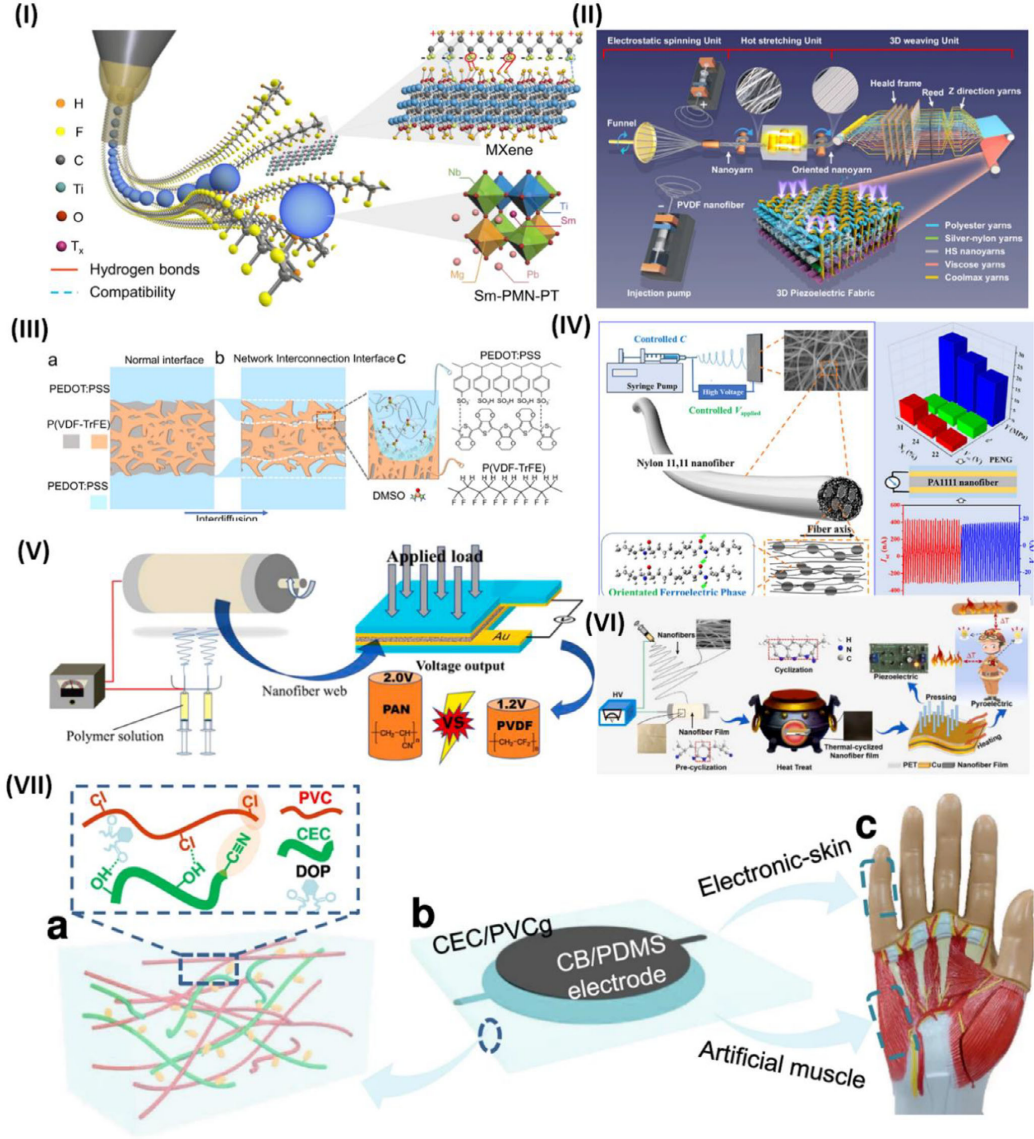
Piezoelectric studies on polymers and their application in wearable sensors. I) Graphical illustration of electrospun nanofibers and interactions of MXenes with PVDF‐doped Pb(Mg1/3Nb2/3)O3‐PbTiO, reproduced under a CC‐BY 4.0 license.^[^ ^118^ ^]^ Copyright 2022, Springer Nature. II) Schematic illustration of the 3DPF strain sensor and its fabrication and thermal processing steps, which were reproduced under a CC‐BY 4.0 license.^[^ ^119^ ^]^ Copyright 2024, Springer Nature. III) Interface engineering of P(VDF‐TrFE) with a layer of PEDOT:PSS for pressure and thermal sensing, reproduced under a CC‐BY 4.0 license.^[^ ^125^ ^]^ Copyright 2023, Springer Nature. IV) Electrospun nylon 11 fibers with an oriented ferroelectric phase and performance overview, reproduced with permission. ^[^ ^134^ ^]^ Copyright 2024, American Chemical Society. V) Schematic illustration of electrospinning of PAN and the output voltage of PAN versus PVDF, reproduced with permission.^[^ ^138^ ^]^ Copyright 2018 Elsevier Ltd. VI) Electrospinning of piezoelectric and pyroelectric PAN/Zn (Ac)_2_ hybrid sensors for high‐temperature applications, reproducedwith permission.^[^ ^139^ ^]^ Copyright 2024, Elsevier. VII) Schematic illustration of a CEC/PVCg matrix for actuating and sensing applications reproduced under a CC‐BY 4.0 license.^[^ ^144^ ^]^ Copyright 2023, Springer Nature.

The mechanical flexibility and piezoelectric properties of polymer‐ceramic piezoelectric composites surpass those of various piezopolymers. However, these materials have low inherent polarization and usually have poor crystallinity, limiting their piezoelectric capacity.^[^ ^117^ ^]^ Ti_3_C_2_Tx MXene anchoring is a technique devised by Su et al.^[^ ^118^ ^]^ to manipulate the intermolecular interactions inside a polymer matrix in an all‐trans conformation. The phase field simulations and molecular dynamics confirmed that OH bonds on Ti_3_C_2_T_x_ enhance the composite's polarization and dipole alignment. This strategy was experimentally proven by electrospinning the MXene with PVDF‐doped Pb (Mg1/3Nb2/3)O3‐PbTiO3, as highlighted in **Figure** **3**I.^[^ ^118^ ^]^ Fan et al.^[^ ^119^ ^]^ developed PVDF piezoelectric nanoyarns with a remarkable strength of 313.3 MPa. These nanoyarns are woven with other yarns to create a 3DPF sensor utilizing contemporary 3D textile technologies, as illustrated in Figure 3II. The 3DPF material has the highest tensile strength of all flexible piezoelectric sensors at 46.0 MPa. The 3DPF uses antigravity unidirectional liquid transmission to move sweat from the inner layer near the skin to the outer layer in 4 seconds. Additionally, the 3DPF is as durable and comfortable as cotton T‐shirts. This investigation describes advanced, comfortable, flexible wearable electronics.^[^ ^119^ ^]^

##### Poly(Vinylidene Fluoride‐Co‐Trifluoroethylene) (P(VDF‐TrFE)

Polyvinylidene fluoride copolymers containing trifluoroethylene (TrFE) and tetrafluoroethylene (TFE) have significant piezoelectric, pyroelectric, and ferroelectric properties.^[^ ^120^ ^]^ In this context, these polymers are being addressed together since they exhibit comparable behavior when copolymerized with PVDF. A notable morphological characteristic of the comonomers is their ability to induce the polymer to adopt an all‐trans conformation, creating a polar crystalline phase. This avoids the need for mechanical stretching to achieve a polar phase.^[^ ^121^ ^]^ Poly(vinylidene fluoride‒trifluoroethylene) (P(VDF‒TrFE)) has a much greater degree of crystallization than does polyvinylidene fluoride (PVDF), with crystallinity levels reaching up to 90%, which results in stronger remanent polarization, a lower coercive field, and more distinct hysteresis loops. TrFE additionally increases the maximum operating temperature by ≈20 °C, reaching nearly 100 °C.^[^ ^122^ ^]^

In contrast, copolymers containing TFE have shown a reduced level of crystallinity and a decreased melting temperature compared with those of the PVDF homopolymer.^[^ ^123^ ^]^ While the piezoelectric constants of the copolymers are not as significant as those of the homopolymer, the favorable characteristics of P(VDF‐TrFE), such as its processability, improved crystallinity, and higher operating temperature, make it advantageous for many applications.^[^ ^124^ ^]^ Ferroelectric polymers have potential in mechanical and thermal sensing applications, yet their sensitivity and detection limits are still limited. By cross‐linking a thin film of a ferroelectric poly(vinylidene fluoride‒cotrifluoroethylene) copolymer (P(VDF‒TrFE)) with a layer of PEDOT:PSS, interface engineering might enhance charge collection, as reported by Li et al. and illustrated in Figure 3III. The device's sensitivity is 2.2 V kPa^−1^ within the pressure range of 0.025–100 kPa and 6.4 V K^−1^ within the temperature range of 0.05–10 K. This study explores the manipulation of electrode contact to increase the sensitivity of a ferroelectric polymer sensor.^[^ ^125^ ^]^

#### 4.2.2 Conventional Piezoelectric Polymers

##### Polyureas

Takahashi examined the dielectric and pyroelectric properties of polyurea sheets, revealing their piezoelectric characteristics. Since the early 1990s, several heterocyclic and aromatic polyureas have been developed and shown to possess piezoelectric properties. Polyureas with a powerful odor were the first to exhibit piezoelectric characteristics.^[^ ^126^ ^]^ The piezoelectric constant, which measures the ability of a material to generate an electric charge in response to applied mechanical stress, remains constant at temperatures up to 200 °C. This characteristic ensures that the material maintains stability even when exposed to high temperatures. Owing to their decreased dielectric loss compared with those of other polymers, they exhibit high pyroelectric coefficients. The d constant at room temperature is ≈5 pCN^−1^, increasing as the temperature increases.^[^ ^127^, ^128^ ^]^ Piezoelectric polymers derived from sustainable sources are crucial for achieving carbon neutrality and reducing the dependence on fossil fuels. These polymers can be used in wearable electronic devices, which increases their importance. Gu et al. generated nanofiber films by electrospinning PLLA utilizing CO_2_‐based polyurea (PU) derived from CO_2_ and diamine. The PU/PLLA piezoelectric sensor produces 19 V at a concentration of 16 wt% PU, which is 15 times higher than the output of the PLLA sensor. Furthermore, the PU/PLLA sensor exhibited excellent stability and durability. This investigation describes the first composite piezoelectric materials synthesized using carbon dioxide and poly(l‐lactic acid).^[^ ^129^ ^]^

##### Polyamides

Kawai reported the first detection of a minimal amount of piezoelectricity in polyamides, also known as nylons. Scheinbeim and Newman began a detailed analysis of odd‐numbered nylons in 1980, which resulted in almost two decades of further research into their piezoelectric and ferroelectric properties.^[^ ^130^ ^]^ Odd nylon monomers have an even number of methylene groups and one amide group. The dipole moment is 3.7 Debye (D). Polyamides crystallize in all‐trans conformations and are organized such that neighboring amine and carbonyl groups may make most hydrogen bonds possible. The amide dipoles in the monomer align in an odd number, yielding a net dipole moment.^[^ ^131^ ^]^ At ambient temperature, nylons with odd numbers have lower piezoelectric constants than does PVDF. Nonetheless, when evaluated beyond their glass transition temperature, they show identical ferroelectric and piezoelectric capabilities and much increased thermal stability. The piezoelectric d and e constants significantly and quickly increase with increasing temperature.^[^ ^131^ ^]^

Tu et al. reported a polyamide‐imide (PAI) matrix doped with a lead titanate nanowire (PbTiO_3_ NW) piezoelectric nanocomposite, which shows increased piezoelectricity at increased temperatures. The piezoelectric g_31_ and d_31_ coefficients of the nanocomposite films with different weight percentages of PbTiO_3_ NWs were studied in situ at temperatures ranging from 25 to 250 °C. This investigation comprehensively explains how temperature and filler concentration affect piezoelectric properties.^[^ ^132^ ^]^ Piezoelectric nanogenerators, or PENGs, constitute a highly promising method for gathering and using mechanical energy from the environment. The use of piezoelectric polymers in creating PENGs provides a significant advantage in wearable devices with self‐powered systems. However, there is room for improvement in performance since only a few piezoelectric polymers have been explored for PENGs.^[^ ^133^ ^]^ Yang et al. developed a PENG employing a unique piezoelectric nylon known as odd‒odd nylon (nylon 11,11). The electrospinning parameters were investigated to obtain homogenous nylon 11,11 nanofibers with excellent piezoelectric crystalline states. The quantity of material in the electrospinning fluid affects the shape and arrangement of the piezoelectric‐active crystal. In ferroelectric crystals, the domain orientation is determined by the applied electric field, as shown in Figure 3IV. Nylon 11,11 nanofiber piezoelectric nanogenerators (PENGs) show great potential as electromechanical response systems and self‐powered devices.^[^ ^134^ ^]^

##### Polyacrylonitrile (PAN)

Polyacrylonitrile (PAN) is a synthetic, partially crystalline organic polymer resin characterized by a linear chemical formula (CH_2_CHCN)n. Most PAN resins are copolymers, with acrylonitrile constituting the primary monomer.^[^ ^135^ ^]^ The substantial presence of a nitrile dipole in PAN suggests that an externally supplied electric field may orient it. Nevertheless, PAN poses unique complications that are absent in other polymers containing nitrile groups.^[^ ^136^ ^]^ Multiple investigations have shown that the challenge of poling PAN under unstretched conditions is attributed to the strong dipole‒dipole interactions among nitrile groups within the same molecule. This interaction fosters avoidance between them, hindering the establishment of strong polarity. When a material undergoes stretching, intermolecular dipole interactions facilitate the aggregation of individual chains, leading to the creation of structured regions.^[^ ^135^, ^137^ ^]^ Polyacrylonitrile (PAN), a polymer without a specific structure, is recognized for its considerably reduced piezoelectricity compared with that of PVDF. Moreover, Wang et al. highlighted the exceptional piezoelectric property of electrospun PAN nanofiber membranes. The compression of a 5 cm^2^ PAN nanofiber nonwoven membrane segment may produce a voltage of up to 2.0 V. Under the same conditions, this membrane results in a greater electrical output than do the other PVDF nanofiber membranes, as shown in Figure 3V. The presence of several planar Sawtooth PAN configurations inside the nanofibers was shown to be the cause of the piezoelectric characteristics. These unexpected findings might inspire the development of innovative piezoelectric materials and technologies.^[^ ^138^ ^]^ Yin et al. reported an electrospinning PAN/Zn(Ac)_2_ piezoelectric sensor, which can work at temperatures greater than 500 °C, as illustrated in Figure 3VI. The reported sensor demonstrated 14.13 V at RTP and 14.86 V at high temperature with durability over 10 000 cycles for 60 days at 400 °C. This sensor is the best candidate for high‐temperature applications.^[^ ^139^ ^]^

##### Polyvinyl Chloride (PVC)

Polyvinyl chloride (PVC) has a carbon‒chlorine dipole, which may be prolonged to provide a modest level of piezoelectricity. The enduring and reproducible piezoelectric and pyroelectric properties of PVC were validated.^[^ ^140^, ^141^ ^]^ Broadhursts began their research using PVC to explore and understand the piezoelectricity of amorphous polymers. Empirical evidence suggests that PVC has piezoelectric coefficients d_31_ within the 0.5% to 1.5 pCN^−1^ range. The results were significantly improved by simultaneously elongating and corona‐poling the film. The enhanced piezoelectric coefficient d_31_ varies from 1.5 to 5.0 pCN.^−1[^ ^128^ ^].^ Fu et al. used the solution casting technique on polyvinyl chloride, which was plasticized with diethyl adipate (PVC/DEA). The PVC/DEA film was subjected to thermal poling, and subsequently, an analysis was carried out to determine the piezoelectric coefficient (d_33_). The results revealed that the d_33_ value of the PVC/DEA film first increased but then decreased with increasing DEA concentration. This work introduces a novel method for developing piezoelectric materials with excellent performance, which may be used as sensors or nanogenerators.^[^ ^140^ ^]^ Polyvinylidene fluoride (PVDF) and polyvinyl chloride (PVC) are significant polymers in electronics and optoelectronic devices because of their exceptional transparency and ability to create thin films. Owing to these advantages, several research teams are investigating the chemistry of PVDF/PVC to increase its pyroelectric coefficient. The introduction of nanofillers may enhance the pyroelectric characteristics of PVDF/PVC. The incorporation of PVC into PVDF may increase the reliability and robustness of composite thin films.^[^ ^142^ ^]^

Dielectric elastomers (DEs) are widely used in soft actuation and sensing. Current DE actuators require powerful driving electrical fields because of their low permittivity. Most DE actuators and sensors have viscoelastic effects, causing mechanical loss and signal shifts. To solve this issue, Huang et al. investigated how to make high‐permittivity, low‐viscoelasticity polyvinyl chloride (PVC) elastomers. Plasticized PVC gel (PVCg) with cyanoethyl cellulose (CEC) has two significant effects. First, the CEC increases the dielectric permittivity to 18.9 at 1 kHz. Second, it considerably minimizes PVCg's viscoelastic effects, as indicated by a 0.04 mechanical loss at 1 Hz. The CEC/PVCg sensors can detect human motions accurately because of their high sensitivity, fast response, and minimal signal drift, as shown in Figure 3VII.^[^ ^144^ ^]^

#### 4.2.3 Natural and Biomolecular Piezoelectric Materials

##### Amino Acid‐ and Peptide‐Based Piezoelectric Systems

Amino acids are chemical compounds with a carboxyl group, a radical group, and an amino group. Peptides are formed from long chains of amino acids, and proteins are aggregates of polypeptides.^[^ ^158^ ^]^ Vasilescu first identified the piezoelectric capabilities of amino acids attributable to their radical chemical structure. Numerous amino acids have piezoelectric properties; nevertheless, maintaining the biocompatibility, flexibility, and piezoelectricity of amino acids, peptides, and proteins in biomedical applications is challenging.^[^ ^34^, ^159^ ^]^ The electronic and supramolecular configurations of biomolecular assemblies are significantly influenced by alterations in their molecular architecture, resulting in a markedly altered piezoelectric response. A comprehensive understanding of the relationships among the chemistry of molecular building components, crystal packing, and quantitative electromechanical response remains insufficient.^[^ ^160^ ^]^

**Figure 4:**
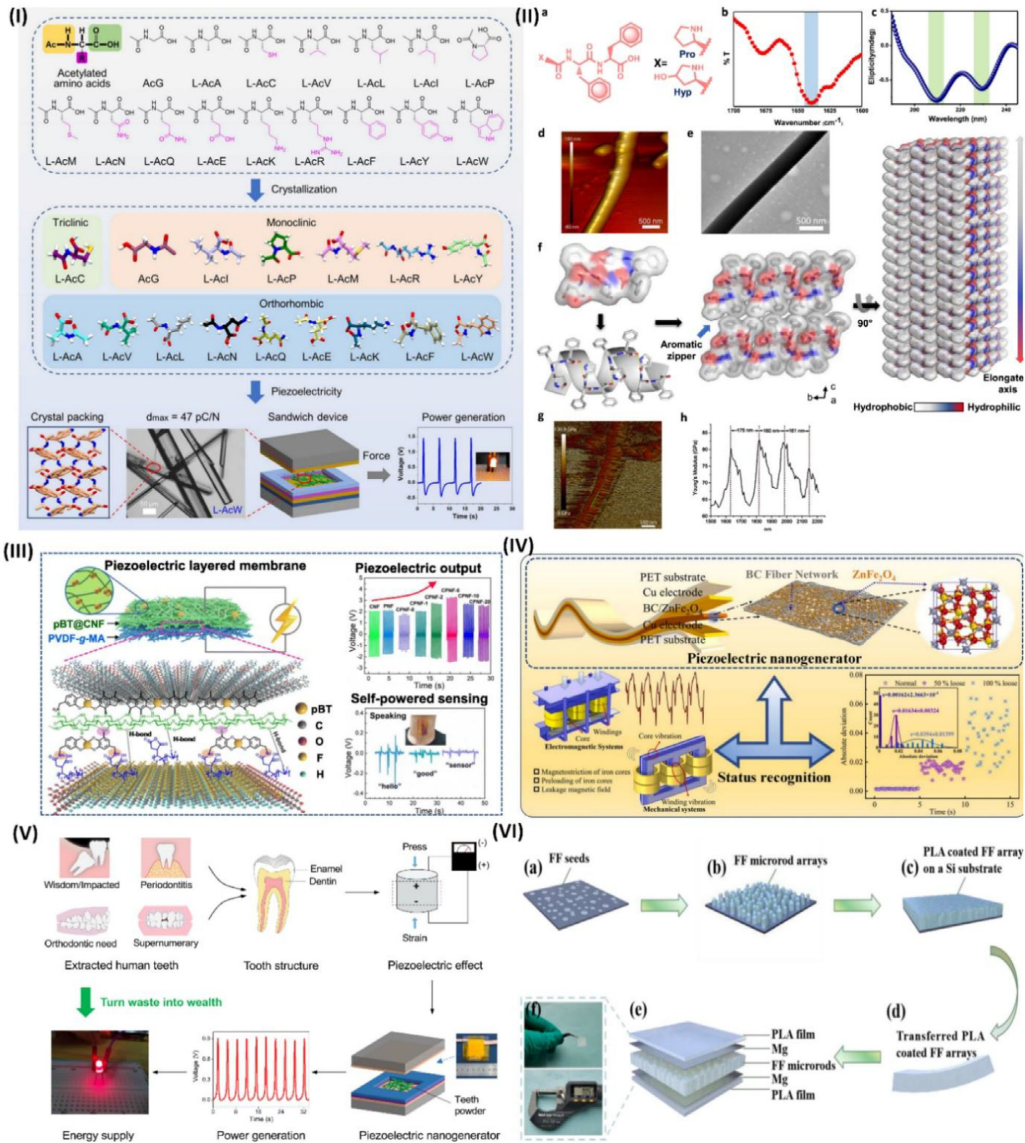
Piezoelectric application of biological and degradable piezoelectric materials. I) Visual representations of N‐acetylated amino acid architectures with diverse side chains that crystallize in unique space groups. Various forms of supramolecular packing influence the piezoelectric properties of crystals. A nanogenerator capable of generating power and illuminating LEDs is constructed utilizing L‐AcW crystals with significant piezoelectric properties. Reproduced with permission.^[^ ^161^ ^]^Copyright 2023, American Chemical Society. II)Structural and chemical characterization of Hyp‐Phe‐Phe. a) Chemical structure of Pro‐Phe‐Phe and self‐assembling tripeptides. b,c) Fourier transform infrared (FTIR) and circular dichroism (CD) spectroscopy of Hyp‐Phe‐Phe. d) AFM image of the tripeptide fibrillar. e) TEM image of the tripeptide fibers. f) Single‐crystal structure of Hyp‐Phe‐Phe. g,h) Quantitative nanomechanical mapping via atomic force microscopy (QNM‐AFM). Reproduced under CC with a 4.0 license.^[^ ^162^ ^]^ Copyright 2021, Springer Nature. III) A flexible PVDF‐cellulose‐based piezoelectric membrane for self‐power sensing and energy harvesting was reproduced with permission.^[^ ^166^ ^]^ Copyright 2021 Elsevier Ltd. IV) Schematic illustration of piezoelectric nanogenerator composites with their application in status recognition, reproduced with permission.^[^ ^167^ ^]^ Copyright 2024, Elsevier Ltd. V) Schematic illustration of recycled human teeth in a piezoelectric nanogenerator, starting with the piezoelectric action of the teeth and concluding with energy delivery, reproduced with permission.^[^ ^171^ ^]^ Copyright 2024, Elsevier. VI) The development procedure of diphenylalanine‐based degradable piezoelectric nanogenerators was reproduced with permission.^[^ ^180^ ^]^ Copyright 2021, Elsevier.

Wang et al.^[^ ^161^ ^]^ conducted a thorough investigation of the possibility of supramolecular engineering to augment the piezoelectric properties of amino acid‐based assemblies. These findings indicate that the piezoelectric sensitivity of acetylated amino acids may be significantly enhanced by altering their side chains, hence augmenting the polarization of their supramolecular structures. Unlike most naturally occurring amino acid assemblies, chemical modification by acetylation also increased the maximum piezoelectric tensors. This work illustrates the construction of high‐performance functional biomaterials from fundamental, easily modifiable components, using supramolecular engineering to systematically adjust the piezoelectric response in amino acid‐based assemblies, as highlighted in **Figure** **4**I. The electromechanical properties of hierarchically structured natural materials, such as collagen, may be enhanced by molecular engineering to provide technologically beneficial piezoelectricity.

Bera et al.^[^ ^162^ ^]^ created a peptide‐based piezoelectric generator using a key collagen building block and an innovative helical configuration comprising Phe‐Phe‐derived peptides, Pro‐Phe‐Phe, and Hyp‐Phe‐Phe, made only from proteinogenic amino acids. The incorporation of a hydroxyl group is anticipated to increase the piezoelectric responsiveness by an order of magnitude (d_35_ = 27 pm V^−1^). This study illustrates piezoelectricity's computationally directed molecular engineering in peptide nanotechnology, resulting in a potential device. Furthermore, Figure 4II illustrates the structural and chemical characterization of Hyp‐Phe‐Phe, with Figure 4II(a) emphasizing the chemical structure of Pro‐Phe‐Phe and self‐assembling tripeptides. Figure 4II(b) illustrates the use of Fourier transform infrared (FTIR) and circular dichroism (CD) spectroscopic methods to characterize the secondary structure of Hyp‐Phe‐Phe in solution. The CD spectra robustly corroborated the FTIR findings, displaying twin negative peaks indicative of helical conformations, as shown in Figure 4II(c). Figure 4II(d,e) demonstrated that the tripeptide self‐assembled into homogeneous high aspect ratio fibers with a diameter of 500 nm, extending for many micrometers. Single‐crystal X‐ray diffraction analyses elucidated the advantageous molecular interactions in Figure 4II(f) that govern the supramolecular arrangement. Figure 4II(g,h) presents quantitative nanomechanical mapping via atomic force microscopy (QNM‐AFM).^[^ ^162^ ^]^

##### Piezoelectricity in Polysaccharides

Polysaccharides are widely distributed macromolecules of many monosaccharide units linked together by glycosidic bonds and are present in animals, plants, and microorganisms. They have been employed for transitory medical purposes owing to their intrinsic enzymatic degradability.^[^ ^163^ ^]^ Natural polysaccharide materials such as cellulose and chitin have a low‐symmetry hierarchical fibrous structure with strong piezoelectric characteristics. Polysaccharides have recently emerged as feasible alternatives for the development of environmentally friendly products. Polysaccharide‐based polymers have been studied for use in the food, packaging, and healthcare industries. Numerous studies have shown that processing techniques and treatments may affect the inherent properties of polysaccharides, making them suitable for energy applications.^[^ ^164^ ^]^ Cellulose‐based piezoelectric energy harvesting is becoming more prevalent. However, the low piezoelectricity of natural cellulose is a significant challenge.^[^ ^165^ ^]^ Wang et al. developed piezoelectric nanogenerators with strong, long‐lasting multilayer membranes made of cotton cellulose interfaced with maleic anhydride‐grafted polyvinylidene fluoride (PVDF‐g‐MA) nanofibers. Polydopamine@BaTiO3 (pBT) nanoparticles act as interlayer bridges, creating simple and scalable interlocking layer‒layer interfaces that covalently bond component layers. Upscaled cellulose‐based membranes might be utilized to make energy harvesters that are ecologically friendly, flexible, and long‐lasting, as well as self‐powered wearable sensors, as illustrated in Figure 4III.^[^ ^166^ ^]^ Wang et al. used coprecipitation to create an outstandingly adaptable piezoelectric composite made of zinc ferrite and bacterial cellulose. The results show that the interfacial and piezoelectric constants of the composites increase with increasing percentage of zinc ferrite (ZnFe_2_O_4_). The piezo output response increases and decreases, with a sensitivity of 80 mV N^−1^. This work enhances the development and use of smart piezoelectric sensors for monitoring power equipment, as highlighted in Figure 4IV.^[^ ^167^ ^]^

##### Piezoelectricity in the Human Body

The human body consists of complex biological structures composed of proteins, amino acids, and peptides that exhibit piezoelectric properties. In 1957, Fukada employed direct and reverse piezoelectric effects to examine the quantitative piezoelectric properties of bone.^[^ ^168^ ^]^ Studies have shown that the piezoelectric constant of bone is approximately one‐tenth that of quartz. The removal of collagen resulted in the loss of the piezoelectric properties of the bone, whereas the mineral content remained unaffected. Hydroxyapatite ceramics have been suggested to generate electrical energy through flexoelectricity, which is defined as the generation of electric charge due to a strain gradient independent of piezoelectric activity.^[^ ^169^ ^]^ Biological components in the human body that lack centrosymmetry, including biomolecular building blocks, tissues, and organs, demonstrate piezoelectric activity.^[^ ^170^ ^]^ The use of piezoelectric devices made from waste organs and tissues for energy harvesting is rare. Yin et al. utilized extracted human teeth as an active element in a piezoelectric nanogenerator for power generation. Piezoelectric nanogenerators made from human teeth demonstrate significant and stable power outputs, reaching an open‐circuit voltage of 0.9 V when subjected to 60 N of external stress owing to the piezoelectric characteristics of enamel and dentin. The piezoelectric nanogenerator exhibited mechanical durability after 1600 cycles of pressing and releasing, with no degradation in output observed. A piezoelectric device utilizing human teeth illuminated an LED for the first time, as highlighted in Figure 4V. A piezoelectric nanogenerator for energy harvesting employs extracted human teeth, promoting bionanotechnology to transform waste into profit for sustainable energy solutions.^[^ ^171^ ^]^

#### 4.2.4 Biodegradable Piezoelectric Polymers

Biodegradable polymers are materials engineered to work for a certain amount of time before deteriorating in a controlled manner, resulting in readily recyclable materials.^[^ ^172^ ^]^ There are two kinds of biodegradable polymers available: natural and synthetic. Natural polymers are classified into three categories: polysaccharides, polyamides, and polynucleotides. Plant‐derived polysaccharides such as cellulose and dextran, as well as animal‐derived polymers such as collagen and silk fibroin, are widely used in the field of transitory piezoelectric applications because of their biocompatibility and ability to be degraded by enzymes.^[^ ^173^ ^]^ In contrast to naturally occurring materials, synthetic biodegradable materials are often constructed from artificial polymers, which are composed of one or more structural units that are generally covalently connected.^[^ ^92^ ^]^ Furthermore, synthetic polymers with inherent dipoles may demonstrate piezoelectricity by modifying polymer chain configurations.^[^ ^92^ ^]^ Poly(L‐lactic acid) (PLLA),^[^ ^174^ ^]^ polyhydroxybutyrate (PHB),^[^ ^175^ ^]^ poly(3‐hydroxybutyrate‐co‐3‐hydroxyvalerate) (PHBV),^[^ ^176^, ^177^ ^]^ chitosan, and cellulose‐based polymers are common examples of biodegradable piezoelectric polymers.^[^ ^178^ ^]^

The piezoelectric response of poly‐L‐lactic acid (PLLA) and its prospective use in different electronic devices, such as sensors, wearable devices, and actuators, has sparked the interest of researchers in the field of biodegradable piezoelectric films.^[^ ^174^ ^]^ In this context, Lin et al. described a UV‐curable acrylate film packaged with a PLLA sensor that can be used to identify human behavior. Acrylic films with different percentages have good skin adhesion, ductility, and mechanical and surface properties. The developed sensor demonstrated exceptional application performance, including touch, swallowing, and wrist bending.^[^ ^179^ ^]^ Bioelectronics greatly benefit from biocompatible and biodegradable energy harvesters since they provide safe and alternative energy sources for human consumption. Tao et al. demonstrated a biodegradable piezoelectric nanogenerator (PENG) consisting of a polylactic acid sheet and an array of embedded diphenylalanine microrods, and their development procedure is highlighted in Figure 4VI. The PENG demonstrates efficiency, with an optimal output voltage of 1.78 V and a power density of 1.56 W m^−3^. At 60 °C for 25 days, the device fully dissolves in acid, alkali, and phosphate‐buffered saline solutions. The biodegradable PENG is an effective alternative for short‐term power requirements, ensuring minimal environmental impact.^[^ ^180^ ^]^

#### 4.2.5 Hybrid Piezoelectric Materials

Hybrid piezoelectric materials consist of organic and inorganic piezoelectric components at the nanoscale or microscale and exhibit synergistic properties that distinguish them from traditional materials.^[^ ^17^ ^]^ Peng et al.^[^ ^181^ ^]^ developed a hybrid piezomaterial by sandwiching single‐crystal BaTiO₃ films with PVDF‒TrFe layers, yielding a nanocomposite piezoelectric nanogenerator (PENG). The PENG exhibited a substantial output of 15.1 V, 2.39 µA, and a peak power density of 17.33 µW cm^−2^, surpassing devices constructed with pure PVDF‐TrFE or BTO nanoparticle‐doped PVDF‐TrFE. Banerjee et al.^[^ ^182^ ^]^ reported the synthesis of a metal‐doped potassium sodium niobate (KNN‐ZS) nanorod filler through a hydrothermal method, which demonstrated a high piezoelectric coefficient (≈95 pCN^−1^). The integration of 3 wt% KNN‐ZS into a PVDF matrix by electrospinning produced a nanocomposite fibrous web with superior structural and morphological properties. The resultant nanogenerator exhibited piezoelectric outputs of approximately 1.68 V and 0.216 µA. Significantly, when operating in both piezoelectric and triboelectric modes, the device produced outputs of approximately 25 V and 2.11 µA, respectively. This synergistic effect facilitates efficient energy harvesting, with the generated charge stored in capacitors to power small‐scale electronics, underscoring the potential of hybrid nanogenerators in sustainable energy applications. Tripathy et al.^[^ ^183^ ^]^ examined the piezoelectric properties of KNN integrated into PVDF and its copolymers—PVDF‐TrFE and PVDF‐HFP—through solution casting and corona poling. Among the composites, PVDF‐TrFE with 8 wt% KNN (PTK8) exhibited exceptional properties, including the highest β‐phase content (85%) and dielectric constant. The developed nanogenerator attained a maximum voltage output of approximately 20 V and a power density of 0.54 µW cm^−2^, surpassing alternative formulations. The results underscore the promise of KNN/PVDF‐TrFE hybrid composites for effective, compact, and environmentally sustainable energy harvesting systems in self‐powered electronics. To overcome the constraints of independent piezoelectric and triboelectric nanogenerators, Li et al.^[^ ^184^ ^]^ developed a hybrid tribo/piezoelectric nanogenerator (HTPENG) featuring an innovative “microsphere@nanofiber” architecture. This structural innovation markedly improved the energy harvesting efficiency, attaining a short‐circuit current of 8.066 µA, an open‐circuit voltage of 35.693 V, and a power density of 525.12 mW m^−2^—adequate to energize a light‐emitting diode in 20 s. This research illustrates the ability of structural engineering in hybrid nanogenerators to increase output efficiency, providing promising solutions for energizing portable and miniaturized electronic devices. A visualized tactile sensing electronic skin (VTSES) was developed by Wang et al.^[^ ^185^ ^]^ to replicate the multifunctional sensing abilities of human skin, incorporating PVDF‐TrFE, thin‐film transistor (TFT), and poly(l‐lactide) (PLLA) arrays. The PVDF‒TrFE layer serves as both a dynamic and thermal stimulus sensor and a dielectric capacitor for static sensing, interfacing with the TFT array to facilitate high‐resolution signal processing similar to neural activity. The PLLA layer facilitates dynamic slide detection and enhances biocompatibility. The VTSES system facilitates real‐time visualization of tactile data, including location, size, and texture of contact, presenting considerable opportunities for applications in artificial intelligence, robotics, and human‒machine interfaces.

Flexible pressure sensors are increasingly popular for wearable applications because of their versatility and conformability.^[^ ^186^ ^]^ Mu et al.^[^ ^187^ ^]^ presented an innovative, flexible, integrated pressure sensor (FIPS) consisting of a silicone rubber matrix infused with piezoelectric PAN/PVDF powders and conductive silver‐coated glass microspheres. The sensor, with a PAN/PVDF‐to‐rubber mass ratio of 4:5, attains a maximum output voltage of 49 V. It demonstrates exceptional linearity (R^2^ ≈ 0.986) within the range of 0–800 kPa, accompanied by high sensitivities of 42 mV kPa^−1^ and 0.174 nA kPa^−1^. The device exhibited a rapid response (43 ms), durability exceeding 10 000 cycles, and proficient detection of human motion, including gait and finger flexion. These findings underscore the FIPS's ability for sophisticated health monitoring and motion sensing applications.

## 5 Fabrication Techniques for Piezoelectric Devices

Piezoelectric materials are extensively utilized in wearable and implantable electronics owing to their scalability benefits. Multiple material processing techniques have been developed and optimized to fabricate piezoelectric materials and devices, ranging from solution‐based approaches to precision printing methodologies and advancing to sophisticated manufacturing protocols. Each fabrication route of these methodologies presents distinct advantages from many angles: structure control, scalability, and device integration capabilities. This section outlines the fabrication methodologies employed in the development of bioelectronics for wearable and implantable electronics, systematically analyzes processing fundamentals and essential postprocessing protocols, and provides a representative and comprehensive overview of the current state‐of‐the‐art techniques. Moreover, a summarized overview of the fabrication techniques is provided in **Table** **4**.

**Table 4:** Summary overview of Fabrication Techniques.

Technique	Principle	Fabrication Speed	Film Thickness Control	Structural Quality	Throughput	Key Features	Applications	Limitations	Reference
Electrospinning	Electric field induces liquid jet formation	≈2.6 g/h (HTES system)	Moderate (≈100 nm–5 µm)	High (uniform nanofiber morphology)	Low (1–100 mg h^−1^ lab‐scale)	Continuous nanofibers, cost‐effective	Wearable electronics, filtration, and biomedical applications	Sensitive to humidity, temperature, and high voltage required	[237, 238, 239, 240]
Spin Coating	Centrifugal force distributes coating	High (seconds per layer)	Good (2–10 µm typical)	High (surface uniformity)	Medium (batch processing)	Uniform thin films, rapid processing	Thin‐film electronics, optoelectronics	Limited to flat substrates, edge effects	[241, 242, 243]
Additive Manufacturing (3D Printing)	Layer‐by‐layer material deposition	FDM: ≈50–150 mm s^−1^; SLA: ≈20–36 mm h^−1^	High (≈50–100 µm per layer)	Moderate–High (resolution‐dependent)	Low (limited by print speed and resolution)	Complex 3D geometries, customizable	Prototyping, bioelectronics, piezoelectric devices	Limited material range, slower large‐scale production	[244, 245, 246]
Screen Printing	Ink forced through patterned mesh	High (up to 2–3 m^2^ s^−1^)	Low (≈10–100 µm layers)	Moderate (mesh‐dependent)	High (large‐scale production)	Simple, scalable, suitable for mass production	Sensors, printed electronics, displays	Limited resolution, requires postprocessing/sintering	[221, 247, 248, 249]
Inkjet Printing	Drop‐on‐demand digital patterning	Medium–High (0.01–0.5 m^2^ s^−1^)	Digital, maskless, low material waste	Flexible sensors, displays, and organic electronics	Strict ink viscosity and surface tension requirements	Digital, maskless, low material waste	Flexible sensors, displays, organic electronics	Strict ink viscosity and surface tension requirements	[228, 250, 251]

### 5.1 Solution‐Based Processing

#### 5.1.1 Electrospinning

**Figure 5:**
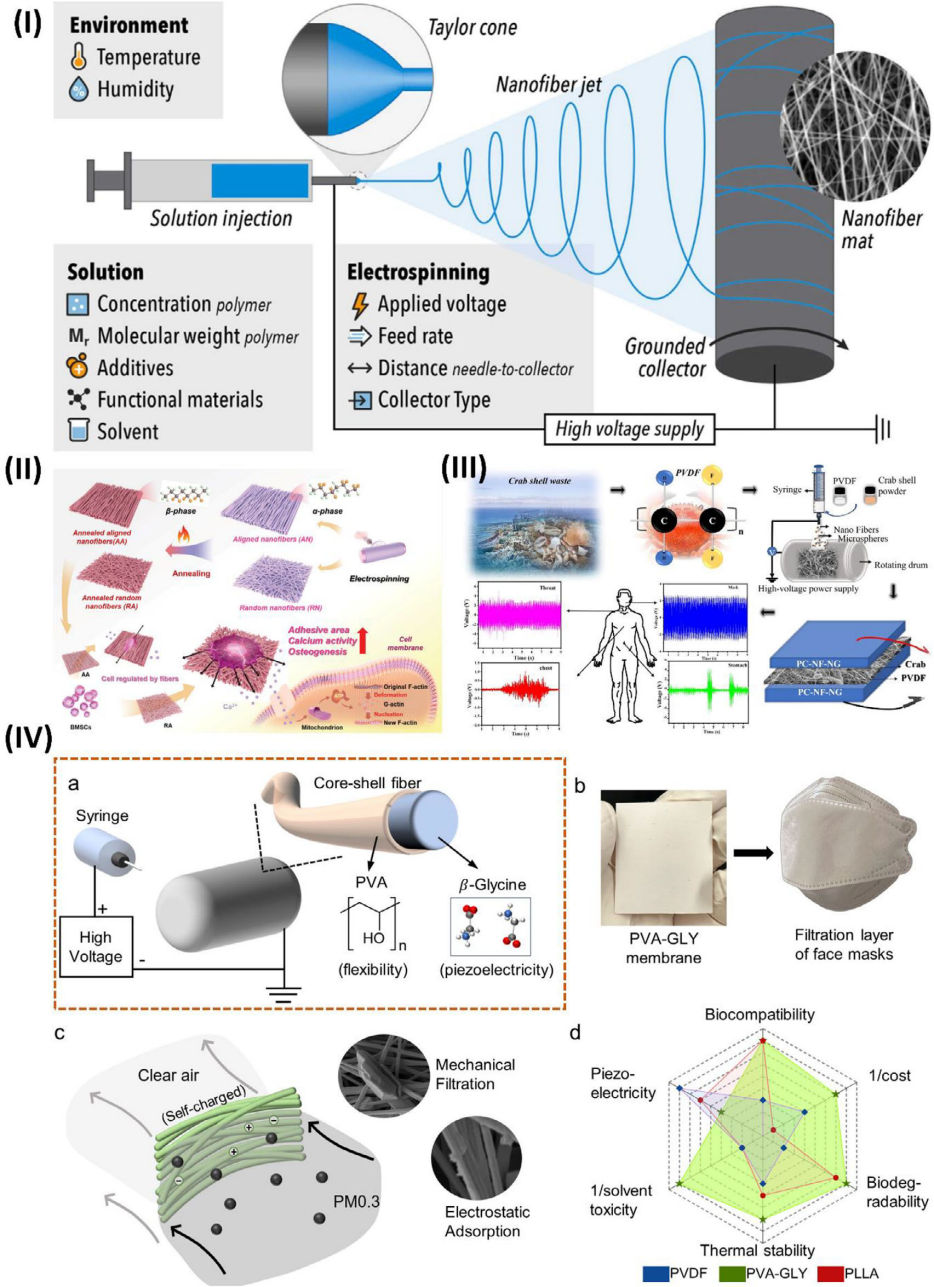
Application of piezoelectric materials by electrospinning techniques. I) Illustration of electrospinning setup with environmental parameters and solution effects, reproduced under CC by a 4.0 license.^[^ ^204^ ^]^ Copyright 2022 Elsevier. II) Schematic diagram of the fabrication of annealed aligned and random PVDF nanofibers and the mechanism of BMSC differentiation on nanofibers, reproduced under CC by a 4.0 license.^[^ ^207^ ^]^ Copyright 2023, Wiley VCH GmbH. III) Illustration of extraction and electrospinning of the carb shell waste‐doped PVDF‐based PENG and its application in the movement of the neck, mask, chest, and abdomen, reproduced with permission.^[^ ^208^ ^]^ Copyright 2024, Elsevier. IV) Schematic representation of the mask using a PVA‐GLY nanofiber membrane. a) Electrospinning of PVA‐GLY. b) Facial mask development from an electrospun PVA‒GLY membrane. c) Mechanical and electrostatic adsorption mechanisms of the electrospun PVA‒GLY membrane. d) Radar map illustrating the benefits of the PVA‒GLY membrane over the PVDF and PLLA membranes, reproduced with permission.^[^ ^209^ ^]^ Copyright 2024, Elsevier.

Electrospinning is an electrohydrodynamic method whereby an electric field is introduced to cause a liquid droplet to produce a jet. The jet elongated and stretched, forming nanofibers owing to heightened surface tension.^[^ ^203^ ^]^ The electrospinning approach involves a straightforward and cost‐effective apparatus consisting of a spinneret, a syringe infusion pump, a high‐voltage power source, and a conductive collector, as illustrated in **Figure** **5**I.^[^ ^204^ ^]^ The liquid is first delivered to the spinneret by a syringe infusion pump. The liquid produces droplets in the spinneret due to its high surface tension.^[^ ^205^ ^]^ A high‐voltage power source, which may be alternating (AC) or direct (DC), facilitates electrification. Upon electrification, surface charges of the same polarity create electrostatic repulsion, resulting in the deformation of the droplet into a geometric configuration termed a Taylor cone. A charged jet is then expelled from the cone, demonstrating linear progression and experiencing pronounced whipping movements due to bending instabilities. The jet solidification process occurs as the jet narrows to smaller diameters, leading to the deposition of solid fibers on the collector surface.^[^ ^206^ ^]^

Recently, electrospun nanofibers have been extensively used in wearable and implantable electronics, as shown by Wu et al.,^[^ ^207^ ^]^ who conducted a comprehensive investigation of the interaction of electrospun PVDF nanofibers with stem cells. Furthermore, annealing, fiber orientation, and stem cell differentiation were thoroughly studied in Figure 5II. This study explored the role of the annealing temperature and nanofiber orientation in enhancing the osteogenic potential of PVDF‐based membranes. Polarization under optimized thermal conditions significantly increased the β‐phase content, improving the piezoelectric performance. Randomly aligned (RA) PVDF nanofibers nearly doubled the degree of osteogenesis of untreated fibers and outperformed aligned (AA) fibers. The enhanced osteogenic differentiation of bone marrow stem cells (BMSCs) on RA fibers was attributed to both the piezoelectric effect and the 3D microenvironment, which promoted greater cell adhesion, cytoskeletal deformation, and calcium ion activity. Real‐time calcium signaling and vinculin expression confirmed that nanofiber orientation and piezoelectricity synergistically regulate stem cell behavior. This work provides valuable insights into material–cell interactions and highlights the potential of PVDF nanofibers in bone tissue engineering and regenerative medicine.^[^ ^207^ ^]^

Divya et al. augmented PVDF nanofibers by integrating crab shell powder, hence improving the performance output of PENGs to exceed 19 V. Furthermore, the PENG is applicable to the neck, mask, chest, and abdomen movement, as shown in Figure 5III.^[^ ^208^ ^]^

Wang et al. fabricated biodegradable piezoelectric membranes by electrospinning polyvinyl alcohol (PVA) infused with glycine (GLY), as shown in Figure 5IV(a). As shown in Figure 5IV(b), the membranes used as facemasks exhibit exceptional filtering efficacy above 97% for 10 h via both mechanical and electrostatic adsorption mechanisms Figure 5IV(c). Additionally, the efficacy of the biodegradable mask is shown in Figure 5IV(d).^[^ ^209^ ^]^

#### 5.1.2 Spin Coating

**Figure 6:**
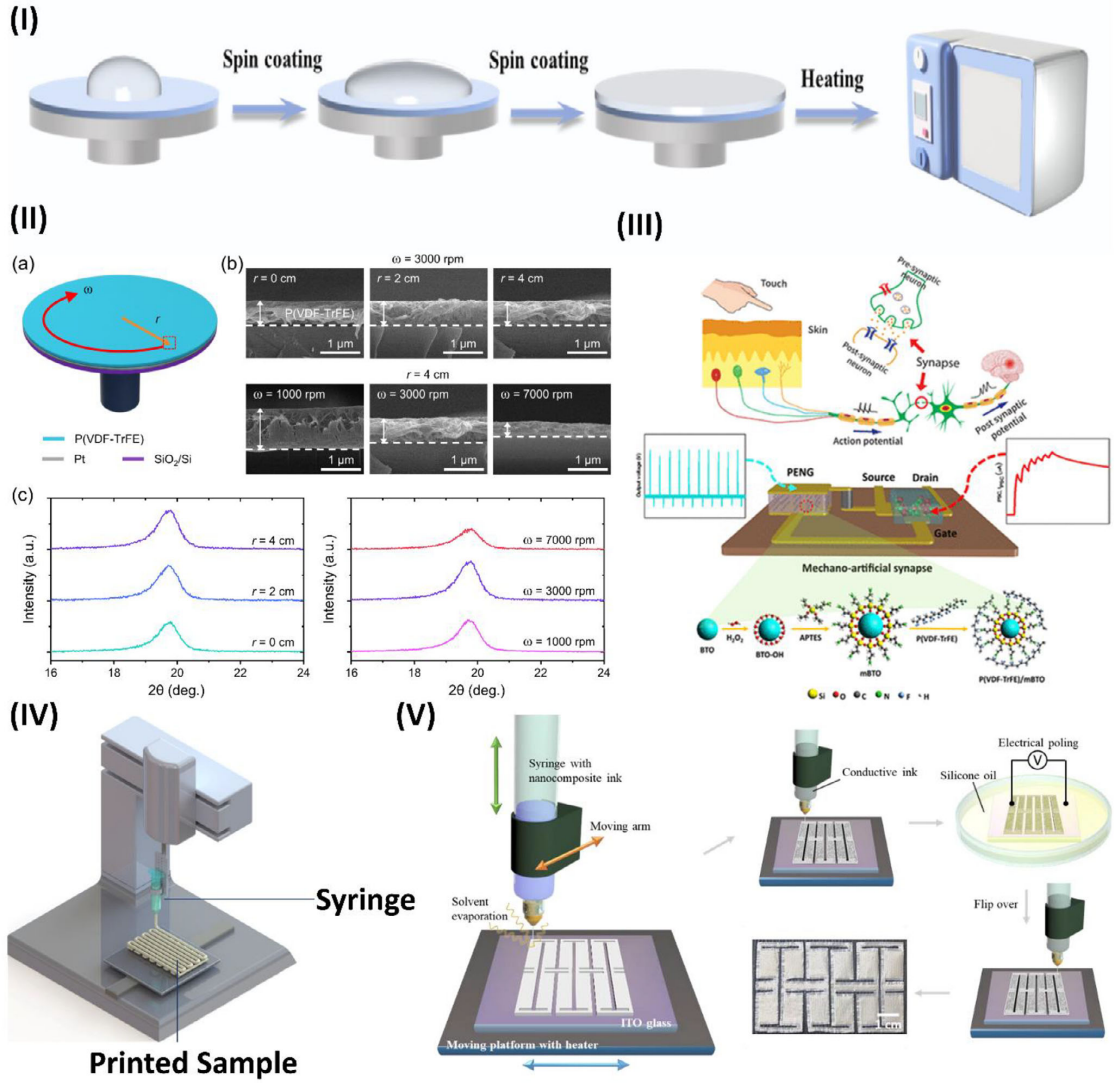
Application of piezoelectric materials via spin coating and 3D printing techniques. I) Illustration of the spin coating technique, reproduced with permission.^[^ ^210^ ^]^ Copyright 2024 Wiley Periodicals LLC. II) Spin coating process investigation of PVDTrFE, a, b) diagram illustrating the spin coating process of P(VDF‐TrFE) films and cross‐sectional scanning electron microscopy (SEM) of P(VDF‐TrFE) films. c) XRD of P(VDF‐TrFE) films with respect to distance from the center and different rpms under CC by a 4.0 license.^[^ ^213^ ^]^ Copyright 2023 MDPI, Basel, Switzerland. III) Graphical illustration of P(VDF‐TrFE)/modified‐BTO‐based piezotronic artificial mechanoreceptor, reproduced with permission.^[^ ^214^ ^]^ Copyright 2024, American Chemical Society. IV) Schematic illustration of the 3D printing technique reproduced under open access CC BY licenece.^[^ ^218^ ^]^ Copyright 2024, Elsevier ltd. V) Schematic representation of the manufacturing process for the entirely 3D‐printed PENG, reproduced with permission.^[^ ^220^ ^]^ Copyright 2020, Elsevier.

Spin coating is a method for applying uniform, thin layers or films onto specific surfaces. A small amount of precursor material is placed at the center of the substrate, which can rotate either slowly or at a steady pace. The substrate is spun at desired speeds that align with the desired film thickness, utilizing a centrifugal force to distribute the coating material consistently, as illustrated in **Figure** **6**I.^[^ ^210^ ^]^ Spin coating equipment is known as spin coaters or spinners.^[^ ^211^, ^212^ ^]^

Jeong et al. conducted a detailed examination of the parameters involved in spin coating and their effects on the structural, mechanical, piezoelectric, and electrical properties of PVDF‐TrFE. First, as the distance from the center increases, the rotational speed enhances the piezoelectric properties. However, the piezoelectric properties decrease with increasing speed beyond a certain point. Furthermore, Figure 6II(a,b) illustrates the spin coating process of P(VDF‐TrFE), with thickness measurements obtained through SEM analysis. Figure 6II(c,d) shows the XRD crystallinity of the (PVDF‐Trfe) film at various distances from the center and varying RPMS.^[^ ^213^ ^]^ Do et al. reported an artificial mechanoreceptor by doping (PVDF‐TrFE) with modified barium titanate (mBTO) through spin coating. Figure 6III simulates the mechanoreceptor of piezotronics alongside a biological tactile system. In conclusion, this advancement can potentially significantly open new arenas for tactile perception technology.^[^ ^214^ ^]^

### 5.2 Advanced Manufacturing Methods

#### 5.2.1 Additive Manufacturing

Additive manufacturing, often called 3D printing technology, has attracted considerable attention in multiple fields because of its efficiency, precision, cost‐effectiveness, optimal material utilization, and ability to produce intricate three‐dimensional structures.^[^ ^215^ ^]^ A range of 3D printing technologies has been utilized to fabricate devices based on piezoelectric materials, including Fuse deposition modeling, selective laser sintering, and stereolithography.^[^ ^216^ ^]^ The continuous progress in smart interaction and energy technology has generated considerable interest in piezoelectric devices, which are recognized for their unique stress‒electricity response and conversion capabilities. Nonetheless, the limited structural diversity and lack of a theoretical framework considerably impede the prospects for performance improvements and the future relevance of these devices.^[^ ^217^ ^]^ Figure 6IV shows a schematic representation of the 3D printing process, wherein the slurry contained in the syringe is extruded into filaments under pressure and assembled into the intended configuration by a three‐dimensional moving platform.^[^ ^218^ ^]^ Song et al. investigated a range of innovative piezoelectric‐enhanced porous structures created via powder bed fusion of polymers via a laser beam (PBF‐LB/P). The theoretical analysis and analog simulation established the influence mechanism of the pore structure size characteristics, such as the pore shape and line thickness, on the piezoelectric performance. Moreover, the energy harvester exhibited an impressive piezoelectric response when subjected to impacts from fingers, hands, feet, and bicycle tyres. This study introduces a novel method for creating advanced piezoelectric structures while greatly expanding the potential applications and capabilities of piezoelectric devices.^[^ ^219^ ^]^ Zhou et al. created a stretchable kirigami piezoelectric nanogenerator (PENG) using barium titanate (BaTiO_3_) nanoparticles, a P(VDF‐TrFE) matrix, and electrodes made from silver flakes, all produced through a fully 3D printable process, as highlighted in Figure 6V. A meticulously designed modified T‐joint‐cut kirigami structure was created to attain a nonprotruding, high structural stretchability performance, tackling the out‐of‐plane displacement challenges often encountered in conventional kirigami structures and thereby enhancing the pressing‐mode functionality of a kirigami‐structured PENG.^[^ ^220^ ^]^

### 5.3 Printing Approaches

#### 5.3.1 Screen Printing

**Figure 7:**
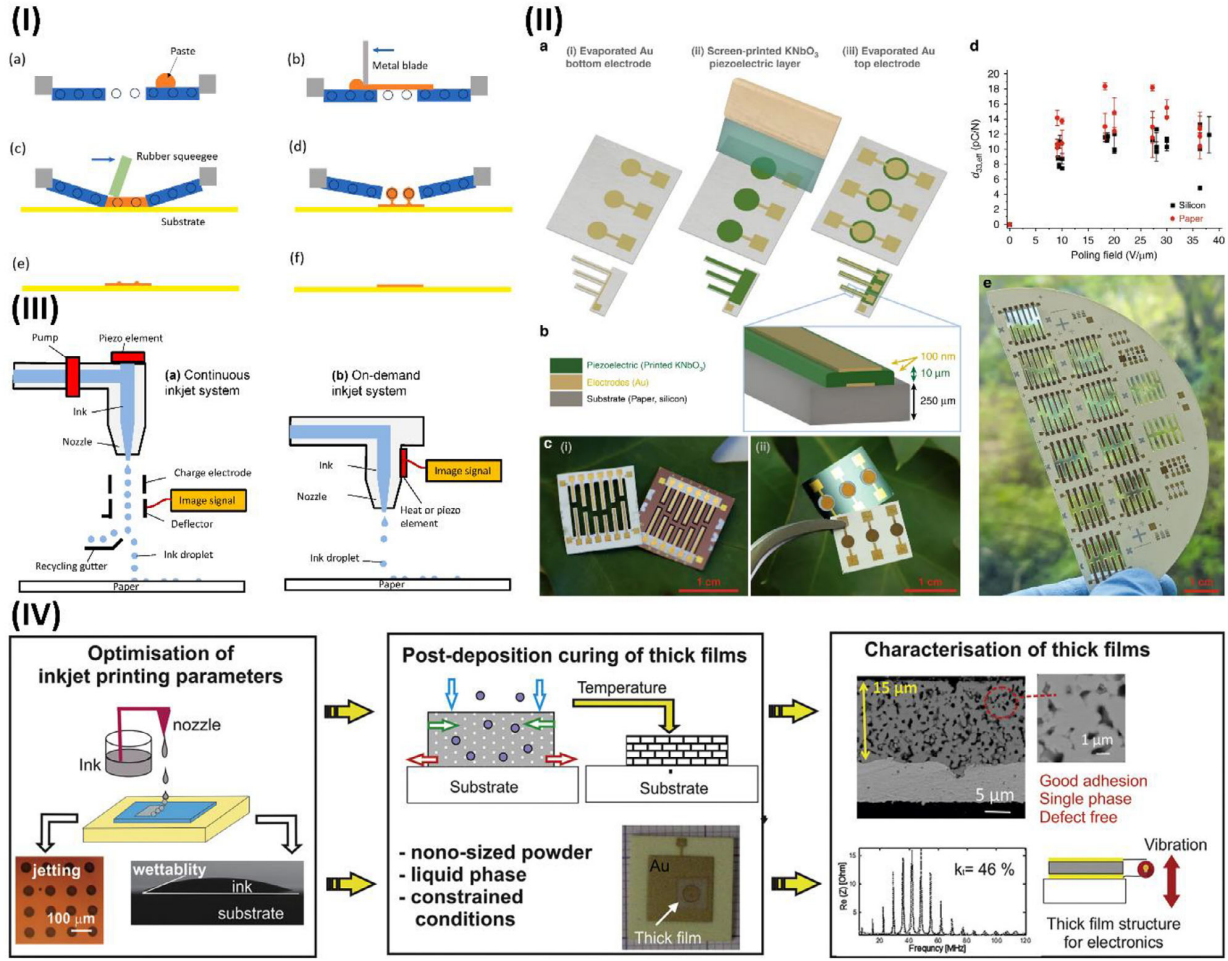
Screen printing and inkjet printing techniques for piezoelectric devices for biomedical applications. I) Illustration of the screen printing mechanism, reproduced with permission.^[^ ^223^ ^]^ Copyright 2024 Elsevier. II) presents an overview of the device architectures, fabrication processes, scalability, and key piezoelectric responses of the fabricated devices, all of which are elaborated upon in subsequent sections. a) Streamlined fabrication process for these devices, and b) cross‐section of the completed device stack, including estimated layer dimensions. c) Finalized printed devices for the parallel plate capacitor and cantilever structures. d) Investigation of the d_33_ coefficient over various poling voltages. e) Visual of paper substrates for cantilever devices manufactured on a wafer, reproduced under CC by a 4.0 license.^[^ ^225^ ^]^ Copyright 2023, Springer Nature. III) Schematic illustration of a continuous inkjet printer a) and on‐demand inkjet printer b), reproduced under CC by a 4.0 license.^[^ ^227^ ^]^ Copyright 2017, MDPI, Basel, Switzerland. IV Visuals of the optimization process, postdeposition process and characterization validations of thick film fabrication using lead‐zirconate‐titanate via inkjet printing, reproduced with permission.^[^ ^230^ ^]^ Copyright 2020 Elsevier ltd.

Screen printing technology has attracted considerable interest because of its simple production method and remarkable scalability. As a result, this technology has been used in the fabrication of various organic electronic devices for several applications, including biosensors, logic circuits, and amplifiers, which use organic electrochemical transistors, piezoelectric sensors, and electrochromic displays.^[^ ^221^, ^222^ ^]^ The printing process, as depicted in **Figure** **7**I, commenced with the application of paste onto the screen adjacent to the pattern area Figure 7I(a). A metal blade is employed to apply a thin layer over the exposed area, referred to as the “flooding bar” process, Figure 7I(b). A rubber squeegee is subsequently employed to press the paste into the exposed region and remove the excess paste, Figure 7I(c). Upon the passage of the rubber squeegee over the exposed area, the mesh retracts and separates from the paste, creating a liquid bridge between the thread and the substrate Figure 7I(d). The liquid bridge ultimately thins and ruptures, resulting in liquid beads on the printing pattern, Figure 7I(e). The paste on the substrate will achieve self‐leveling until a relatively uniform condition is attained, Figure 7I(f).^[^ ^223^ ^]^

Screen printing has been used mainly to fabricate sensors to detect electrophysiological impulses. The advancement of solution‐processed, biodegradable piezoelectrics represents significant progress in mitigating global electronic waste. Piezoelectric printing technologies face limitations because of the elevated sintering temperatures required for perovskite manufacturing.^[^ ^224^ ^]^ Monroe et al. reported that lead‐free printed piezoelectric devices operating at lower temperatures are suitable for integrating environmentally friendly substrates and electrodes. The process, device architecture, poling results and device images are highlighted in Figure 7 (IIa‐e). Figure 7II illustrates a streamlined manufacturing procedure for these devices, whereas Figure 7IIb presents a cross‐section of the completed device stack, with estimated layer dimensions. Figure 7IIc shows the finished printed devices for both the parallel plate capacitor and cantilever constructions. Effective d_33_ response attained as a function of the poling field for capacitor configurations, assessed via the Berlincourt technique, as described in Figure 7IId, and cantilever devices fabricated at wafer sizes on paper substrates, as described in Figure 7IIe. Developing printable biodegradable piezoelectrics facilitates the production of solution‐processed, environmentally sustainable electronics.^[^ ^225^ ^]^

#### 5.3.2 Inkjet Printing

Inkjet printing is a digital, noncontact method that enables the direct patterning of extensive regions without the need for a physical mask. Moreover, digital patterning reduces production costs, allowing rapid design alterations without necessitating new masks and promoting a more adaptable processing flow and seamless layer superposition.^[^ ^226^ ^]^

Inkjet printers use a piezoelectric diaphragm to generate pressure waves that eject ink droplets through a nozzle. In continuous inkjet printing, Figure 7III(a), droplets (0.5–1 µL) are produced rapidly (80–100 kHz) and directed by deflection plates, with unused droplets recirculated. In contrast, in demand‐mode printing Figure 7III(b), smaller droplets (2–500 pL) are ejected at lower frequencies (up to 30 kHz) without recirculation, resulting in a simpler design and reduced ink waste.^[^ ^227^ ^]^

This technology is appropriate for various production sizes and requires a cheaper initial investment than other printing methods do. Ink usage and material waste are negligible and may generate patterned thin films. A significant limitation of this approach is that inks must satisfy precise rheological criteria, such as viscosity and surface tension, which must remain within rigorous parameters.^[^ ^228^ ^]^ Piezoelectric energy can be generated through repeated small bending deformations, utilizing harvesters on plastic surfaces. The intricate manufacturing process and size constraints hinder the commercialization of piezoelectric self‐powered technology. Lim et al. presented a straightforward inkjet‐printed flexible piezoelectric energy harvester utilizing a BaTiO_3_ hybrid material. The noncontact inkjet method enables the printing of a flexible, large‐area piezoelectric hybrid film and silver electrode layers on a flexible substrate, eliminating the need for high‐temperature annealing or complex transfer processes. An inkjet‐printed energy harvester produces a maximum open‐circuit voltage of 7 V, a short‐circuit current of 2.5 µA, and an adequate output power of 5 µW through periodic mechanical deformation. This method facilitates self‐powered systems and flexible electronics based on inorganic materials.^[^ ^229^ ^]^ Kuscer et al. investigated thick film fabrication using lead‐zirconate‐titanate via inkjet printing. Various waveforms were utilized to ascertain the optimal dimensions and configuration of the droplets. Moreover, illustrative visuals of the optimization process, postdeposition process, and characterization validations are listed in Figure 7 (IV). The reported findings have significant implications for economically viable and environmentally friendly practices in printing piezoelectric materials, which are essential for various electronic devices, including actuators, sensors, piezoelectric energy harvesters, and transducers.^[^ ^230^ ^]^

The discussed fabrication methods possess notable advantages and limitations regarding structural control, scalability, and device integration for piezoelectric polymer applications. Electrospinning is a preferred method for producing piezoelectric nanofibers, offering precise control over diameter and superior structural integrity. The scalability of electrospinning is limited because of its intricate structure, making it more suitable for small‐scale applications. It has numerous applications, including wearable and implantable biosensors.^[^ ^231^ ^]^ Conversely, spin coating moderately regulates the film thickness, which can fluctuate on the basis of the rotational velocity. It is exceptionally scalable and suitable for extensive surface coverage; however, its integration capabilities are somewhat restricted, mainly in thin‐film applications.^[^ ^232^ ^]^ Additive manufacturing is distinguished by exceptional structural control, enabling the creation of intricate and tailored geometries. It is exceptionally scalable, rendering it appropriate for mass production, and excels in device integration by facilitating the amalgamation of various materials and components within a single assembly.^[^ ^38^, ^233^ ^]^ Screen printing provides moderate regulation of layer thickness; however, it does not achieve high detail resolution. Nonetheless, it is ideally suited for high‐volume production and integrates efficiently into basic sensor and circuit designs.^[^ ^234^ ^]^ Ultimately, inkjet printing offers moderate structural control with accurate patterning, albeit limited by the characteristics of the inks employed. It offers significant scalability with minimal material waste and is ideally suited for the integration of patterned films onto flexible substrates, positioning it as a formidable option for flexible and implantable electronics.^[^ ^235^, ^236^ ^]^

## 6 Characterization Techniques for Piezoelectric Materials

It is crucial to determine the physicochemical characteristics to clarify the relationship between the structure and properties of piezoelectric materials. Standard characterization techniques for analyzing piezoelectric materials span fundamental structural analysis, direct piezoelectric response measurements, chemical composition analysis, and thermal behavior studies. These advanced characterization methods enable comprehensive insights into the material's structure, piezoelectric properties, surface chemistry, and thermal stability, which are essential for optimizing device performance.

### 6.1 Piezoelectric Response Analysis

#### 6.1.1 Piezoelectric Force Microscopy (PFM)

**Figure 8:**
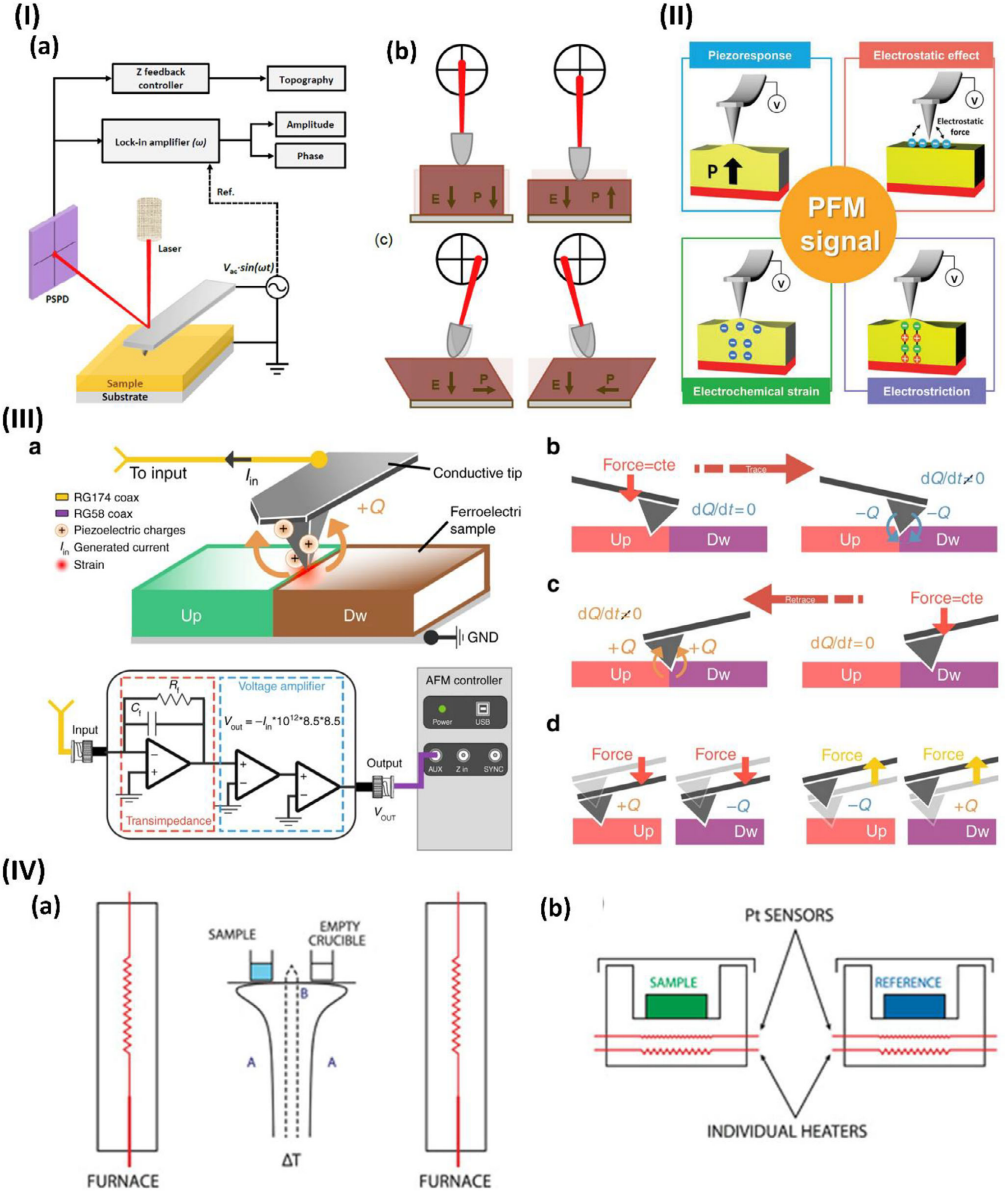
Advancements in PFM, DSC characterization. I) Schematic illustration of the PFM setup and its principles of b) vertical and c) lateral PFMS, reproduced with permission.^[^ ^255^ ^]^ Copyright 2016, Elsevier B.V. II) Illustration of various PFM signals reproduced under CC BY 4.0 license.^[^ ^256^ ^]^ Copyright 2020, WILEY‐VCH Verlag GmbH & Co. KGaA, Weinheim. III) Diagram of the measurement apparatus and an outline of the proposed nanoscale representations of piezogenerated charges in lead zirconate titanate (PZT), bismuth ferrite (BiFO3), a thick single crystal of periodically poled lithium niobate (PPLN), and a force‐produced current in these materials. a) The apparatus used to measure the piezoelectric charge generated by the material utilizes a designated current‒voltage transimpedance amplifier. b) When a single domain polarization is scanned, the force remains invariant, resulting in no current flow. As the tip moves across different domains, a current is generated due to the continuous force, while the d_33_ value reverses its sign. c) At the domain wall, the induced current may be shown as the tip entering one domain, charging it, departing from the opposing domain, and discharging it. The indication of charge generation is dictated by the scanning direction, specifically, whether the tip transitions from an up domain to a down domain (Q < 0) or a down domain to an up domain (Q > 0). d) The spectroscopic sweep model is generated when the tip performs a force‐versus‐distance sweep. When the tip exerts stress on the sample, a strain is generated. Upon the relaxation of the force, unstraining occurs. The actions of straining and unstraining generate either negative or positive charges depending on the polarization of the domain, which are reproduced under a CC BY 4.0 license.^[^ ^258^ ^]^ Copyright 2017, Springer Nature. IV) Schematic illustration of heat flux differential scanning calorimetry (DSC) (a) and power‐compensated differential scanning calorimetry (DSC), reproduced with permission.^[^ ^261^ ^]^ Copyright 2018 Canadian Society for Chemical Engineering.

PFM, or piezoelectric force microscopy, is a high‐resolution subset method of atomic force microscopy used to examine the piezoelectric characteristics of materials at the nanoscale. The PFM mechanism is straightforward and consists of a conductive tip integrated with an AFM system. During the experiment, the application of an AC voltage induces mechanical deformations on the surface of piezoelectric materials, which are measured by an AFM cantilever.^[^ ^252^ ^]^ The findings are primarily interpreted on the basis of the oscillation amplitude and phase of the cantilever. Nonetheless, PFM is a very sophisticated technology that requires meticulous calibration, and the environmental impacts on the tip nodes present significant obstacles in its processing.^[^ ^253^ ^]^ PFM operates in derivative imaging mode, meaning that it derives electromechanical information from primary topographic imaging. A lock‐in amplifier is used to extract the electromechanical signal by comparing the input voltage signal V_in_ = Acos(ωt+ϕ) with a reference signal V_ref_ = Bcos(ωt). The output signal contains two components: a DC term 1/2 ABcos(ϕ) and an AC term at twice the frequency ½ ABcos(2ωt+ϕ). From this, the amplitude and phase images of the piezoelectric response are extracted, providing insights into the material's local electromechanical behavior, as illustrated in **Figure** **8**I(a).^[^ ^254^, ^255^ ^]^ In piezoresponse force microscopy (PFM), the amplitude (A) signal reflects the strength of the piezoelectric response, whereas the phase (ϕ) signal indicates the polarization direction. Together, they define the overall piezoresponse. By analyzing the cantilever deflection, the vertical PFM measures out‐of‐plane polarization through vertical movements, and the lateral PFM captures in‐plane polarization via lateral deflections, as highlighted in Figure 8I(b,c).^[^ ^255^ ^]^

Moreover, electrostatic influences and electrochemical strain may affect the PFM hysteresis loop. The Coulombic electrostatic force between the sample surface and the scanning probe microscope (SPM) cantilever or tip causes an electrostatic effect, which is unavoidable in PFM and other voltage‐modulated SPM modes. It is difficult to exclude these impacts from the PFM results. Surface potential electrostatic effects, especially the contact potential difference, may produce phase flipping in hysteresis loops and mislead PFM visuals about the polarization direction even when no switching occurs. The visuals of the PFM signals are highlighted in Figure 8II.^[^ ^256^ ^]^ Electrostatic activity from charge injection may change the equilibrium surface potential. Thus, the electrostatic effect of charge injection may cause a hysteresis loop and contrast in ferroelectric‐like PFM images. Increasing the spring constant of the SPM cantilever while measuring at higher AC frequencies and compensating for the surface potential with an external voltage minimized the electrostatic force.^[^ ^256^, ^257^ ^]^

Despite the prevalence of piezoelectric and ferroelectric materials in our everyday lives, many questions about their physics remain unanswered. Evaluation at the nanoscale is the only way to better understand piezoelectric and ferroelectric materials. Therefore, Gomez et al. provided a technique for directly quantifying the piezoelectric coefficient d_33_ via atomic force microscopy, as illustrated in Figure 8III. The experimental configuration is intended to quantify piezoelectric charge production with a very sensitive current‐to‐voltage transimpedance amplifier characterized by a low input‐bias current and a bandwidth of 4–5 Hz (Figure 8III(a)). When a probe tip traverses a singular ferroelectric zone under constant tension, no current is observed (Figure 8III(b)), owing to the homogeneous piezoelectric response. When the tip traverses domain barriers, a current is induced due to the change in the sign of the piezoelectric coefficient d_33_. The orientation of the induced charge is contingent upon the scanning direction—transitioning from an up domain to a down domain generates a negative charge, whereas the opposite transition yields a positive charge (Figure 8 III(c)). Furthermore, in a force‐versus‐distance spectroscopic sweep (Figure 8III(d)), the application and release of force create strain and unstrain in the material, producing charges whose polarity corresponds to the domain's polarization. This study demonstrated the nanoscale representations of piezogenerated charges in lead zirconate titanate (PZT), bismuth ferrite (BiFO_3_), a thick single crystal of periodically poled lithium niobate (PPLN), and a force‐produced current in these materials. The method's reliability is further bolstered by an equally precise measurement of the considerably larger d_33_ of the PZT.^[^ ^258^ ^]^

An electromagnetic‐piezoelectric hybrid nanopositioner for atomic force microscopy (AFM) with a serial‐parallel‐kinetic design was reported by Tan et al. The xy‐stage is powered by normal‐stressed electromagnetic actuators (NSEAs) for long‐range planar scanning, whereas a piezoelectric actuator (PEA) on top tracks topography at high speed. A unique flexure mechanism improves motion guiding and decouples the xy‐stage, allowing for quicker AFM imaging by increasing the x‐axis resonance frequency. The analytical model was validated via finite element analysis. The prototype exhibited motion ranges of 94.4 µm (x), 102.8 µm (y), and 5.22 µm (z), with resonance frequencies of 735 Hz (x), 650 Hz (y), and 6340 Hz (z), respectively. The feedback controllers provided accurate high‐speed tracking, whereas AFM imaging demonstrated the system's ability to scan wide areas at high rates.^[^ ^259^ ^]^

### 6.2 Thermal Analysis

#### 6.2.1 Differential Scanning Calorimetry (DSC)

Differential scanning calorimetry (DSC) is an excellent analytical method for determining the physical characteristics of materials. DSC allows the evaluation of the glass transition temperature and the determination of melting, crystallization, and mesomorphic transition temperatures.^[^ ^260^ ^]^ Differential scanning calorimetry (DSC) can be used to determine the specific heat capacity and phase transition enthalpies across an extensive temperature range (−150 to 1800 °C). It is generally used in small amounts (1–100 mg), making it suitable for diverse applications. The predominant method, heat‐flux DSC, measures voltage differentials between thermocouples located under the sample and reference crucibles and is often configured in an ABA arrangement to mitigate noise. There are two varieties: point detectors, which are characterized by high sensitivity and rapid reactions, and plate detectors, which provide superior contact consistency and calibration proficiency, as illustrated in Figure 8IV(a). Conversely, a power‐compensated DSC maintains uniform temperatures between the sample and reference by modulating the electrical heating power, often via Peltier devices, as demonstrated in Figure 8IV(b). This technique is very sensitive and suitable for microcalorimetry. However, it typically functions at reduced temperature ranges.^[^ ^261^ ^]^

DSC plays a crucial role in piezoelectric materials by elucidating the transition between ferroelectric and dielectric phases. Furthermore, several piezoelectric materials exhibit identical temperature transition phases, posing challenges during the investigation of the DSCs of materials.^[^ ^262^ ^]^ Moreover, poly(vinylidene fluoride) (PVDF) is a well‐known piezoelectric polymer that possesses crystalline phases—α, β, and γ—each contributing uniquely to its piezoelectric characteristics. Precise characterization of these phases is essential for optimizing the piezoelectric performance of PVDF in bioengineering applications.^[^ ^263^ ^]^ Purushothaman et al. developed a comprehensive multitechnique approach that combines Fourier transform‐infrared spectroscopy (FTIR), Raman spectroscopy, wide‐angle X‐ray scattering (WAXS), and differential scanning calorimetry (DSC) to analyze the crystalline structure of PVDF. DSC analysis revealed two distinct glass transitions: a lower transition (T_gl_ ≈ −40 °C) associated with amorphous phase mobility and an upper transition (T_gu_ ≈ 30–60 °C) linked to interfacial mobility between the crystalline and amorphous regions. An additional endothermic peak (T_msc_ ≈ 50–70 °C) was attributed to the melting of secondary crystals, which disappeared upon melt recrystallization. The crystallization behavior is phase dependent: the β‐phase forms near 80 °C, the α‐phase forms near 130 °C, and the γ‐phase forms above 160 °C. Melting temperatures (T_m_) further distinguish the phases, with α and β melting at approximately 167 °C and γ melting at approximately 183 °C. However, (T_m_) alone is not a reliable phase identifier owing to its sensitivity to factors such as molar mass, branching, polydispersity, crystallite size, and experimental conditions. This study underscores the necessity of using multiple complementary techniques for precise phase quantification in PVDF.^[^ ^264^ ^]^

Recent investigations into the piezoelectric characteristics of poly(vinylidene fluoride) (PVDF) have transitioned from only measuring the β‐phase concentration to examining its crystallization morphology.^[^ ^265^, ^266^ ^]^ In this context, Ye et al. developed PVDF films exhibiting a pure β phase, elevated crystallinity, and enhanced interfacial regions that were produced by ultradrawing and high‐pressure thermal annealing methods. Differential scanning calorimetry (DSC) was used to examine the crystallization behavior of the PVDF films at different tensile ratios. The results indicated that moderate tensile ratios (3 and 6) increased crystallinity via enhanced molecular chain alignment. Nevertheless, higher tensile ratios (9 and 12) resulted in diminished crystallinity and broader melting peaks due to the disruption of crystalline regions and the emergence of defects. Following annealing, the crystallinity and crystallinity of the films improved, especially in the ultradrawn samples, which experienced better chain orientation and less entanglement. These structural enhancements facilitate more efficient crystal formation and stability of the β phase.^[^ ^267^ ^]^

Poly‐L‐lactic acid (PLLA) is recognized as a biodegradable piezoelectric material for regenerative medicine and tissue engineering, particularly when it is electrospun to improve fiber alignment and chain orientation.^[^ ^174^ ^]^ Despite its promise, the mechanisms that control the piezoelectric capabilities of PLLA have not been thoroughly researched. The effects of chiral purity, molecular weight, and crystallinity on the piezoelectric response of PLLA were systematically investigated by Rentero et al. Several PLLA microfibers with high alignment were created, indicating that the piezoelectric performance is largely dependent on the crystallinity and dipole orientation along the polymer chains. The most significant piezoelectric response was obtained in samples with high chiral purity and low D‐isomer concentration, which also had the highest crystallinity at 52%. In contrast, samples with high D‐isomer concentrations had lower crystallinity and piezoelectric efficiency. Thermal treatment during cold crystallization, Tcc 100 °C, increased the melting temperature and piezoelectric sensitivity by crystallizing the metastable oriented phase formed during the electrospinning process. DSC revealed a glass transition at 60 °C, followed by cold crystallization (T_cc_ = 60–90 °C) and melting peaks based on chiral purity. Physical aging was responsible for a significant endothermic peak at the glass transition temperature (Tg), although the final melting temperature (T_m_) was more closely related to the chiral purity than to the molecular weight.^[^ ^268^ ^]^

## 7 Applications

Rapid advancements in piezoelectric materials have accelerated wearable technologies for physiological monitoring and energy harvesting.^[^ ^269^, ^270^ ^]^ These materials are ideal for implanted and wearable electronics because of their capacity to convert mechanical energy into electrical energy.^[^ ^271^ ^]^ Utilizing these materials, researchers can increase the efficiency and efficacy of wearable and implantable technology, resulting in superior healthcare solutions that are both practical and adaptive. This section discusses several applications, including optimal energy harvesting systems, smart clothing, and health monitoring devices in wearable and implantable applications. A summarized overview of wearable piezo applications is listed in **Table** **5**.^[^ ^272^ ^].^

**Table 5:** Summary overview of piezoelectric applications in wearable devices.

Device	Piezoelectric Material	Doping/Enhancement Material	Mechanism	Fabrication Method	Performance	Application	References
ZnO@C/PVDF Electrospun Membrane	PVDF (polyvinylidene fluoride)	ZnO nanoparticles & nanorods with carbon coating (ZnO@C)	Enhanced piezoelectric effect via β‐phase PVDF and ZnO@C interaction	Electrospinning with in situ electrical poling and mechanical stretching	Max power density: 384.8 µW cm^−3^ Max output voltage: 19.9 V	Wearable energy harvesting, breathable, and waterproof textiles	[310]
WS₂ QDs‐PANI Cotton Fabric Nanogenerator	WS₂ quantum dots (QDs)	Conductive polyaniline (PANI) and PMMA matrix	Piezoelectric effect from WS₂ QDs and conductive PANI electrodes	Hydrothermal synthesis of WS₂ QDs, dip‐coating of PANI on cotton, embedding in PMMA	Output voltage: 60 V Current density: 302 nA cm^−2^	Wearable energy harvesting, piezoelectric touch sensors, robust under humidity and strain	[311]
PANI‐ZnS/PDMS Hybrid Piezo‐Triboelectric Nanogenerator (HPTNG)	ZnS nanoplates	Mulberry‐shaped PANI nanoparticles (0–2.5 wt%)	Hybrid piezoelectric and triboelectric effect enhanced by PANI doping	Hydrothermal synthesis of ZnS, polymerization of PANI, composite formation with PDMS	Max output voltage: ≈180 V Power density: ≈280 µW cm^−2^	Self‐powered electronics, wearable sensors, motion‐responsive energy harvesting	[312]
PVDF‐Covered ZnO Nanorod Array Nanogenerator	ZnO nanorods	PVDF conductive polymer	Piezoelectric effect enhanced by PVDF‐ZnO interaction	Hydrothermal growth of vertically aligned ZnO nanorods on ITO‐coated PET, PVDF coating	High current output under bending, pressing, and body motion	Mechanical energy harvesting from body motion, implantable, and wearable electronics	[313]
PDA@BTO/CA Hybrid Nanogenerator	Barium titanate (BTO) nanoparticles	Polydopamine (PDA) in cellulose acetate (CA) matrix	Piezoelectric effect enhanced by PDA‐BTO‐CA interaction	Electrospinning of PDA@BTO/CA nanofibers	‐ 40% increase in voltage output due to PDA ‐ Durable over 15000 cycles	Biodegradable, flexible biosensors for wearable human motion monitoring	[314]
PC‐NFs PENG (Crab Shell‐PVDF Nanogenerator)	PVDF nanofibers	Crab shell powder nanofillers (CS‐NFs, 1.5 wt%)	Piezoelectric effect enhanced by biobased nanofillers	Electrospinning of PVDF with CS‐NFs	‐ Max output voltage: 19 V ‐ Charges 2.2 µF capacitor to 2 V in 180 s	Eco‐friendly, biocompatible wearable sensors for health monitoring and energy harvesting	[315]
PVDF/MXene Confined Orientation Nanofiber PENG	PVDF nanofibers	MXene nanosheets	Confined orientation structure enhances β‐phase and force transfer	Electrospinning of PVDF/MXene nanofibers	‐ Piezoelectric coefficient: 61.7 pCN^−1^ ‐ Response time: 14 ms ‐ Pressure sensitivity: 19.29 mV kPa^−1^	High‐performance wearable sensors, mechano‐electrodeposition, and self‐powered sensing	[316]
CNF/PVDF Nanofiber‐Based PENG	PVDF nanofibers	Carbon nanofibers (CNFs) from polyacrylonitrile	CNFs enhance β‐phase formation and conductivity	Electrospinning under an electric field with CNF incorporation	Stable over 12 000 cycles at 20 N, 5 Hz	Acoustic, air, and water wave sensing; biomechanical energy harvesting; healthcare monitoring	[317]
Zn‐Car_TPHG with Cu‐HHTP/Thy Bio‐MOF Hybrid Nanogenerator	Zn‐Car_MOF (Zn(II)‐carnosine)	Cu‐HHTP MOF loaded with thymol (Thy)	Tribo‐piezoelectric hybrid effect + ROS generation for antibacterial action	MOF synthesis, PDMS patterning, device integration	‐ Output: 131 V at 100 kPa ‐ Pressure range: 1 Pa–100 kPa	Environmental energy harvesting, biomedical sensors, and self‐powered antibacterial systems	[318]
Nd5‐PENG (Neodymium‐Doped ZnS Nanoplates)	ZnS nanoplates	Neodymium (Nd) doping (1–5 mol%)	Piezoelectric enhancement via rare‐earth doping	Hydrothermal synthesis of Nd‐doped ZnS nanoplates	‐ Output: ≈42 V ‐ Power density: 102.75 µW cm^−2^	Self‐charging power systems, wearable electronics, and biomedical motion sensors	[319]
Reconfigurable BTO‐CNF Paper‐like PENG	Barium titanate (BTO)	Cellulose nanofibrils (CNF)	Reconfigurable piezoelectric film with low internal resistance	Film casting and polarization strategy	‐ Optimal at 25 wt% BTO	Self‐powered smart microsystems, fall alarms, biodegradable, and flexible electronics	[320]
FCF‐PENG (Sb‐Doped BaTiO₃/PDMS)	BaTiO₃	Antimony	Sb doping enhances piezoelectricity	Hydrothermal + PDMS	28 V, 1.5 µA, 1.6 mW m^−2^	IoT‐based impact sensors	[321]
PVDF/CA & PVDF/CMCNa Supersonic‐Blown PENGs	PVDF	CA or CMCNa	γ and β‐phase enhancement	Supersonic solution blowing	12.5–28.6 V, 31 µW cm^−2^	Smart sensors, health monitoring	[322]
Bamboo Microfibril‐PVDF Nanofiber PENG	PVDF	Bamboo microfibrils	Biomass‐enhanced β‐phase	Electrospinning	29.75 V, 503.8 µW cm^−3^	Smart wheelchairs	[323]
Dual‐Modal PENG + PPG SAS Detection System	PVDF	PPG sensor	Two‐stage detection: PENG triggers PPG	Integrated system + AI	99.59% accuracy	SAS monitoring	[324]
FPI/Ni Porous Foam Hybrid Sensor	FPI	Ni nanoparticles	Synergistic piezo‐triboelectric coupling	One‐step foaming	49 V, 10 000 cycles	Motion & speech detection	[325]
Surface‐Engineered BaTiO₃/PDMS HBNG	BaTiO₃	PDMS (micropyramid)	Surface‐enhanced hybrid effect	Lithography + casting	92 V, 11 µA	Sustainable energy harvesting	[326]
PAN/K‐BTO Composite Nanofiber Sensor	PAN	KH550‐modified BTO	Enhanced piezoelectricity via dispersion	Electrostatic spinning	12.33 V, 4.93 µA, 1.63 V N^−1^	Flexible motion sensors	[327]
MoSe₂/PVDF Nanocomposite PENG	PVDF	MoSe₂ nanosheets	2D material‐enhanced piezoelectricity	Sandwich‐structured casting	56 V, 680 µW cm^−2^, 3.73 V kPa^−1^	IoT, wearable energy harvesting	[328]
PVDF/BaTiO₃/f‐MWCNT Hybrid PENG (PBC11 & PBC12)	PVDF	BaTiO₃ + f‐MWCNTs	Hybrid filler‐enhanced β‐phase and dielectric properties	Electrospinning	69 V (PBC11), 63 V (PBC12), 55 and 45 µW cm^−2^, 2.34 and 2.31 µA	Real‐time exercise/fatigue monitoring, wearable electronics	[329]
PVDF‐HFP/CNC Composite Yarn PENG	PVDF‐HFP	Cellulose nanocrystals (CNC)	CNC‐induced β‐phase formation and mechanical enhancement	Electrospinning into yarns	21.2 V @ 20 N, 0.5 Hz	Touchscreen gloves, motion sensing in subzero temperatures	[330]
Woven PVDF & Cu‐PVDF Yarn Fabric Sensor	PVDF	Copper‐coated PVDF yarns	Strain‐to‐voltage responsiveness via woven structure	Loom weaving	Detects 30°/90° knee flexion, walking/running	Knee joint monitoring, health and motion tracking	[331]
CF/ZnONR & CF/ZnONT Textile PTENGs	ZnO nanorods/nanotubes	Carbon fabric (CF)	Hybrid piezoelectric and triboelectric effects	Microwave hydrothermal growth (100 mM Zn(NO₃)₂ + HMTA)	High sensitivity to pressure, impact, vibration	Wearable pressure/impact sensors, soft robotics, e‐skin	[331]
SO‐PEG (Stretched Oriented PVDF Nanofiber Generator)	PVDF	Oriented nanofiber alignment	Enhanced β‐phase and strain transfer	Electrospinning + mechanical stretching	27 V, 0.93 V N^−1^, 95.1% β‐phase, Tr = 93 ms, Tf = 84 ms, >2000 cycles	Wearable sensors for small and large motion detection	[332]
Post‐Drawn & Annealed Nylon‐11,11 Textile PENG	Nylon‐11,11	None (postprocessing)	Improved crystalline orientation and crystallinity	Electrospinning + postdrawing + annealing	21.5 V, 800 nA, 1.88 mW m^−2^ @ 80 MΩ, 266 mV kPa^−1^, 13.99 nA kPa^−1^	Biomechanical energy harvesting, gesture and motion monitoring	[333]
Terbium‐Doped Lead‐Free Perovskite Composite Fiber PENG	Lead‐free double perovskite	Terbium (Tb), aramid & PPS fibers	Piezoelectric and luminescent multifunctional composite	Energy‐efficient crystallization + fiber modification	50 V output, 10 ms response, 91.6% recognition accuracy	Smart textiles, motion recognition, environmental sensing	[334]

### 7.1 Wearable Electronics

Rapid advancements in microelectronic sensor systems have resulted in the robust development of embedded miniaturized wearable electronics. However, the low power supply with relatively ample dimensions in these microsystems limits their life span and broad range of practical applications.^[^ ^36^, ^273^ ^]^ In this regard, piezoelectric materials are ideal for wearable electronics owing to their low cost and intrinsic energy conversion characteristics, which are highly precise and accurate. They can monitor highly sensitive physiological data by converting mechanical energy into electrical current across multiple domains via their inherent piezoelectric effect. This section discusses various health monitoring systems, e‐textiles, and micropower energy harvesters that use microcomposite‐based piezoelectric materials.^[^ ^274^, ^275^ ^]^

#### 7.1.1 Health Monitoring Devices

Piezoelectric health monitoring systems enable noninvasive detection of various human physical and physiological parameters. Additionally, piezoelectric sensors have shown promising development in the early detection and long‐term monitoring of cardiovascular diseases, including heart rate, blood pressure, and strain movements. Moreover, piezoelectric sensors can directly monitor arterial pulse waveforms and atherosclerosis at preliminary stages, reducing fatal vascular events and decreasing mortality rates.^[^ ^276^, ^277^ ^]^

Mohsin et al.^[^ ^278^ ^]^ fabricated a biodegradable and flexible PLLA/Gly‐based wearable device using a piezoelectric film to address secondary surgery and e‐waste management while continuously monitoring physiological signals. They reported that the sensor can detect signals from the carotid and radial arteries and tactile stimuli such as swallowing, coughing, and wrist and elbow movements. Despite several advancements in biodegradable electronics, their study introduced an innovative piezoelectric material that is easy to fabricate, flexible, and highly efficient, with easy degradability in phosphate‐buffered saline at 37 °C. Moreover, the pressure sensor exhibited a response time of 10 ms with a high sensitivity of 13.2 mV kPa^−1^ and good mechanical stability.

Yan et al.^[^ ^279^ ^]^ designed a wearable piezoelectric sensor based on polyacrylonitrile (PAN) nanofibers for motion detection and personal health monitoring. They used MXene and a polydopamine‐modified zinc oxide‐based dual filler to develop a piezoelectric PAN/MXene/PDA@ZnO‐5 (PMPO) sensor. The sensor exhibited a wider linear range with a higher sensitivity of 285.56 V N^−1^ and good durability of 3000 loading‒unloading cycles with a robust response time of 49 ms and recovery time of 40 ms. Moreover, the unique flexibility owing to the fiber structure when interfacing with the body enabled monitoring of minute physiological movements and varied motions. The applications of health monitoring devices using piezoelectric material for vital signs of physiological biomarkers continuously enable proactive health management; for example, piezoelectric sensors integrated into wristbands can track heart rate variability and detect potential cardiac issues.^[^ ^280^, ^281^ ^]^ Similarly, wearable patches equipped with piezoelectric materials can monitor respiratory patterns, providing critical data for patients with respiratory conditions.^[^ ^282^, ^283^ ^]^

**Figure 9:**
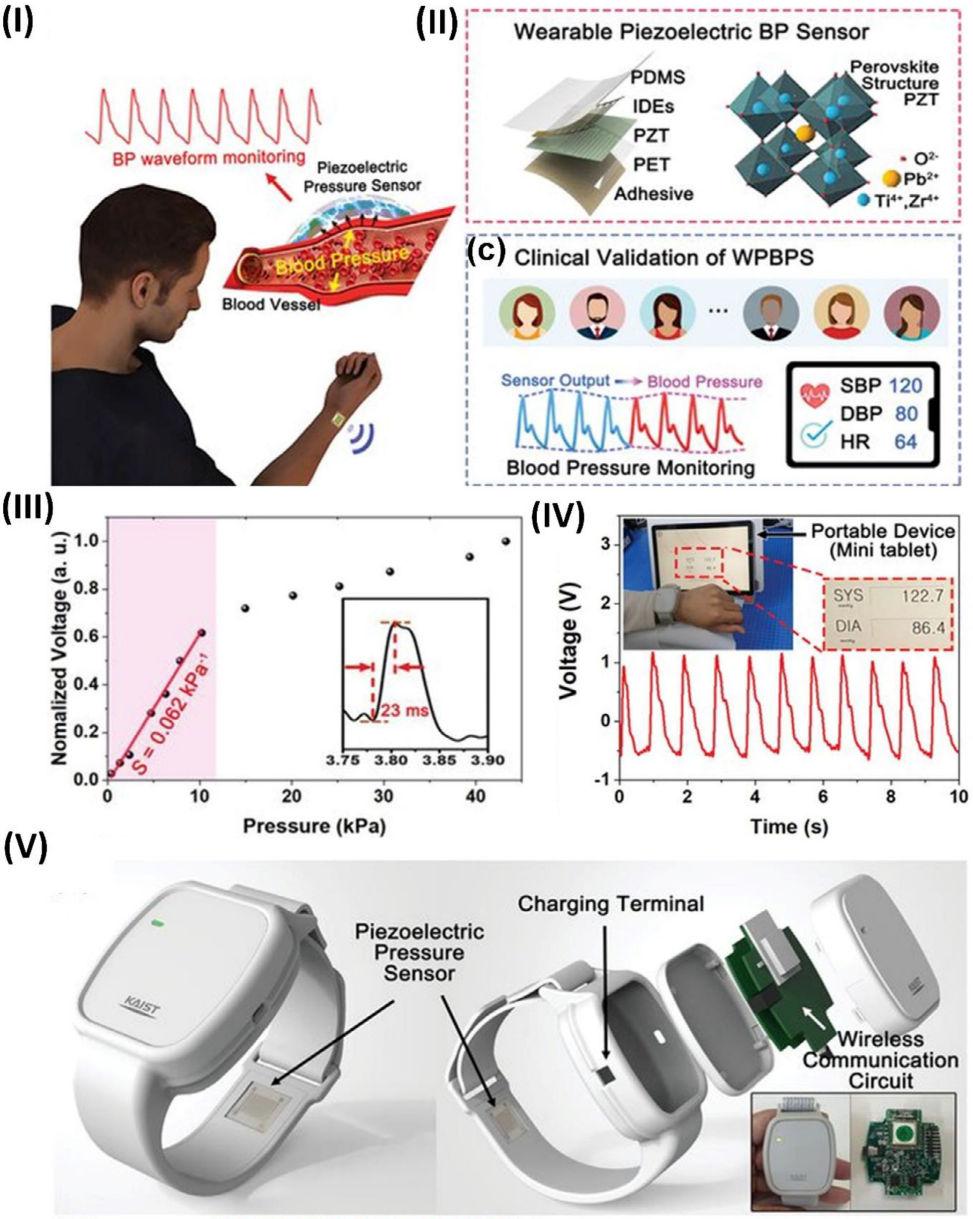
Piezoelectric material application in wearable electronics I) Schematic representation of the complete concept of a wearable piezoelectric blood‐pressure sensor (WPBPS). A piezoelectric sensor is attached to the user's skin to detect arterial pulse signals, which can be converted into BP values. II) The upper insets display a layout of the flexible piezoelectric sensor. III) Conceptual image of clinical validation in contrast to the commercial sphygmomanometer. IV) Output voltage normalized as a function of applied pressure and robust response time of the pressure sensor (inset). V) Schematic design illustration of the developed WPBPS wristwatch. Reproduced with the permission.^[^ ^284^ ^]^ Copyright 2023, Wiley.

Seongwook et al.^[^ ^284^ ^]^ designed a wearable piezoelectric blood pressure sensor (WPBPS) to continuously monitor noninvasive arterial pressure (CNAP) from the human wrist artery. A piezoelectric sensor responded sensitively when external pressure was applied to the arterial pulse. It generates an output signal as a BP wave, followed by the conversion of pulse pressure data into the BP values in **Figure** **9**I. A high‐quality sensor is fabricated using piezoelectric Pb(Zr_0.52_Ti_0.48_)O_3_ (PZT) perovskite material. The quality of the piezoelectric thin film is an important parameter in optimizing sensor performance; therefore, the perovskite thin film was crystallized by annealing at a relatively high temperature and then transferred to the PET substrate via a laser lift‐off (LLO) procedure. The upper and lower layers of the piezoelectric sensor were made from the PDMS layer, which encapsulated the PZT and contributed to increasing the signal‐to‐noise ratio by decreasing the capacitance. Moreover, the PDMS also enhanced the conformal contact between the skin and the sensing interface. The PZT membrane was intentionally placed away from the neutral plane via a PET substrate Figure 9II to increase the stability and sensitivity of the designed sensor. Figure 9III revealed the conceptual image of clinical validation in contrast to the commercial sphygmomanometer, the US FDA approved to validate the accuracy of BP calculated via the transfer function of the WPBS. The validation study was performed on 35 participants who had undergone screening, with ages ranging from 20–80 years, and the mean differences between the commercial sphygmomanometer and WPBPS were −0.89 ± 6.19 and −0.32 ± 5.28 mmHg for systolic blood pressure (SBP) and diastolic blood pressure (DBP), respectively. The authors also observed the output traits of piezoelectric sensors using different applied pressures. The piezoelectric sensor generated an open‐circuit voltage ranging from 0.07–2.6 V under a compression pressure of 0.4–43 kPa. The output voltage was further normalized in this range to determine the linearity and sensitivity of the sensor. Figure 9IV was calculated at 0.062 kPa^−1^ for pressures less than 10 kPa.

The authors also designed a WPBPS‐mounted wristwatch containing a piezoelectric sensor for pulse waveform monitoring. The detachable sensor unit was designed for easy replacement in the case of sensor failure. The battery was recharged via the charging terminal on the watch head, and wireless communication was embedded inside the head of the watch, as illustrated in Figure 9V. They optimized the pulse waveforms from the wireless circuit, which discerned the unique features of arterial pulse waveforms, including the dicrotic notch and late systolic peak. They monitored the pulse position of the radial artery by attaching a piezoelectric sensor and placing it on the watch to measure the BP accurately. The inset shows a photograph of the user's beat‐to‐beat pulse monitoring and corresponding BP values in real time through a designed WPBPS wireless communication circuit. Implementing a CNAP‐based comfortable and conformal device is a promising solution for accurate wearable sphygmomanometers for CVD diagnosis.

Guan et al.^[^ ^285^ ^]^ developed self‐sustaining piezoelectric nanogenerators (PENGs) via melt copolymerized composite films (PZT/MFC@PVA) composed of polyvinyl alcohol, microfibrillated cellulose, and lead zirconate titanate. Sixty percent (wt/wt%) silver conductive paste was used to reduce the viscosity and ensure the connections of the PZT/MFC@PVA printed electrodes, resulting in increased surface resistance. Interdigital electrode electric fields laterally polarized the electric domains of the PZT nanoparticles. The sensitivity of the interdigital electrode PENG was measured to be 0.0724 V·kPa^−1^ within a dynamic stress range of 0.23–5 kPa, demonstrating the monitoring of different physiological signals and strains.

Moreover, piezoelectric hydrogel‐based sensors^[^ ^286^ ^]^ have significantly advanced noninvasive physiological signal monitoring. Cost‐effective multichannel wireless sensing devices that effectively collect data and enhance the use of wearable sensors have been developed more recently. In a multisignal acquisition system, the low‐voltage output of these sensors allows noise to obscure the output signal. Fu et al.^[^ ^287^ ^]^ developed a multichannel wireless sensing system using a flexible, biocompatible piezoelectric hydrogel sensor and proprietary signal processing and transmission components. The piezoelectric device can wirelessly track the body's physiological data in real time. In conclusion, this research enhances piezoelectric hydrogel‐based wireless multichannel sensing devices and may be advantageous in several intricate scenarios.

The advanced features of health monitoring devices using piezoelectric materials have allowed the development of more sensitive and accurate sensors. An increase in the composition and structure of materials, such as composites and nanomaterials, increases the sensitivity of these sensors to detect subtle alterations in the body's physiology.^[^ ^288^, ^289^ ^]^ Enhancing the biocompatibility and long‐term stability of piezoelectric materials for wearable applications remains two significant challenges, notwithstanding their potential.^[^ ^290^ ^]^

#### 7.1.2 Smart Fabrics and E‐Skins

Integrating textiles with electronic components facilitates the development of multifunctional garments, resulting in significant advancements in wearable technology. Piezoelectric materials are well aligned with e‐textiles, offering low power consumption and responding to multiple stimuli to produce a wide range of voltages ranging from millivolts to hundreds of volts. The development of these piezoelectric smart fabrics has relied upon the utilization of piezoelectric materials.^[^ ^291^ ^]^ Therefore, researchers are attracted to developing smart fabrics that utilize piezoelectric fibers to respond to motion and mechanical stress. Ainul et al.^[^ ^292^ ^]^ enhanced structural health monitoring by reducing the EMI with a new bond utilizing a Sn‐Ag alloy for sensor integration. They examined the effects of disbonding on bond endurance and impedance‐based signaling under environmental stressors. The experimental data indicated that a durable thermoactivated Sn‒Ag alloy bonds the structure to the piezoelectric transducer. Compared with those based on epoxy, the bonds formed with this substance exhibited superior resistance to temperature, chemical, and moisture. This characteristic enables the creation of garments that can detect and respond to physiological signals and movements. Shukla et al.^[^ ^293^ ^]^ investigated hybrid piezoelectric materials composed of graphene, gold, silver nanowires, and silver. Their study aimed to examine the piezoelectric capabilities of hybrid materials to increase the efficacy of energy harvesting by improving production processes. Shukla's research illustrated the use of these materials in the advancement of autonomous intelligent wearable devices within the Internet of Things (IoT) paradigm. Moreover, real‐time input from piezoelectric sensors in smart fabrics aided fitness training and rehabilitation.^[^ ^294^ ^]^ Similarly, muscle activity monitoring gear can help athletes perform better and avoid injury.^[^ ^295^ ^]^

**Figure 10:**
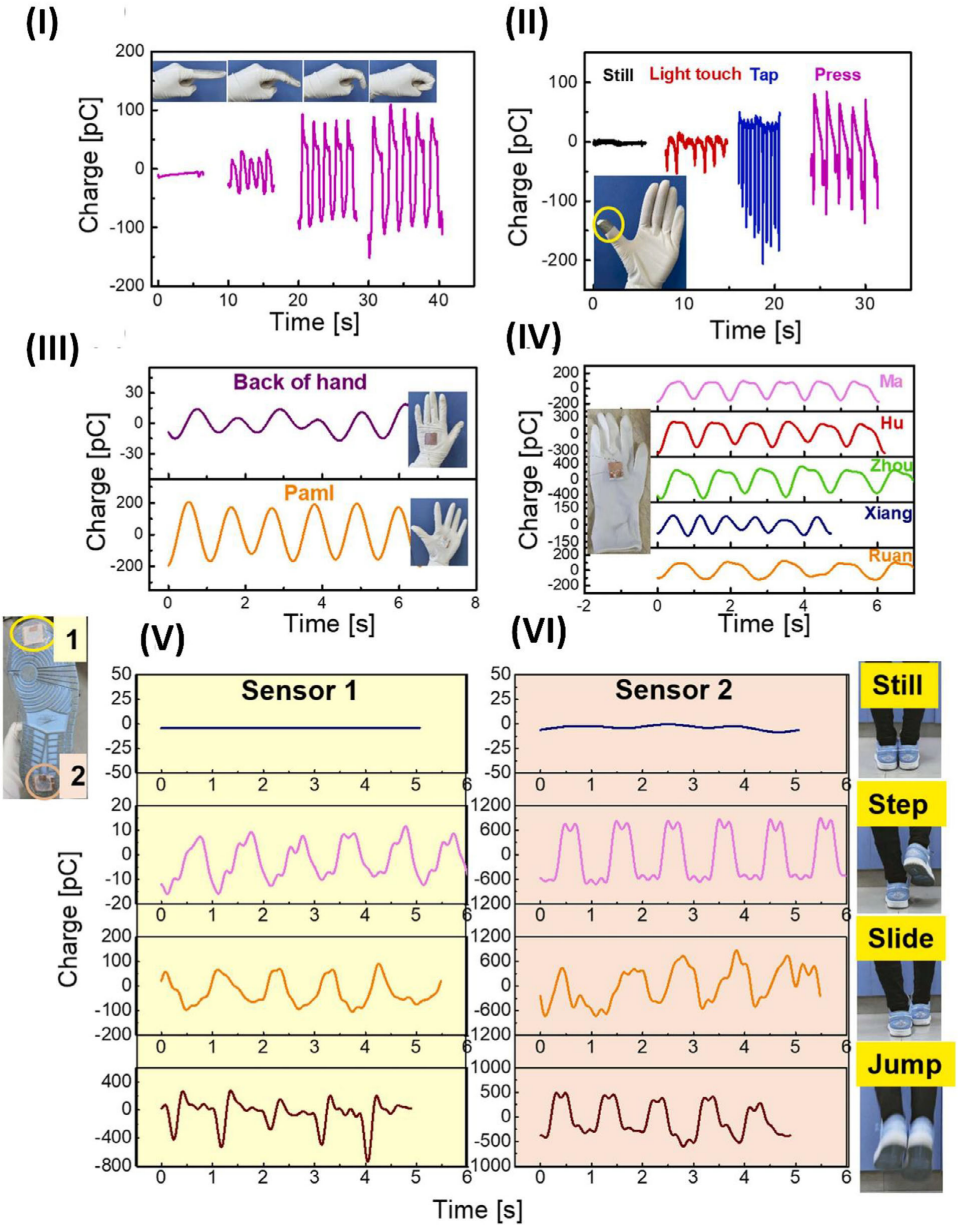
Examples of DS‐PLA wearable applications include I) the sensor's electrical reaction to finger flexion, demonstrating its applicability for wearing devices; II) variations in charge signals corresponding to various contact intensities (still, mild touch, tap, push), illustrating tactile sensibility. The inset depicts a sensor affixed to a finger. III) Electrical reaction to hand motions (opening and closing) when affixed to the dorsal side of the hand or palm, accompanied by inset photos depicting sensor placements. IV) Electric impulses were recorded from many participants performing identical hand motions. V, VI) Recognition of foot motions via the DS‐PLA sensor. Reproduced with the permission.^[^ ^296^ ^]^ Copyright 2022, Elsevier B.V.

Ma et al.^[^ ^296^ ^]^ developed a disposable sensor using a biodegradable polylactic acid piezoelectric film (DS‐PLA), which serves as both a pressure and tensile sensor. The sensor deteriorated in deionized water at 170 °C after 11 hours, promoting sustainable development. The DS‐PLA sensor has shown exceptional compressive sensitivity (up to 1000 pCN^−1^) and an extensive pressure detection range (0.03–62 kPa), resulting in sustained functionality after 1.08 million cycles. Compared with conventional sensors, it enhances tensile sensitivity. Wearable DS‐PLAs track human movements when affixed to the skin, making them suitable for advanced wearable biosensors and implantable biomedical devices, and providing maintenance‐free and eco‐friendly alternatives. As shown in **Figure** **10**I, the designed DS‐PLA was coupled to the finger to monitor the finger bending angle. The sensor could monitor finger bending at different angles by analyzing the changes in charge corresponding to various strain extents of the developed sensor. Similarly, a 3.14 cm^2^ sensor was attached to a human thumb to monitor tactile signals. Figure 10II shows that a larger finger press caused a higher signal intensity. The different characteristic signals were obtained from different thumb movements, aiding robots and disabled patients in applying the right and optimized amount of force on different objects.

The sensor also monitored hand shutting and opening motion owing to hand muscle bending when mounted on the back of the hand or palm. The signal generated by palm movement was greater than that generated by the back of the hand, owing to the more significant muscle contraction of the palm during hand opening and shutting (Figure 10III). In addition, the electrical signals generated by DS‐PLA showed good reproducibility while the hand was opened and shut by different volunteers, as illustrated in Figure 10IV. The designed sensor was also mounted on shoes and has potential application in gait analysis. Two sensors were attached to the toe and heel of the shoes, and two sensors obtained charge output signals by remaining still, sliding back and forth, stepping, and jumping (Figure 10V,VI). The various movements were captured by a designed sensor that showed different waveforms useful for gait recognition.

The flexibility of piezoelectric materials enables customizable smart fabrics that meet user needs.^[^ ^297^ ^]^ Musaddiq et al.^[^ ^298^ ^]^ introduced piezoelectric stretchers to improve tissue surface properties and light distribution during photodynamic therapy (PDT). They addressed uneven tissue surfaces by optimizing structural topology optimization via the piezoelectric effect to stretch tissue precisely. Their research assessed these stretchers via finite element models in a multiphysics context and elucidated that increasing the electric potential improved the laser irradiance uniformity. The surface uniformity of the stretcher improved by 31% from nonexpansion to full expansion, improving laser delivery during PDT. Electrically induced piezoelectric tissue expansion can be used in therapeutic applications because it produces a uniform tissue surface and increases the degree of laser irradiation. The main challenges in developing smart fabrics are the durability of piezoelectric materials during washing and wear and the ability to maintain comfort and breathability. Future developments should focus on enhancing the resilience of these materials while ensuring high performance.^[^ ^299^ ^]^ Chen et al.^[^ ^300^ ^]^ investigated the influence of graphene oxide and reduced graphene oxide on the β‐phase of PVDF. They developed piezoelectric hybrid nanofibers capable of monitoring human movement. Polymer‐assisted dispersion enhanced the β‐phase of reduced graphene oxide (rGO), resulting in an elevated output voltage. Dispersants reduced rGO aggregation, improved stability, and elevated the β‐phase content.

**Figure 11:**
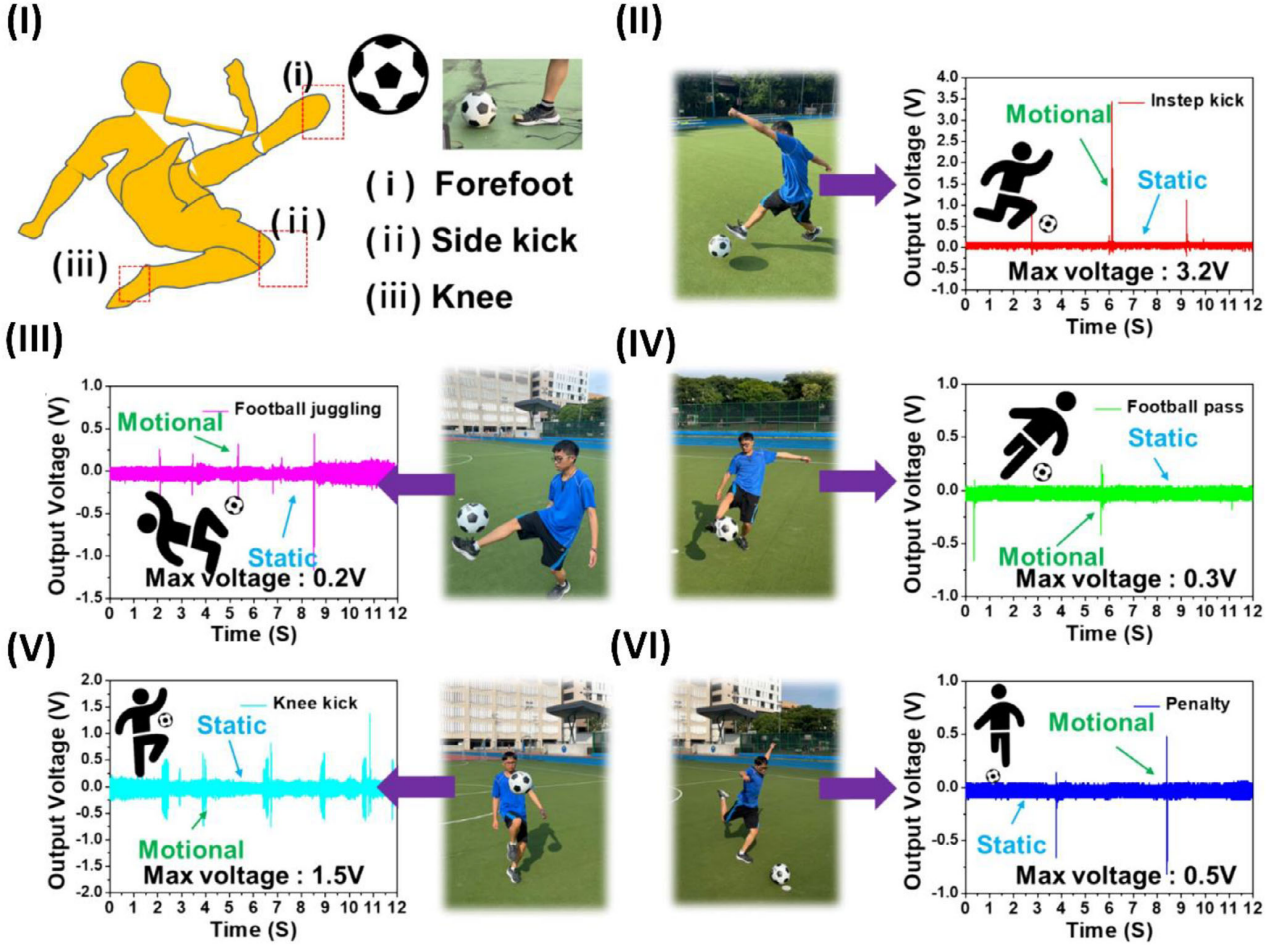
Application of electrospun TX‐100/rGO/PVDF piezoelectric hybrid nanofibers in football tracking I) Schematic illustration of the pulse voltage produced by electrospun TX‐100/rGO/PVDF piezoelectric hybrid nanofibers subjected to varying impacts on different areas of the foot: the front foot, the side foot, and the knee. Piezoelectric hybrid nanofiber output voltages during various football movements, such as II) instep kicks, III) juggling, IV) passing, V) knee kicking, and VI) penalty kicks. Reproduced with the permission.^[^ ^300^ ^]^ Copyright 2024, Elsevier.

The electrospun Triton X‐100 TX‐100/rGO/PVDF piezoelectric hybrid nanofibers exhibited flexibility, exceptional hydrophobicity, and self‐powering capability, making them suitable for football position sensors. Figure 11I illustrates the effects of football kicks—forefoot, sidefoot, and knee—on the performance of a shoe electrospun with TX‐100/rGO/PVDF piezoelectric hybrid nanofibers. Moreover, **Figure** **11**II–VI illustrates that nanofibers can be used to differentiate football kicks, including forefoot, sidefoot, knee, and penalty kicks. The designed nanofiber‐based piezoelectric sensor can distinguish between passing and juggling a ball. The self‐powered sensors enabled the assessment of gait, prevention of injuries, adjustment of posture, and improvement of technology. Compared with other piezoelectric materials, the electrospun TX‐100/rGO/PVDF hybrid nanofibers exhibited significantly enhanced pressure sensitivity, demonstrating superior sensitivity in specific applications.

#### 7.1.3 Energy Harvesting Devices

Energy harvesting devices convert ambient mechanical energy from human activities into electrical energy, providing a sustainable power source for wearable electronics. Piezoelectric materials are at the forefront of this technology.^[^ ^301^ ^]^ Piezoelectric materials can harness energy from everyday movements, such as walking or running. When these materials are deformed, they generate electrical energy that can be stored and used to power wearable devices, reducing their reliance on batteries.^[^ ^302^ ^]^

**Figure 12:**
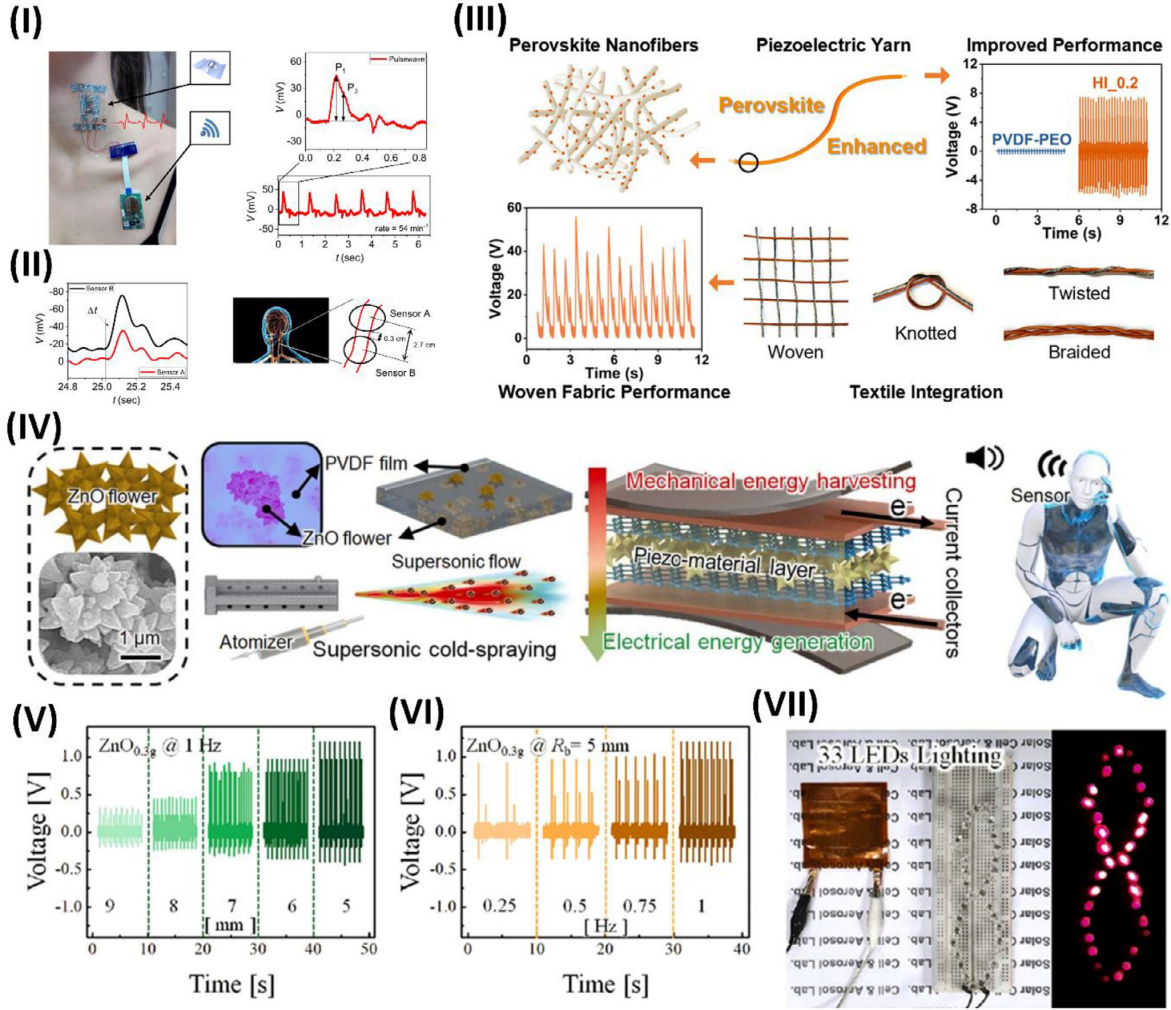
Application of Piezoelectric Energy Harvesting Devices for Health Monitoring. The wireless e‐health patch's attachment locations feature an ultraflexible transducer, an invisible sensor adhering to the skin without adhesive. I) The patch quantifies human pulse wave characteristics, specifically the P1 and P2 peaks. II) Assessment of neck blood pressure through pulse wave velocity (PWV), III) wearable piezoelectric nanogenerator yarns made of nanofibers decorated with cesium lead halide perovskites, IV) supersonic spray deposition of PVDF and ZnO structures featuring integrated nanocuboids for the development of wearable piezoelectric nanogenerators, bending of V) piezoelectric sensors at 5 mm, VI) PVDF/ZnO_0.3g_ at various bending radii, and VII) instantaneous lightning of 33 LEDs by tapping a single PENG. I, II) Reproduced with permission.^[^ ^305^ ^]^ Copyright 2021, Springer. III) Reproduced with permission.^[^ ^306^ ^]^ Copyright 2023, American Chemical Society. IV–VII) Reproduced with permission.^[^ ^307^ ^]^ Copyright 2023, Elsevier.

Energy harvesting from piezoelectric materials can power wearable devices, including health monitors, fitness trackers, and smartwatches. The integration of energy harvesting capabilities allows these devices to operate continuously without frequent battery replacements, increasing user convenience.^[^ ^303^ ^]^ Zhang et al.^[^ ^304^ ^]^ reported the expanding use of smart electronics in wearable electronics, portable tools, exoskeletons, and implantable medical devices. To power body‐attached devices, researchers are exploring sustainable alternatives to standard power sources owing to the rise in energy needs. Their work described collecting mechanical energy from human motion via physical processes, materials, buildings, and design ideas. The development of conformable and wearable electronics is crucial for applications in healthcare, robotics, and cyber‐physical systems. Petritz et al.^[^ ^305^ ^]^ investigated the organic diodes and ferroelectric polymer transducers essential to covert sensing and energy harvesting systems. Ultraflexible ferroelectric polymer transducers create discrete wireless e‐health patches that accurately measure blood pressure and pulse rates. An energy harvesting device was developed by combining transducers and rectifiers that harness biomechanical energy via ultraflexible organic diodes, resulting in a thickness of 2.5 µm and a peak power density of 3 mW cm^−3^. The wireless patch comprised a compact, lightweight wireless module weighing ≈5.6 g and a neck‐mounted sensor that weighed ≈2 mg, excluding connections. It adheres to the subcollar tissue **Figure** **12**I. The designed e‐patch band monitors heart rate, respiration, and pulse. Moreover, two ultraflexible ferroelectric sensors placed on the neck of a 34‐year‐old male recorded pulse signals, as illustrated in Figure 12II.

Piezoelectric nanogenerators (PENGs) convert the mechanical energy generated by body movement into electrical energy. Wu et al.^[^ ^306^ ^]^ introduced a flexible piezoelectric yarn fabricated through a one‐step concentric cesium lead halide perovskite for the PVDF deposition of nanofibers, Figure 12III, which are affixed to stainless steel yarn. In situ electrospinning facilitated the production of perovskite crystals. Devices constructed from a single bundle of CsPbI_2_Br integrated with PVDF nanofibers generate an output voltage of 8.3 V and a current of 1.91 µA upon actuation, facilitating the charging of capacitors for electronic applications. Piezoelectric yarns can be manipulated through bending, twisting, braiding, and weaving for textile applications, enabling energy collection from body movements and facilitating wearable mechanical energy harvesting. Joshi et al.^[^ ^307^ ^]^ used supersonic spraying of the piezopolymer polyvinylidene fluoride (PVDF) over hydrothermally synthesized ZnO flowers, including nanocuboids, to fabricate ZnO‐embedded PVDF films for flexible PENG devices, as highlighted in Figure 12IV.

Through substantial supersonic impact and shear flow, ZnO facilitates the transformation of PVDF from its nonpolar α‐phase to its polar β‐phase. Compared with that of pure PVDF before coating, the β‐phase in the PVDF/ZnO composite sprayed with ultrasonic technology increased by 32%. At an open‐circuit voltage of 1.2 V, the piezoelectric coefficient for the pure PVDF films was 43 pm V^−1^, whereas for the PVDF/ZnO composite films, it was 150 pm V^−1^. The substantial role of ZnO in enhancing the β‐phase in PVDF during supersonic spraying is shown by marked fluctuations. The monitoring of biological signals, tactile sensing, and the development of self‐powered wearable sensors have garnered considerable interest. Therefore, a bending test was performed on the PVDF sensor, and insufficient piezoelectricity was produced due to mild strain. However, when ZnO was introduced into the PVDF matrix, the response was visible when the piezoelectric potential reached 1.2 V, which validated the stress transfer capability of the designed piezoelectric material (Figure 12V). However, the output voltage remained lower at a larger radius because less stress was exerted on the films (Figure 12VI). Likewise, a PVDF/ZnO_0.3_ PENG sample was sufficient to produce a voltage that powered 33 LEDs when connected in series (Figure 12VII), thus validating piezoelectricity production by harvesting different forms of mechanical energy.

Su et al.^[^ ^308^ ^]^ presented a self‐powered piezoelectric nanogenerator (PENG) sensor, which showed considerable effectiveness against bacteria and possessed superhydrophobic characteristics. The PENG sensor comprises a piezoelectric nanofiber membrane electrospun from poly(vinylidene fluoride‐co‐tetrafluoroethylene). Each fiber was subsequently enveloped in a crosslinked conformal hydrophobic nanocoating, which was applied through initiated chemical vapor deposition (i‐CVD). The acquired sensor exhibited dependable recording capabilities for various human actions, including breathing and movements. The PENG sensor, recognized for its remarkable long‐term stability and durability, was incorporated into shoe insoles to enable human gait tracking. Narendra et al.^[^ ^309^ ^]^ presented a biocompatible silk hydrogel integrated with ZnO nanorods (ZnONRs), which can adhere to the skin for triboelectric and piezoelectric energy generation. Compared with the unmodified silk hydrogel, the integration of ZnONRs into the silk hydrogel resulted in an eightfold increase in piezoelectricity, which doubled the enhancement when ZnONRs were encapsulated within the silk protein layers. The EG‐skin generated ≈1 mW cm^−2^ of energy, sufficient to power low‐energy devices such as LEDs and oximeters.

Additionally, it can detect finger movements and generate immediate electrical impulses as a tactile sensor. Owing to its flexibility and adhesive properties, the hydrogel adhered to human skin and absorbed mechanical energy (6.2 and 0.9 µW cm^−2^). The EG‐skin, made from silk protein, can improve biomedical implants, soft robots, human‒machine interfaces, and tangible sensors. The adaptable biodegradable piezoelectric composite films, characterized by self‐powered, biocompatible, and biodegradable properties, were created by integrating water‐soluble stiff piezoelectric crystals with flexible polymers. These films exhibited considerable potential for applications in implantable and wearable bioelectronics. Despite advancements, fabricating flexible films with high piezoelectric coefficients remains challenging.

### 7.2 Implantable Electronics

The rapid advancement of implanted electronic technologies has enhanced everyday living and smart healthcare. Nevertheless, implantable, durable electronics contribute to pollution via hazardous waste. Therefore, transient electronics, which disintegrate or deteriorate over time, provide an eco‐friendly alternative via transitory devices. These materials may be used in biomedical applications without producing detrimental residues or necessitating surgical removal.^[^ ^335^ ^]^ Therefore, this section focuses on applying piezoelectric materials in advancing transient and implantable electronics. A summarized overview of implanted piezo applications is listed in **Table** **6**.

**Table 6:** Summary overview of piezoelectric applications in implantable devices.

Device	Piezoelectric Material	Doping/Enhancement Material	Mechanism	Fabrication Method	Performance	Application	Reference
US‐PENG	P(VDF‐CTFE)	BaTiO₃@Carbon (BT@Carbon)	Ultrasound‐induced piezoelectric effect	Nanocomposite synthesis	521 µW cm^−2^	Wireless power for implants	[353]
T‐SNR Film	Silk nanoribbons	None	Electrostriction without poling	Oxidation treatment	d₃₃ = 7.7 pm V^−1^	Biodegradable sensors	[354]
PBPS	PLLA	BTO nanoparticles	Motion‐to‐electric conversion	Electrospinning	Linearity ≈ 0.9445	Nerve recovery monitoring	[355]
NFPM	Unspecified	None	Ossicular vibration detection	Laser welding	Effective detection	Middle‐ear microphone	[356]
Vascular System	PVDF nanofibers	None	Hemodynamic sensing	Growable sheath	Sensitive, adaptive	Blood flow monitoring	[357]
F‐SS EG Device	Silk sericin	None	Mechanical energy harvesting	Silk functionalization	d₃₃ = 12 pCN^−1^, 218.5 µW m^−2^	Biodegradable CIEDs	[358]
Flexible Harvester	PIN–PMN–PT	Ni stressor, MIM	Heartbeat energy harvesting	Crystal film fabrication	20 µA, 3.08 µA mm^−3^	Cardiac sensors	[359]
Acoustic Transducer	Unspecified	None	Sound filtering (250–6000 Hz)	Surgery‐compatible design	320.3 mVpp, SNR 84.2 dB	Cochlear implants	[360]
Glycine‐PEO Film	γ‐glycine	PEO	Interface‐enhanced piezoelectricity	PTFE interface evaporation	d₃₃ ≈ 8.2 pCN^−1^	Wearable/implantable electronics	[337]
Antibacterial Patch	PZT	None	Ultrasound‐triggered SDT/PDT	Wireless ultrasound control	Complete abscess cure	Deep infection treatment	[361]
Gly‐Nb2C‐NFs Sensor	β‐glycine	Nb₂CTx nanosheets	Interfacial polarization locking	Self‐assembly	d₃₃ = 5.0 pCN^−1^, voltage coefficient = 129 × 10⁻^3^ V·m N^−1^	Microvibration sensing in vivo	[362]
OsUTR	Unspecified	–	Ultrasound wireless power transfer	Oblong‐shaped ultrasound transmitter and receiver	246.93 mW cm^−2^, 1.64 mC s^−1^ charging rate	Efficient wireless power for IBEs	[363]
PZP Scaffolds	PVDF	ZnO, PCL	Exercise‐driven electrical stimulation	3D printing and rolling techniques	Promotes cartilage regeneration, good biocompatibility	Osteoarthritis treatment via cartilage regeneration	[364]
Full‐Custom FICI System	Unspecified (likely PZT or similar)	None	Sound sensing and electrical stimulation via MEMS cantilever beams	MEMS‐based acoustic sensor with bilayer piezoelectric design	High SNR, effective auditory nerve stimulation (eABR observed at 45–100 dB SPL)	Fully implantable cochlear implants	[365]
PVDF Antibacterial Interface	PVDF	Antibacterial essential oil nanoparticles and antibiofilm enzymes	Bone integration and antibacterial action via piezoelectric stimulation	Layer‐by‐layer (LBL) coating on PVDF films	Enhanced preosteoblast proliferation, antibiofilm activity against *P. aeruginosa* and *S. aureus*	Implantable devices with improved osseointegration and infection resistance	[366]
MEMS‐based PUEH	PZT‐5H	None	Ultrasonic energy harvesting	Low‐temp MEMS fabrication with epoxy bonding	0.62 V RMS, 0.19 mW RMS at 200 kHz, 20 mm range	Powering miniaturized implantable medical devices	[367]
FB‐HNG (Fully Biodegradable Hybrid Nanogenerator)	PVA/Gy/PVA	PLGA (triboelectric layer)	Piezo‐triboelectric hybrid energy harvesting	Electric field‐assisted water evaporation	94 V, 2.3 µA, 1.53 µA cm^−2^, 6.53 nC cm^−2^	Biodegradable power source for transient medical devices	[368]
AlScN PMUT‐based UWPT Receiver	Al90.4%Sc9.6%N	Acoustic matching gel	Ultrasonic wireless power transfer	PMUT array (13 × 13), CMOS‐compatible	2.33% PTE, 3.3 V DC output	Miniaturized wireless charging for IMDs	[369]
ULFMEF (Ultra‐Low Frequency Magnetic Energy Focusing)	Magnetic core (nonpiezoelectric)	None	Rotating magnetic field for deep‐tissue wireless power	Pellet‐like implant with internal coils	4–15 mW across 20 cm tissue, no heat	Deep‐tissue wireless powering for optogenetics and stimulation	[370]
PLLA/Gly Nanofiber Generator	PLLA (core)/Glycine (shell)	Interfacial hydrogen bonding (─OH and C═O)	Stabilized β‐phase piezoelectricity	Electrospinning of core/shell nanofibers	High β‐phase content, enhanced piezoelectricity	Flexible nanogenerators for physiological motion sensing	[346]
Lead‐Free Cruciform Piezoelectric Array	(K,Na)NbO₃‐based ceramic/polymer composite	Tuned 1–3 composite ratio	Ultrasonic wireless energy harvesting	Cruciform flexible array with serpentine electrodes	22.75 Vpp, 57.1 µW average charging rate	Wireless powering of implantable bioelectronics	[371]
Ultrasound‐Driven BZT‐BCT/PVDF Neurostimulator	BZT‐BCT nanowires + PVDF	–	Ultrasound‐programmable electrical stimulation	Thin‐film composite fabrication (≈30 µm)	Direct neurostimulation via ultrasound pulses	Battery‐free, programmable peripheral nerve stimulation	[372]
Kirigami‐Inspired Pacemaker Lead Harvester	Piezoelectric composite film	Kirigami patterning	Biomechanical energy harvesting from heart motion	Integrated into the pacemaker lead, FEM‐optimized	2.4 µW power, ≈0.7 V in vivo	Self‐powered pacemaker leads	[373]
Titanium Piezo‐Microfluidic Platform	Piezoelectric actuator	–	Fluid actuation (pumping, valving)	Titanium‐based MEMS with shared actuator design	14 ± 2.2 mL/min flow, 75 kPa pressure, <1 µL min^−1^ leakage	Automated fluid control in implantable devices	[342]
TPSS (Tissue‐Adhesive Piezoelectric Soft Sensor)	Piezoelectric soft sensor (material unspecified)	Adhesive hydrogel (AH)	Biomechanical‐to‐electrical signal conversion with enhanced tissue adhesion	Integration of a piezoelectric sensor with tissue‐adhesive hydrogel	8.3 V output, 186.9 µW m^−2^ power density, >6000 cycles stability	Seamless, battery‐free blood pressure monitoring in vivo (e.g., carotid artery)	[374]

#### 7.2.1 Biodegradable Piezoelectric Monitoring Devices

**Figure 13:**
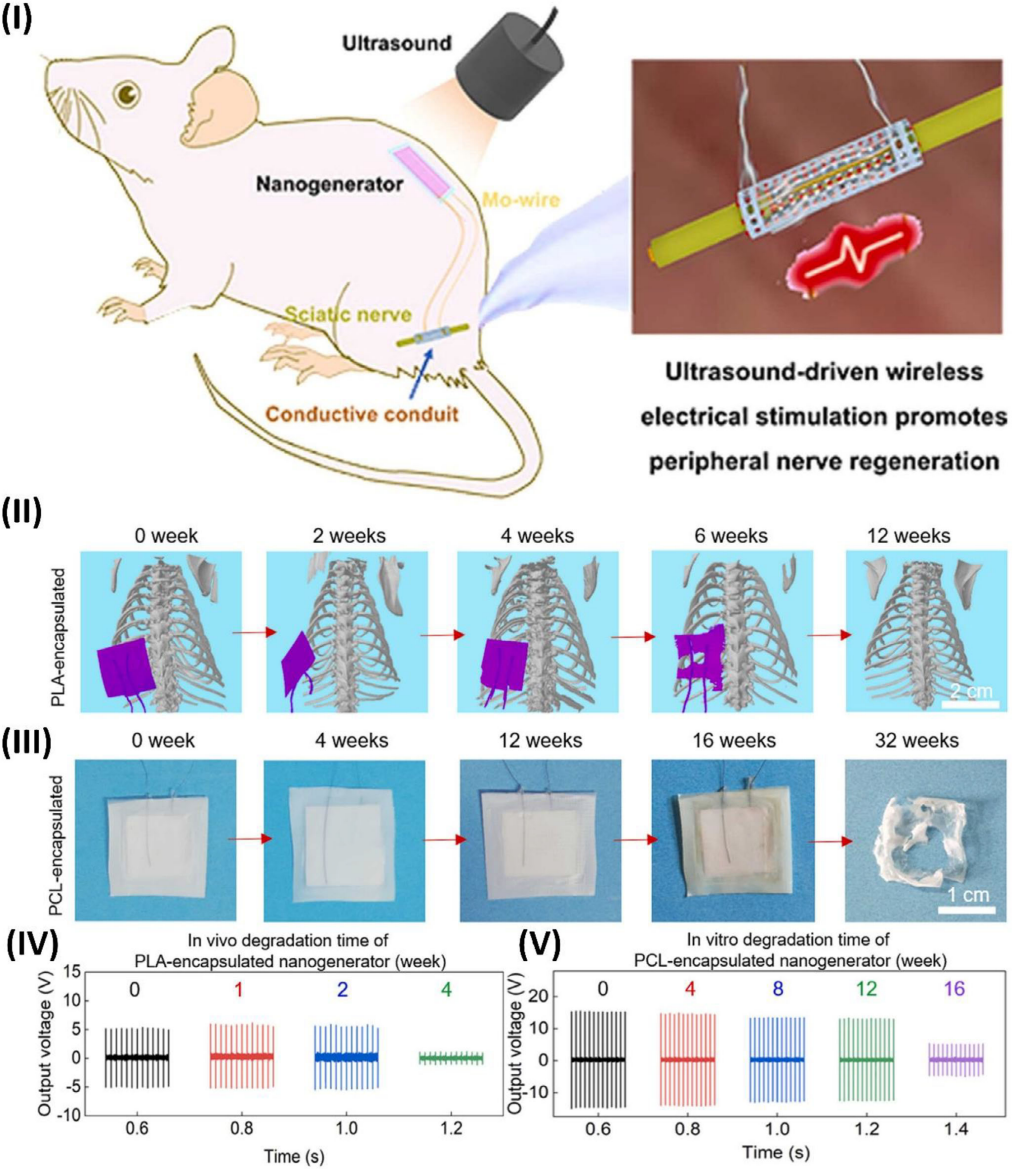
Applications of biodegradable piezoelectric monitoring devices. I) Biodegradable piezoelectric nanogenerator for ultrasound‐driven in vivo simulation for monitoring and enhancing nerve tissue repair; II) PLA‐encapsulated PHBV/PLLA/KNN‐based nanogenerator micro‐CT images obtained at various stages; III) biodegradation performance of the nanogenerator for 32 weeks in a phosphate buffer saline solution; IV) output voltage of the in vivo‐implanted PLA‐encapsulated nanogenerator obtained at various times; V) time‐dependent output voltages of the nanogenerator immersed in PBS. Reproduced with permission.^[^ ^336^ ^]^ Copyright 2022, Elsevier.

Transient electronics can be used for short‐term health monitoring applications, such as postoperative patient monitoring or temporary health assessments. Piezoelectric materials enable these devices to collect vital data during their intended lifespan. Wu et al.^[^ ^336^ ^]^ demonstrated the use of an ultrasound‐driven biodegradable piezoelectric nanogenerator (PENG) to enhance peripheral nerve healing. They utilized biodegradable substances such as KNN nanowires, PLLA, and PHBV to provide in vivo electrical stimulation (ES) upon activation by ultrasound. ES facilitated nerve regeneration and enabled real‐time observation of nerve repair via muscle electrophysiological responses. The PENG system, which includes biodegradable encapsulating layers and electrodes, produces customizable ES parameters via ultrasonic pulses. Experiments on rats demonstrated that the ES group had superior nerve regeneration compared with the non‐ES group, achieving results comparable to those of the autograft group, the benchmark for nerve repair. This technique allows sustained postoperative electrical stimulation administration and real‐time monitoring of tissue recovery, hence minimizing the need for intrusive treatments, as illustrated in **Figure** **13**I. Their research indicated that ultrasound‐mediated wireless electrostimulation using biodegradable piezoelectric nanogenerators may substantially improve and assess brain and other types of tissue regeneration, indicating interesting therapeutic applications.

Moreover, the biodegradation process of PLA was engineered by adjusting the thickness of the PLA. They used PLDs with a higher molecular weight of up to 20 K for device stability. They tested the in vivo stability and degradation by implanting PHBV/PLL/KNN nanogenerators and encapsulating them with a 200 µm PLA layer. The rats were sacrificed for stability, and the output current and voltage of the implanted piezoelectric nanogenerator were measured. The micro‐CT images indicated that the nanogenerator and molybdenum wire electrodes completed their morphology at week 2, as shown in Figure 13II, with no obvious decrease in the output voltage Figure 13IV. Furthermore, the micro‐CT image indicated that the implanted nanogenerator had a complete morphology, but the output voltage was reduced at week 4. However, biodegradation was observed at week 6, and the implanted nanogenerator was not found at week 12, validating the biodegradability of the encapsulated nanogenerator. Likewise, degradation behavior was also observed in PBS at 37 °C. The nanogenerator maintained decent structural integrity during the first 12 weeks, as illustrated in Figure 13III. However, the performance of the nanogenerators decreased gradually, but the output performance retained 80% of its initial status up to week 12, as highlighted in Figure 13V, indicating the feasibility of long‐term in vivo ES.

Yu et al.^[^ ^337^ ^]^ investigated the types, arrangements, structures, and piezoelectric properties of γ‐glycine, a fundamental natural biodegradable piezoelectric crystal, along with the effects of different water‐soluble, biodegradable polymers and film‐forming interfaces. A hydrophobic PTFE interface facilitated the formation of the glycine‒PEO composite film. The film exhibited an out‐of‐plane piezoelectric coefficient (d_33_) of approximately 8.2 pCN^−1^, closely aligned with the predicted d_33_ of 10.4 pCN^−1^ for γ‐glycine. The results offered insights into how interfaces affect composite films' piezoelectric properties and crystal structure, facilitating further research into the flexible production of piezoelectric materials. Moreover, Ensieh et al.^[^ ^338^ ^]^ developed a biodegradable, flexible pressure sensor utilizing glycine‐chitosan piezoelectric sheets. Stable β‐glycine spherulites are produced by films formed through the self‐assembly of glycine in a chitosan solution. The dielectric properties of the films were outstanding, with a sensitivity comparable to that of commercial materials (≈2.82 ± 0.2 mV kPa^−1^). The glycine‐to‐chitosan ratio influenced the crystallization and morphology of the spherulites. Higher ratios resulted in fibrils characterized by increased voids and accelerated nucleation, whereas lower ratios yielded fibrils with reduced voids and slower nucleation rates. Chitosan plays a role in polymorph selectivity, as evidenced by the absence of glycine crystals. Their study highlighted the potential of glycine‒chitosan composites for use as biodegradable sensors in wearable biomedical diagnostics. Recent advancements in neuromodulation underscore the promise of ultrasound‐based approaches to address the shortcomings of electrode‐based stimulation, including immunological response and inadequate spatial accuracy.^[^ ^339^ ^]^ Therefore, F. Hou et al.^[^ ^340^ ^]^ reported the ImPULS device, a fully implantable, flexible piezoelectric micromachined ultrasound transducer that employs biocompatible potassium sodium niobate [(K, Na)NbO₃] to provide focused ultrasonic pressure (≈100 kPa) for neuronal stimulation. Unlike conventional systems, ImPULS eliminates electrochemically active components, hence improving long‐term stability. This platform provides a nongenetic, spatially resolved methodology for deep brain stimulation and functional neuroscience investigations.

Wang et al.^[^ ^341^ ^]^ reported a piezoelectric bone cement consisting of poly(methyl methacrylate) (PMMA) and 15 wt% barium titanate (BaTiO₃) to address aseptic loosening in orthopedic implants. This composite demonstrates significant electrobioactivity, producing an open‐circuit voltage of 37.1 V under biomimetic mechanical stress. In vitro investigations revealed a significant increase in the expression of osteogenic markers in response to cyclic stimulation. The cement increased bone development by more than 4.6 times in both static and dynamic situations two months after implantation. Electrical stimulation activates calcium‐sensitive receptors in bone marrow stromal cells (BMSCs), resulting in a 1.41× increase in calcium ion influx and facilitating osteogenic differentiation. The results highlight the potential of PMMA–BaTiO₃ cement as a bioactive, mechanically sensitive substance for enhancing implant integration in load‐bearing orthopedic applications. A titanium‐based piezoelectric microfluidic platform was developed by Bußmann et al.^[^ ^342^ ^]^ to fulfill the requirements for small, safe, and imaging‐compatible fluidic systems in medical implants; this platform consists of a diaphragm pump, a usually closed valve, and a normally open valve. All devices possess a cohesive actuation mechanism and design, facilitating seamless system integration. The use of titanium promises biocompatibility, hermetic sealing, and reduced imaging artifacts. The diaphragm pump attained a maximum flow rate of 14 ± 2.2 mL min^−1^ and a pressure increase of 75 kPa. The usually closed valve exhibited outstanding sealing capabilities, with leakage rates of 1 µL min^−1^. Thorough evaluations include actuator stroke analysis, valve longevity testing, and both computational and experimental analyses of valve mechanics. This platform provides a robust basis for the creation of smart, energy‐efficient, and compact implanted devices for sophisticated medical treatments.

#### 7.2.2 Smart Fabrics

Transient implantable smart fabrics can be designed for single‐use applications, such as monitoring during specific events such as athletic competitions or medical procedures. These fabrics can incorporate piezoelectric materials to monitor physiological parameters and then degrade after their intended use.^[^ ^343^ ^]^

**Figure 14:**
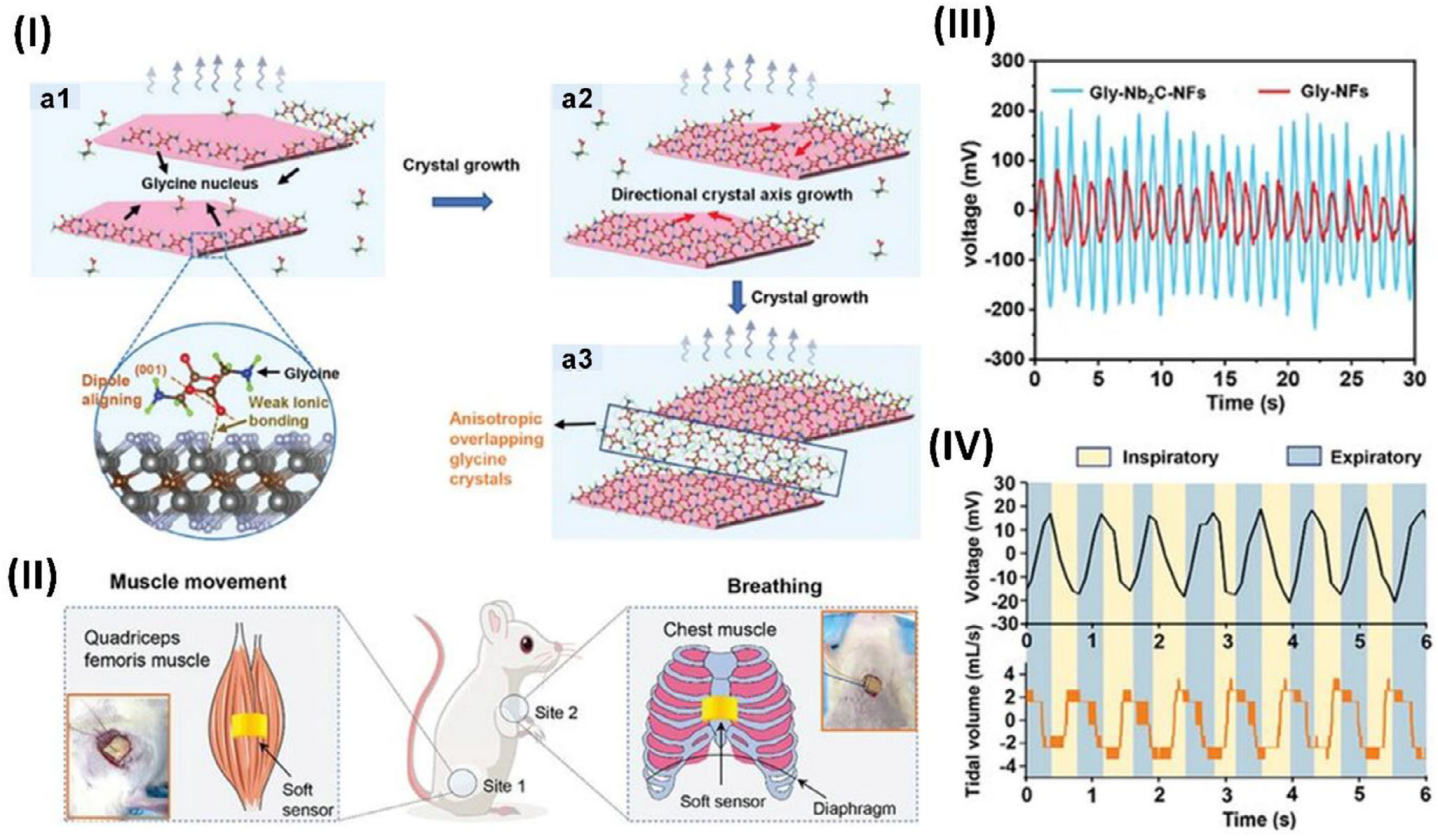
Mechanisms of synthesis and growth of piezoelectric Gly‐Nb_2_C‐NFs. I) Schematic representation of the dipping and pulling process of Gly‐Nb_2_C‐NFs. The inset shows digital images of the as‐received, predominantly curved thin film and its folding, demonstrating significant flexibility. The mechanism of glycine molecule growth on Nb_2_CT_x_ nanosheets. a1) Glycine crystals nucleate at the edges of Nb_2_CT_x_ nanosheets. a2) Glycine crystals exhibited directional growth on the surfaces of the Nb_2_CT_x_ nanosheets. a3) Glycine crystals developed in the gaps between the Nb_2_CT_x_ nanosheets on the PAN nanofibers. The inset shows the interaction between glycine and Nb_2_CT_x_ nanosheets. II) Schematic images and digital photographs depicting the implantation of Gly‐Nb_2_C‐NFs in the thigh and chest regions of rats. III) Piezoelectric voltage outputs of Gly‐Nb_2_C‐NFs and Gly‐NFs implanted on the quadriceps femoris muscle in the thigh region during gentle stretching. IV) Piezoelectric voltage outputs from the Gly‐Nb_2_C‐NFs induced by respiration when implanted on the pectoralis major muscle in the chest, along with the corresponding respiratory curve. Reproduced with permission.^[^ ^344^ ^]^ Copyright 2024, Wiley.

Zheng et al.^[^ ^344^ ^]^ proposed a nanoconfinement self‐assembly method for the large‐scale production of glycine and Nb_2_CT_x_ nanofibers (Gly‐Nb_2_C‐NFs). Briefly, Nb_2_CT_x_ sheets were used as a nucleating agent to induce glycine crystallization. Weak ion bonds developed between the Nb atoms of Nb_2_CT_x_ and the oxygen atoms of glycine, which further induced the directional crystal axis growth of 𝛽‐glycine. The nanofibers exhibited unique interfacial polarization, stabilizing aligned crystal domains **Figure** **14**I. This concept facilitated the development of piezoelectric biomolecular materials influenced by two‐dimensional materials by offering an effective mechanism for systematically controlling crystallization in glycine crystals. The developed Gly‐Nb_2_C‐NF piezoelectric sensor was employed for in vivo microvibration monitoring in mice. The designed piezoelectric sensor devices with a size of 8/8 mm were implanted under the chest and thigh skin of adult rats, where considerable biochemical energy was possible, as illustrated in Figure 14II. When the leg was stretched, the embedded device produced a consistent voltage of >300 from the Gly‐Nb_2_C‐NFs, in contrast to ≈100 mV from the Gly‐NFs, as depicted in Figure 14III. Moreover, the device attached to the pectoralis major muscle produced a voltage of >20 mV during rat respiration Figure 14IV. The generated voltage was comparable to that reported for other piezoelectric sensors.

A biodegradable piezoelectric nanofiber film consisting of glycine crystals embedded in polycaprolactone (PCL) was developed by T. Chorsi et al.^[^ ^345^ ^]^ to address the brittleness and solubility challenges associated with glycine‐based materials. The glycine–PCL composite demonstrates stable piezoelectric performance, producing a high ultrasound output of 334 kPa at 0.15 Vrms, exceeding that of current biodegradable transducers. An entirely biodegradable ultrasonic transducer (5 mm × 16 mm × 330 µm) was developed utilizing this film, incorporating molybdenum (Mo) electrodes and polylactic acid (PLA) encapsulation. The device, which was implanted in mice, facilitated repeated and localized opening of the blood‒brain barrier through ultrasound‐mediated microbubble cavitation, thereby improving the delivery of chemotherapeutic agents to the brain. Immunofluorescence imaging demonstrated significant dextran penetration in both superficial and deep brain regions after sonication with the glycine–PCL transducer, surpassing the performance of bare PCL and piezoelectric PLLA controls. This resulted in enhanced blood‒brain barrier disruption and improved drug delivery efficiency. The operational lifetime of the device was adjustable (10–25 days) through modifications in the PLA encapsulation thickness, with complete degradation verified under physiological conditions. This eliminates the need for surgical intervention, providing a secure and efficient framework for temporary brain therapies.

**Figure 15:**
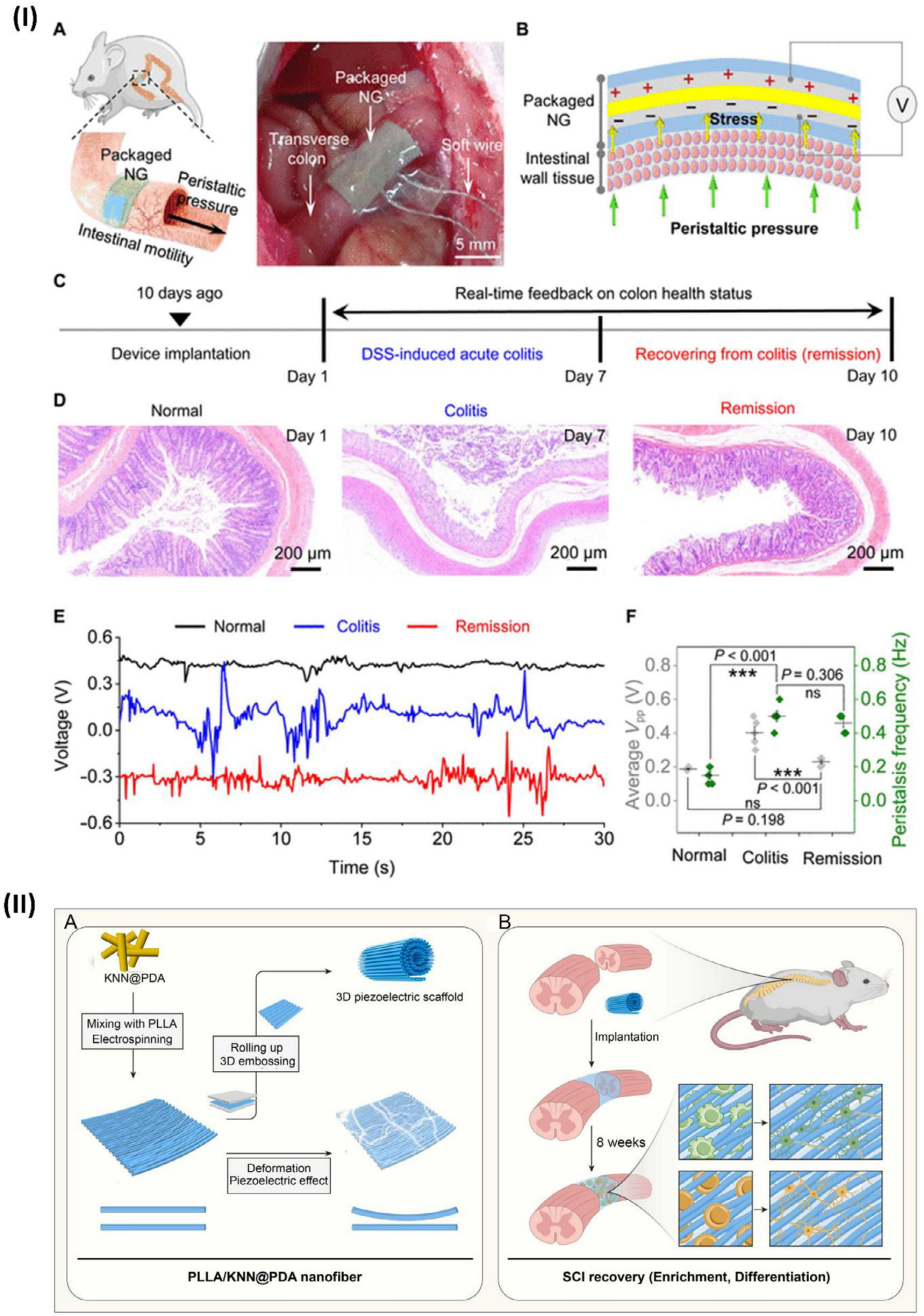
Smart implantable transient piezoelectric fabric bioengineering applications. I) In vivo evaluation of core/shell PLLA/glycine nanofiber‐based nanogenerators. A) The in vivo functionality of the PLLA/Gly NF‐based nanogenerator (NG) was demonstrated through implantation in the transverse colon of rats to monitor intestinal peristalsis. B) The device, connected via soft silver‐plated PTFE Teflon wires, conformed tightly to the wrinkled colon surface and effectively converted mechanical motion into electrical signals via the piezoelectric effect. C) To assess diagnostic potential, a DSS‐induced acute colitis model was established. D–E) The NG captured real‐time voltage signals corresponding to different physiological states—normal, inflamed, and remission—correlating with the histological changes observed in H&E‐stained colon tissues reprinted under License 4.0 (CC BY‐NC).^[^ ^346^ ^]^ Copyright 2024, American Association for the Advancement of Science (AAAS). II) Figure 1 Wireless electrical stimulation via piezoelectric PLLA/KNN@PDA scaffolds for spinal cord repair. A) Schematic illustration of the fabrication process for PLLA/KNN@PDA composite fibers, which integrate biodegradable polymers with piezoelectric nanowires to form electroactive scaffolds. B) Application of the piezoelectric scaffold in promoting neural regeneration following SCI, demonstrating its potential for wireless, self‐powered electrical stimulation in regenerative therapies. Reproduced with permission.^[^ ^347^ ^]^ Copyright 2024, Elsevier Ltd.

Poly(L‐lactic acid) (PLLA), an FDA‐approved implantable biomaterial, exhibits intrinsic piezoelectricity but suffers from instability in its β phase and poor domain alignment.^[^ ^19^ ^]^ To overcome these limitations, Li et al.^[^ ^346^ ^]^ employed a unique interfacial anchoring technique to develop core/shell PLLA/glycine (Gly) nanofibers (NFs) by electrospinning, thereby addressing these constraints. The resultant nanofibers exhibited better β‐phase stability and orientation owing to robust intermolecular contacts between Gly and PLLA, resulting in markedly enhanced and stable piezoelectric performance. A flexible nanogenerator (NG) was constructed from these nanofibers and encapsulated in PDMS for in vivo applications, as illustrated in **Figure** **15**I. Upon implantation into the wrinkled surface of the transverse colon Figure 15I(A), the NG adhered securely without separation and efficiently transduced peristaltic motion into electrical impulses Figure 15I(B). The apparatus records intricate motion patterns, including frequency and amplitude, allowing real‐time observation of gastrointestinal function. For diagnostic ability, acute colitis was induced in rats via dextran sulfate sodium (DSS), and the NG effectively monitored illness development and remission via voltage signal patterns, Figure 15I(C–F). Rats with colitis had heightened high‐ and low‐amplitude voltage activity, which returned to baseline after recovery, underscoring the value of the NG in disease diagnosis and therapy assessment. Biocompatibility was validated by histopathological examination. An insignificant immunological response was observed at one week postimplantation, with complete recovery by week five. No pathological alterations were observed in the main organs up to 35 days postimplantation, confirming systemic safety and long‐term stability.

Neural repair presents a significant challenge within the field of regenerative medicine. In that context, Zhang et al.^[^ ^347^ ^]^ reported a novel biodegradable piezoelectric scaffold by integrating organic poly‐L‐lactic acid (PLLA) with polydopamine‐coated potassium sodium niobate nanowires (KNN@PDA) to address this issue. The scaffold is composed of a biodegradable piezoelectric polymer combined with potassium sodium niobate and features a uniform porous 3D structure formed via low‐temperature hot pressing, as illustrated in Figure 15II(A). In vitro studies demonstrated that the scaffold significantly enhanced neurite outgrowth and proliferation of dorsal root ganglion (DRG) neurons and neural stem cells (NSCs). In vivo implantation in a rat SCI model resulted in accelerated neural regeneration and motor function recovery, as shown in Figure 15II(B). The device's biocompatibility and biodegradability eliminate the need for surgical removal, offering a promising strategy for self‐powered, implantable electrotherapy in neural tissue engineering.

#### 7.2.3 Self‐powered Biodegradable Implants and Transducers

Current research efforts are increasing to develop biodegradable piezoelectric nanogenerators, or PENGs, as self‐powered medical implants and independent health monitors. Moreover, ferroelectrics have been used in stretchable sensors, actuators, and microenergy harvesters; nevertheless, they are often composed of nondegradable petroleum‐based resins, which pose recycling difficulties. Ma et al.^[^ ^348^ ^]^ presented biodegradable and bioabsorbable ferroelectric films composed of polylactic acid (PLA) resins for delicate transducer applications. These films were produced in piezoelectric d_33_ or d_31/32_ mode and exhibited considerable longitudinal and transverse piezoelectric activity due to microstructural and polarization alterations. The PLA films had a thickness of 400 µm and an overall density of 350 kg m^−3^, demonstrating Young's moduli between 0.1 and 10 MPa. Following polarization, they attained elevated piezoelectric coefficients (d_33_ reaching 500 pCN^−1^, g_33_ reaching 40 V mN^−1^, d_31_ (d_32_) reaching −44 pCN^−1^, and g_31_ (g_32_) reaching −3.6 V mN^−1)^. The longitudinal piezoelectric coefficients were similar to those of nondegradable polymers; however, the transverse activity was better. The preparation procedure was suitable for large‐scale manufacturing and facilitated their application in green electronics.

Hu et al.^[^ ^349^ ^]^ developed a bifunctional electroacoustic transducer using flexible and biodegradable cellular polylactic acid (PLA) ferroelectric sheets. These films, with low acoustic impedance and high figure of merit, were suitable for use as microphones and loudspeakers. As a microphone, the device has a reception sensitivity of 4.2 mV Pa^−1^ and a signal‐to‐noise ratio of 23.5 dB at 1 kHz. It produces sound pressure levels between 60 and 103 dB across a 1–80 kHz frequency range as a loudspeaker. The device's stable electrical response between 300 and 3000 Hz enabled accurate speech recognition and control. The PLA ferroelectric films are made from modified PLA resin, foamed with CO_2_, and processed to enhance their properties. They degrade rapidly under specific conditions, with over 80% degradation in 10 hours at 100 °C in phosphate‐buffered saline. However, degradation in natural environments is slower and requires further study. This technology offers eco‐friendly AI and Internet of Things (IoT) applications.

**Figure 16:**
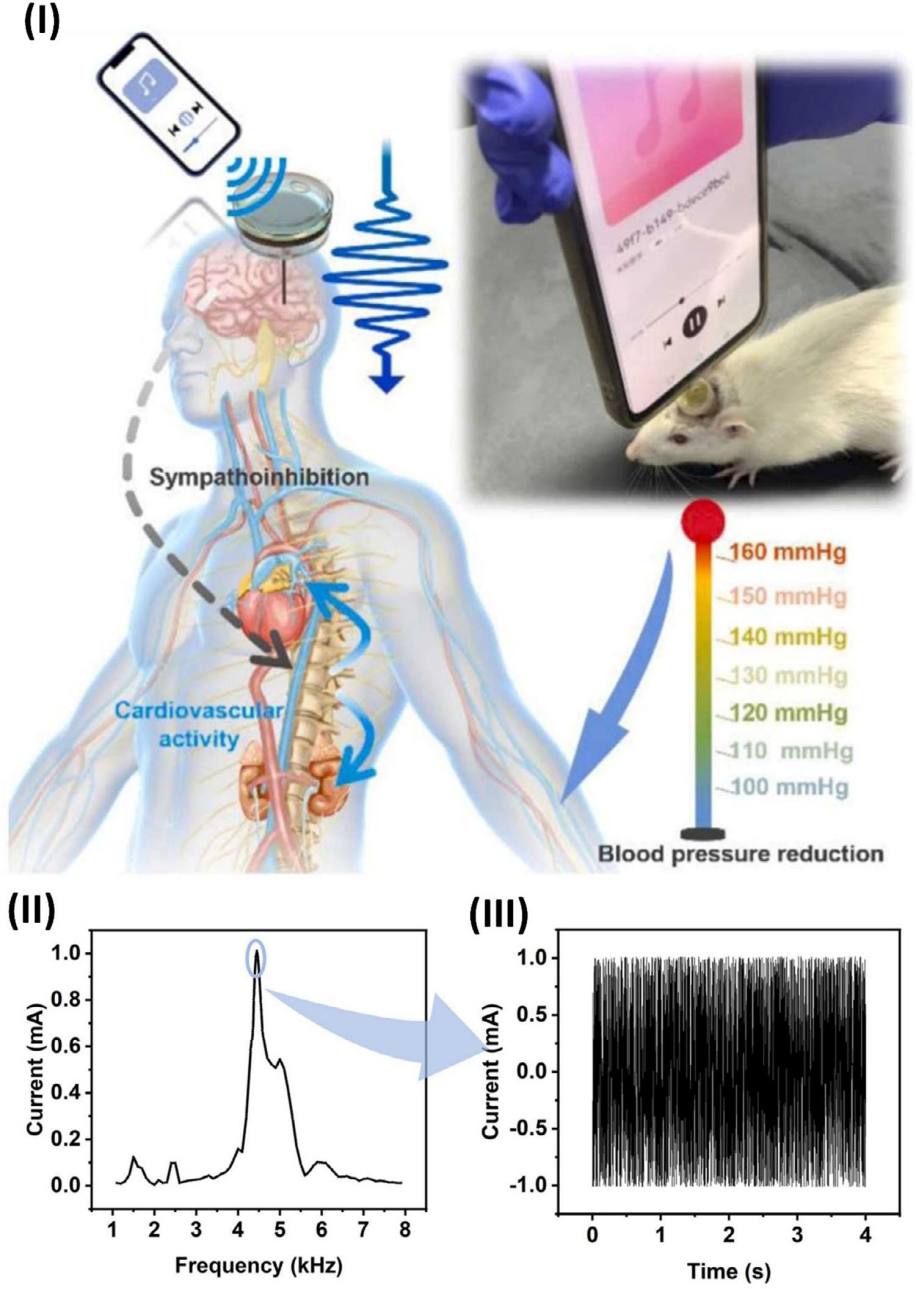
Application of self‐powered biodegradable implants and transducers. I) Self‐powered brain probe for blood pressure regulation; II) the current of the resonator varies from 1000 to 8000 Hz; III) the output current when both the acoustic and resonant frequencies are the same. Reproduced with permission.^[^ ^350^ ^]^ Copyright 2023, Elsevier.

Shan et al.^[^ ^350^ ^]^ designed a novel, biocompatible, self‐powered brain probe as a blood pressure regulator. The device comprises a piezoelectric transducer, a brain‐stimulating module, an electronic module, and a drug microneedle array. The piezoelectric transducer was embedded in a resonant structure, enabling acoustic energy harvesting from smartphone audio tones. They programmed audio signals and activated the resonator, and the electronic module produced an output signal corresponding to the electrostimulation signals. Brain‐simulating implanted electrodes in rat ventrolateral periaqueductal gray matter (vlPAG) are wirelessly controlled by a smartphone, resulting in a reduction in blood pressure to 20 mmHg, as shown in **Figure** **16**I. This novel self‐powered treatment has shown great potential for hypertension treatment and brain‒machine interfaces. The acoustic energy harvester obtained the output current when the frequency varied from 1 to 8 kHz, and the sound pressure was 126 dB, as illustrated in Figure 16II. The distance between the resonator and the sound source was 1 mm, and the angle between them was set at 0°. The resonator with a resonant frequency of 4450 Hz has an output current of 1.012 mA, as demonstrated in Figure 16III. These multifunctional, self‐powered piezoelectric systems are expected to lead to significant developments in wearable and implantable systems.

Implanted nanogenerators (NGs) provide a feasible alternative for self‐sustaining power sources in cardiovascular implanted electronic devices, such as pacemakers. Traditional piezoelectric nanogenerators have a low energy conversion efficiency, which has been a serious challenge.^[^ ^351^ ^]^ To address this problem, Wang et al.^[^ ^352^ ^]^ reported an instantaneous piezoelectric nanogenerator (i‐PENG), which can convert the wave‐like output of traditional PENGs into high‐amplitude electrical spikes, resulting in an approximately seven times larger output and much faster capacitor charging. The i‐PENG is a compact, wirelessly powered device made up of a piezoelectric PVDF sheet, isolated contact electrodes, a rectifier, and a microcapacitor. When fastened to the heart surface of the swine, the device charged a 100 µF capacitor to 4.6 V in approximately 13 minutes, which was adequate for activating a commercial pacemaker and providing continual cardiac stimulation, as validated by ECG monitoring. This concept demonstrates considerable improvement in the efficiency of biomechanical energy harvesting, allowing for the creation of battery‐free, wire‐free pacemakers. Future development will focus on complex packaging materials for long‐term durability, hybrid energy storage technologies, and intelligent power management systems to ensure reliable, continuous operation.

## 8 Discussion and challenges

Despite tremendous advances in piezoelectric materials for wearable and implantable biomedical devices, numerous critical challenges remain unsolved. The fundamental problem is mimicking human skin characteristics, including flexibility, stretchability, and multimodal response, which are required for long‐term biocompatibility and integration with biological tissues. Despite having high piezoelectric coefficients, inorganic piezoelectric materials are often rigid, brittle, and even toxic, which limits their usage in flexible or implantable devices. Organic piezoelectric polymers, such as PVDF, PAN, and PLLA, provide more flexibility and biocompatibility; nevertheless, they have lower energy conversion efficiency and limited endurance under physiological conditions. Biodegradable and bioderived piezopolymers are ecologically friendly solutions for temporary implants; nevertheless, their efficiency varies greatly depending on production methods and ambient circumstances. A major difficulty in all material categories is striking a compromise between excellent piezoelectric performance, mechanical flexibility, and biodegradability. Furthermore, integrating many functionalities—such as sensing, energy harvesting, and actuation—into a single, small, and autonomous platform remains a considerable technological barrier.

Currently, scalable synthesis methods are being developed to ensure consistent dipole alignment and material quality. To fully realize the potential of piezoelectric devices in next‐generation healthcare solutions, more research must address systemic issues such as power management, signal integrity, and adaptability to changing biological conditions.

The development of manufacturing and characterization technologies has advanced the use of piezoelectric materials in bioelectronics, allowing for the creation of flexible, small, and high‐performance wearable and implantable devices. Fabrication processes such as electrospinning, spin coating, additive manufacturing, screen printing, and inkjet printing provide significant scalability, structural accuracy, and compatibility with flexible substrates. Irregular dipole alignment, limited resolution, material compatibility, and mechanical durability continue to be significant challenges in physiological applications. Furthermore, combining many functionalities—such as sensing, actuation, and energy harvesting—into a single device sometimes involves sophisticated hybrid manufacturing techniques, increasing both costs and processing complexity.

Piezoelectric force microscopy (PFM) and differential scanning calorimetry (DSC) are important characterization methods for examining the electromechanical and thermal capabilities of piezoelectric materials. PFM provides nanoscale insights into the polarization amplitude and direction, whereas DSC aids in the detection of the phase transitions required for performance. However, both technologies have limitations: PFM requires accurate calibration and is sensitive to ambient conditions, whereas DSC might be deceived by overlapping heat episodes and material‐specific variables. The absence of set restrictions, as well as the necessity for specialized equipment, limits widespread usage. Future research should focus on developing integrated, high‐throughput, and widely accessible manufacturing and characterization systems to ensure consistent performance and scalability for clinical and commercial applications.

The integration of piezoelectric materials into wearable electronics has revolutionized real‐time physiological monitoring by enabling self‐powered, flexible, and highly sensitive health devices. These systems can continuously track vital signs such as heart rate, blood pressure, respiratory patterns, and body motion, offering noninvasive, real‐time insights into a user's health status. Recent innovations, such as PLLA/Gly‐based biodegradable sensors and PAN/MXene/PDA@ZnO nanofiber composites, have demonstrated impressive sensitivity, fast response times, and mechanical durability. Moreover, advanced designs such as the PZT‐based wearable blood pressure sensor (WPBPS) have achieved clinical‐grade accuracy, validating their potential for medical diagnostics. However, several challenges remain. Ensuring long‐term biocompatibility and mechanical stability under repeated deformation is critical for reliable performance. The trade‐off between sensitivity and durability, especially in biodegradable systems, must be carefully managed. Additionally, achieving consistent signal quality in dynamic, real‐world conditions, such as during exercise or sleep, requires improved materials and device architectures. Power management, data transmission, and integration with wireless systems also pose engineering hurdles. Finally, large‐scale manufacturing and regulatory approval processes must be streamlined to transition these promising technologies from lab prototypes to commercial healthcare solutions.

The integration of piezoelectric materials into smart fabrics, e‐skins, and energy harvesting devices has opened new frontiers in wearable technology, enabling multifunctional systems that are lightweight, flexible, and capable of self‐powering. Smart textiles embedded with piezoelectric fibers can monitor physiological signals, detect motion, and even assist in therapeutic applications such as photodynamic therapy. These fabrics offer real‐time feedback for fitness, rehabilitation, and clinical diagnostics while maintaining user comfort and adaptability. Similarly, energy harvesting devices utilizing piezoelectric materials can convert biomechanical energy from daily activities into usable electrical power, reducing reliance on batteries and increasing the autonomy of wearable electronics. Innovations such as ultraflexible ferroelectric polymer transducers and hybrid nanofiber composites have demonstrated promising performance in terms of sensitivity, power density, and integration with wireless systems.

However, several challenges must be addressed to fully realize the potential of these technologies. The mechanical durability of piezoelectric materials under repeated deformation, washing, and long‐term wear remains a significant concern, particularly for textile‐based applications. Ensuring consistent performance while maintaining breathability and comfort is another critical hurdle. In energy harvesting systems, optimizing the balance between flexibility, energy output, and miniaturization is complex, especially when integrated with compact, body‐conforming devices. Additionally, scalable manufacturing processes that maintain material uniformity and device reliability are still under development. Future research should focus on enhancing material resilience, developing robust encapsulation strategies, and improving the integration of energy storage and wireless communication modules to create fully autonomous, multifunctional wearable systems.

The emergence of biodegradable and transient piezoelectric electronics represents a transformative step toward sustainable, self‐powered implantable medical devices. These systems offer significant advantages by eliminating the need for surgical removal and reducing electronic waste, making them ideal for short‐term therapeutic and diagnostic applications. Recent innovations, such as ultrasound‐driven biodegradable nanogenerators and piezoelectric scaffolds for nerve and spinal cord regeneration, have demonstrated the potential of materials such as KNN, PLLA, and PHBV to deliver localized electrical stimulation and support tissue healing. Additionally, the development of smart fabrics and nanofiber‐based sensors for in vivo monitoring highlights the versatility of piezoelectric materials in transient biomedical platforms. Self‐powered implants, including ferroelectric PLA‐based transducers and electroacoustic devices, further expand the scope of biodegradable electronics into areas such as speech recognition and AI‐integrated health monitoring.

Despite these promising advancements, several challenges remain. Achieving precise control over degradation rates in physiological and natural environments is critical to ensure device functionality during the intended therapeutic window. Material performance must be maintained under dynamic biological conditions, including mechanical stress, moisture, and temperature fluctuations. Furthermore, integrating energy harvesting, sensing, and wireless communication into a single biodegradable platform requires sophisticated material engineering and system design. The scalability of fabrication methods and the regulatory approval of transient materials for clinical use also present significant hurdles. Future research should focus on enhancing the mechanical robustness, electrical output, and biocompatibility of biodegradable piezoelectric materials while developing standardized protocols for in vivo performance evaluation and degradation profiling.

## 9 Future Trends and Directions

The future directions of piezoelectric materials in wearable and implantable biomedical devices reveal a dynamic and interdisciplinary landscape poised to transform healthcare technologies. One of the most significant trajectories is the development of multifunctional hybrid materials. These materials aim to combine the superior piezoelectric properties of inorganic ceramics with the flexibility, biocompatibility, and biodegradability of organic polymers. Future research will likely focus on optimizing the interface between these components to enhance their mechanical integrity and electrical performance, enabling devices that are both high‐performing and safe for long‐term implantation.

Another critical direction is the advancement of fabrication technologies. Techniques such as 3D printing, electrospinning, and inkjet printing will evolve to support the precise, scalable, and cost‐effective production of complex device architectures. These methods can be tailored to accommodate patient‐specific geometries and integrate multiple functionalities, such as sensing, actuation, and energy harvesting, into a single, compact platform. Additionally, self‐assembly and nanostructuring techniques will be explored to improve dipole alignment and enhance the piezoelectric response at the nanoscale.

The integration of smart electronics and wireless communication is also a key trend. Future piezoelectric systems will be embedded with low‐power electronics for real‐time data acquisition, processing, and transmission. These systems operate autonomously, are powered by harvested biomechanical energy, and communicate wirelessly with external devices or cloud‐based platforms. This will enable closed‐loop therapeutic systems that can monitor physiological signals and deliver targeted interventions in real time, significantly improving patient outcomes.

In parallel, the field is moving toward biodegradable and transient electronics for short‐term medical applications. These devices naturally dissolve in the body after their function is complete, eliminating the need for surgical removal and reducing medical waste. Research will focus on fine‐tuning degradation rates, ensuring predictable performance during the device's operational window, and developing biocompatible encapsulation materials that protect the device while allowing controlled breakdown.

Standardization and regulatory alignment will become increasingly important as these technologies move closer to clinical use. Establishing universal testing protocols, safety benchmarks, and performance metrics will be essential for gaining regulatory approval and ensuring patient safety. This will also facilitate interoperability between devices and healthcare systems, supporting broader adoption.

Moreover, the integration of artificial intelligence (AI) and Internet of Things (IoT) technologies will enable predictive analytics, personalized medicine, and remote patient monitoring. Piezoelectric devices serve as the sensory backbone of these intelligent systems, continuously collecting data that can be analyzed to detect early signs of disease, monitor treatment efficacy, and adapt therapeutic strategies in real time.

Finally, environmental sustainability is a growing concern. Future research will prioritize the development of eco‐friendly materials and manufacturing processes, aiming to reduce the environmental footprint of medical electronics. This includes exploring green synthesis methods, recyclable components, and energy‐efficient production techniques.

In summary, the future of piezoelectric biomedical devices lies in the convergence of material science, electronics, data science, and clinical medicine. By addressing current limitations and embracing emerging technologies, the next generation of piezoelectric systems will be smarter, safer, and more sustainable, paving the way for transformative advances in personalized and preventive healthcare.

## 10 Conclusion

Piezoelectric materials have become fundamental in advancing next‐generation wearable and implantable devices, providing distinctive energy harvesting, sensing, and actuation functionalities. This study highlights notable advancements in the synthesis, characterization, and application of organic and inorganic piezoelectric materials. Despite problems such as material toxicity, mechanical fragility, and fabrication scalability, current research is addressing these limits with creative material designs and better processing techniques.

The future of piezoelectric technology depends on its capacity for smooth integration into intricate biomedical systems while ensuring environmental and biological compatibility. Lead‐free and hybrid piezoelectric materials, along with improvements in production techniques, are poised to address existing constraints and expand application possibilities. Furthermore, the emergence of biodegradable materials and AI‐powered sensing technologies marks a pivotal period in customized healthcare. In conclusion, the ongoing development and incorporation of piezoelectric materials into wearable and implantable systems present significant potential for enhancing global health monitoring technology. The area is positioned to deliver innovative contributions to sustainable and adaptive healthcare solutions by tackling current difficulties and utilizing emerging trends.

## Conflict of Interest

The authors declare no conflict of interest.

## Author Contributions

B.K. contributed to conceptualization, original draft preparation, reviewing and editing, validation, and visualization. U.A. and B.K. were involved in original draft preparation as well as reviewing and editing. W.U.K., R.u.S.A., and M.S.K. contributed to reviewing and editing. M.E.H. was responsible for reviewing and editing, and supervision. B.L.K. contributed to conceptualization, reviewing and editing, visualization, validation, and supervision.
